# Unraveling the causal web of 4 adiposity indices and 92 multi-system outcomes: A body-wide Mendelian randomization study

**DOI:** 10.1097/MD.0000000000048986

**Published:** 2026-05-22

**Authors:** Xiaohong Chen, Chuyao Xiang, Long Xia, Qian Li, Chao Chen

**Affiliations:** a Department of Emergency, The Third People’s Hospital of Chengdu, Chengdu, Sichuan, China; b Department of Anesthesiology, Renji College, Wenzhou Medical University, Wenzhou, Zhejiang, China; c Department of Cardiology, The Third People’s Hospital of Chengdu, Chengdu, Sichuan, China; d Department of Thyroid Surgery, Sichuan Provincial People’s Hospital, University of Electronic Science and Technology of China, Chengdu, Sichuan, China.

**Keywords:** body mass index, hip circumference, Mendelian randomization analysis, total fat percentage, waist circumference

## Abstract

Body mass index (BMI) reflects general adiposity, waist circumference (WC) and hip circumference (HC) indicate regional fat distribution, and total fat percentage (TFP) represents overall body fat composition, while their independent disease-risk contributions remain unclear. Therefore, this study aimed to investigate the comparative and independent genetically predicted effects of 4 adiposity indices (BMI, WC, HC, and TFP) on 92 obesity-related outcomes. Two‑sample MR (TSMR) primarily employed the inverse-variance weighted (IVW) method, with MR‑Egger and maximum likelihood for supplementary analyses; multivariable MR (MVMR) applied the IVW and MR‑Egger methods. Sensitivity analyses were conducted to assess pleiotropy, heterogeneity, and outliers. All exposure datasets had sample sizes exceeding 300,000. Exposure and outcome data were obtained from large public consortia, including FinnGen and UK Biobank; most participants were of European ancestry, and there was no sample overlap between exposure and outcome datasets. TSMR using the IVW method (Bonferroni‑corrected *P* < .0001) demonstrated stronger positive associations between BMI and WC (18/92 and 21/92 outcomes, respectively) than HC (n = 13) and TFP (n = 9). MVMR using the IVW method revealed distinct independent causal associations between the different adiposity indices and diseases. BMI was independently and positively associated with a diverse range of health outcomes, including cardiometabolic (hypertension, and serum uric acid), respiratory (chronic obstructive pulmonary disease), gastrointestinal (gastroesophageal reflux disease [GERD]), musculoskeletal (osteoarthritis), and neuropsychiatric (sleep disorders and sleep apnea syndrome) diseases. WC was independently associated with an increased risk of multiple conditions, particularly cardiovascular and metabolic disorders (CMDs) and inflammatory conditions (e.g., atrial fibrillation, heart failure, hypertension, peripheral atherosclerosis, asthma, and cholecystitis). The independent causal effects of adiposity indices on outcomes remained robust even after separate adjustments for adipokines and inflammatory markers. However, TFP showed a positive association only with GERD among the 92 outcomes. Conversely, HC was negatively associated with the risk of multiple outcomes, notably CMDs. Results from MVMR using the IVW method and multivariable MR-Egger were consistent. Sensitivity analyses did not indicate substantial pleiotropy. The comprehensive MR analysis demonstrated the differential health effects of adiposity indices on multiple conditions. However, further studies are required to elucidate the underlying biological pathways.

## 1. Introduction

Obesity, clinically defined as a body mass index (BMI) ≥ 30 kg/m^2^, represents a critical global health challenge, implicated in millions of deaths and disabilities annually, and its prevalence continues to rise.^[1,2]^ However, relying solely on BMI as a screening measure has clear limitations, as it fails to capture critical aspects of body composition and fat distribution.^[3,4]^ This limitation is particularly evident in the “obesity paradox,” where studies have reported conflicting findings. For instance, while some evidence suggests that obesity reduces the quality of life in older adults,^[5]^ other observational analyses indicate that overweight and mild obesity (as defined by BMI) may be inverse associations with adverse outcomes in this population.^[6,7]^

This contradiction underscores the need for more precise adiposity indices that can better reflect health. Subsequent research has thus turned to measures such as waist circumference (WC), hip circumference (HC), and total fat percentage (TFP). For example, Liang et al found that a combination of lower WC, higher BMI, and higher HC was associated with better cognitive function in older people.^[8]^ Dang et al reported that WC was more strongly associated with cardiovascular disease than BMI,^[9]^ whereas Chen et al found BMI was more strongly associated with hypertension than WC.^[10]^ In addition, TFP has been positively associated with obstructive sleep apnea.^[11]^ Collectively, these studies suggest that different adiposity indices may have distinct – and sometimes opposing – associations with specific diseases. Therefore, universally advising weight reduction based solely on BMI may be an inappropriate and oversimplified clinical strategy.

However, these findings, generated from observational studies, lack sufficient statistical power to establish causal relationships owing to unavoidable confounding factors.^[12]^ Mendelian randomization (MR) analysis is a powerful tool for investigating causal associations using genetic variants as instrumental variables.^[13]^ However, most existing MR studies have relied on two-sample MR (TSMR), which lacks power to directly compare the suitability of different adiposity indices for specific diseases.^[14,15]^ Multivariable MR (MVMR) analysis is a powerful causal inference tool that identifies independent risk factors while simultaneously accounting for relationships among multiple covariates.^[16]^ In a previous MVMR analysis of 3 adiposity indices and infectious diseases, Chen et al found WC was a superior risk factor for several infections.^[17]^ However, the study was limited in scope and sample size.

To address these gaps, we conducted a comprehensive MVMR analysis using substantially larger datasets to systematically evaluate the causal roles of multiple adiposity indices across a wide range of diseases. This study aimed to clarify the differential relevance of specific adiposity measures for distinct disease outcomes, thereby providing evidence that may inform future risk assessment strategies and public health guidelines.

## 2. Materials and methods

To strengthen the reporting of observational epidemiological studies using MR guidelines, we employed a three-step MR approach to systematically investigate causal relationships between various adiposity indices and 92 diverse outcomes.^[18]^

### 2.1. Data sources

Four adiposity indices were selected as the exposures. These included BMI (kg/m^2^), which is the most commonly used measure for to identify obesity. TFP (%) was used to reflect the overall amount of fat in the body.^[19]^ WC (cm) and HC (cm) were chosen to examine whether fat distribution in specific regions had varying effects on particular outcomes.

The exposure and outcome datasets were derived from the well-known open consortiums. BMI, WC, and HC were obtained from the Neale Lab (sample sizes: 336,107, 336,639, and 336,601, respectively)^[20]^ and the European Bioinformatics Institute (sample sizes: 359,983, 407,661, and 407,662, respectively).^[21]^ TFP was obtained from the Neale Lab (sample size: 331,113). Detailed exposure information is provided in Table S1.

Ninety-two outcomes were categorized into 4 groups: cardiovascular and metabolic disorders (CMDs) (n = 25), immunity, infection, and reproductive disorders (n = 22), neuromusculoskeletal and mental health disorders (n = 21), and respiratory, digestive, and other disorders (n = 24). These 92 outcomes were selected to encompass a broad spectrum of diseases and physiological traits potentially influenced by adiposity, based on their established or suspected associations with obesity in epidemiological studies.^[22,23]^ Outcomes were mainly obtained from the FinnGen biobank (e.g., heart failure), medical research council integrative epidemiology unit (e.g., colon cancer), and other consortiums (e.g., the UK Biobank).^[24]^ Most of the study participants were of European ancestry. The details of the outcomes are listed in Table S2. No sample overlap was observed between the exposure and outcome genome-wide association studies (GWAS) datasets. All data were obtained from the GWAS summary statistics (https://gwas.mrcieu.ac.uk/).

### 2.2. Study design

**Figure 1.**
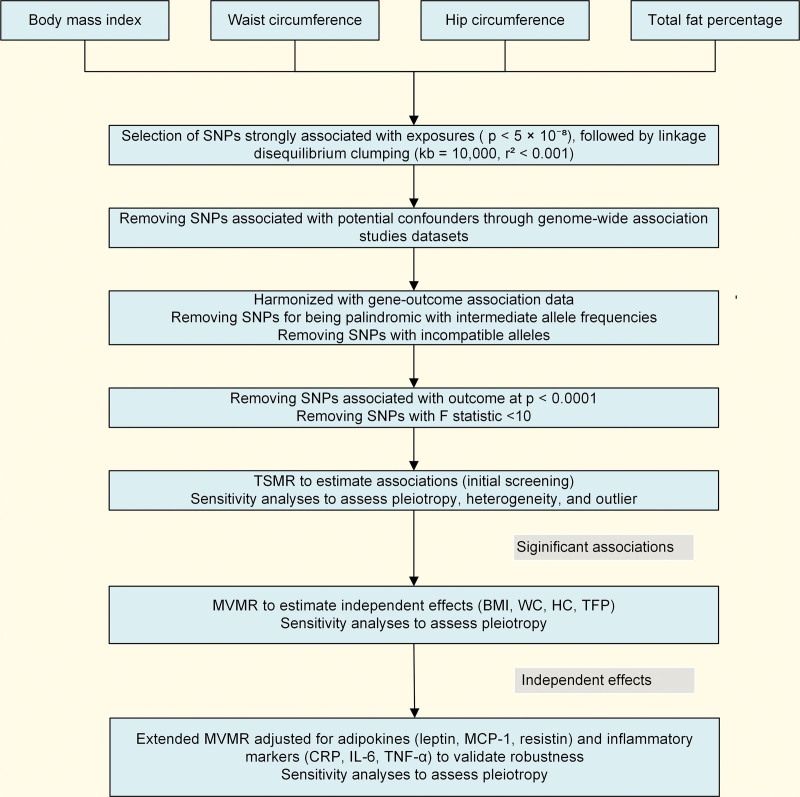
Analytical strategy. Analytical strategy with a three-step MR workflow: TSMR analyses for initial screening; MVMR analyses adjusted for core adiposity indices (BMI, WC, HC, TFP); and Extended MVMR with additional adjustment for adipokines (leptin, MCP-1, resistin) and inflammatory markers (CRP, IL-6, and TNF-α). BMI = body mass index, CRP = C-reactive protein, HC = hip circumference, IL-6 = interleukin-6, MCP-1 = monocyte chemoattractant protein-1, MR = Mendelian randomization, MVMR = multivariable MR, TFP = total fat percentage, TNF-α = tumor necrosis factor-alpha, TSMR = two-sample MR, WC = waist circumference.

A three-step MR framework was used to systematically evaluate the causal relationships between 4 adiposity indices (BMI, WC, HC, TFP) and 92 outcomes (Fig. 1).

First, TSMR was performed to estimate the total effects of each adiposity index on all 92 outcomes. To account for multiple testing, a Bonferroni-corrected significance threshold of *P* < .0001 (α = 0.05/368, 4 exposures and 92 outcomes) was applied.^[25]^ Outcomes that showed a significant association with at least one adiposity index at this threshold were selected for subsequent MVMR analyses.

Second, MVMR was performed to estimate the independent effect of each adiposity index on the outcomes selected from TSMR, while simultaneously accounting for the other 3 indices. For any outcome where an adiposity index showed a nominally significant independent association (*P* < .05), we proceeded to the third step to assess the robustness of these associations.

Third, extended MVMR was performed as a supplementary method to test the robustness of the independent associations observed in the second step. This analysis was conducted by separately adjusting for adipokines and inflammatory markers, considering that adipose tissue functions as an endocrine organ secreting adipokines^[26]^ and plays a crucial role in inflammatory responses.^[27]^ The adipokines included leptin, monocyte chemoattractant protein-1, and resistin, whereas the inflammatory markers included C-reactive protein (CRP), interleukin-6, and tumor necrosis factor-alpha. Detailed information on adipokines and inflammatory markers is presented in Table S3.

For binary outcomes, effect estimates are presented as odds ratios (OR) with 95% confidence intervals (CI). For continuous outcomes, effect estimates are presented as beta coefficients (β) with 95% CI. All effect estimates correspond to a 1‑standard deviation increase in the exposure.

### 2.3. Instrument selection

Analyses followed the 3 core MR assumptions^[13]^: Relevance assumption, single nucleotide polymorphisms (SNPs) strongly associated with exposures (*P* < 5 × 10^−8^) and independent by linkage disequilibrium (kb = 10,000, *r*^2^ < 0.001) were selected.^[28]^ Weak instruments (*F* < 10) were excluded,^[29]^ $F=\frac{N-K-1}{k}\times \frac{R^{2}}{1-R^{2}}$, where *R*^2^ is the quantity of instrumental variables representing exposure, *k* is the number of SNPs, and *N* is the exposure sample size^[30]^; Independence assumption, SNPs related to potential confounders in GWAS datasets were excluded. Potential confounders included lifestyle factors, cardiometabolic disorders, respiratory diseases, psychiatric conditions, and other relevant traits. A comprehensive list of the specific confounders checked for each exposure-outcome pair is provided in Table S4; and Exclusion assumption, SNPs associated with outcomes (*P* < .0001) were removed. This threshold lies between the conventional nominal significance level (*P* < .05) and the genome-wide significance level (*P* < 5 × 10^−8^), striking a balance between minimizing horizontal pleiotropy risk and preserving statistical power, which is consistent with current guidelines for MR investigations.^[31]^

### 2.4. MR analysis

The inverse-variance weighted (IVW) method was used as the main analytical approach for TSMR analysis, as it yields the most precise causal estimates under the assumption of valid instrumental variables.^[32]^ A random-effects model was applied when significant heterogeneity was detected; otherwise, a fixed-effects model was used.^[33]^ The MR-Egger and maximum likelihood methods were performed as supplementary analyses. The maximum likelihood approach offers advantages in scenarios with weak instruments and provides unbiased estimates.^[34]^ The MR-Egger intercept test was used to assess directional pleiotropy, and Cochran *Q* statistic was applied to evaluate heterogeneity, while the MR pleiotropy residual sum and outlier test was implemented to identify potential outliers.^[35]^ MVMR-IVW was used as the primary analytical method for MVMR to estimate independent effects of each adiposity index while accounting for correlations among exposures. The MVMR-Egger test was also employed as a supplementary approach to evaluate potential pleiotropy.^[35]^

All statistical analyses and partial visualization were performed in R (version 4.4.1) using the “TwoSampleMR,” “MRPRESSO,” and “MendelianRandomization” packages. Additional figures were generated using OriginPro 2025 (version 10.2.0.196).

### 2.5. Ethics approval

Publicly available summary statistics were obtained from GWAS datasets via the Open database (https://gwas.mrcieu.ac.uk/). As these data derive from published studies with existing ethical approval and participant consent, no additional ethics approval was required.

## 3. Results

### 3.1. Two-sample Mendelian randomization results

**Figure 2.**
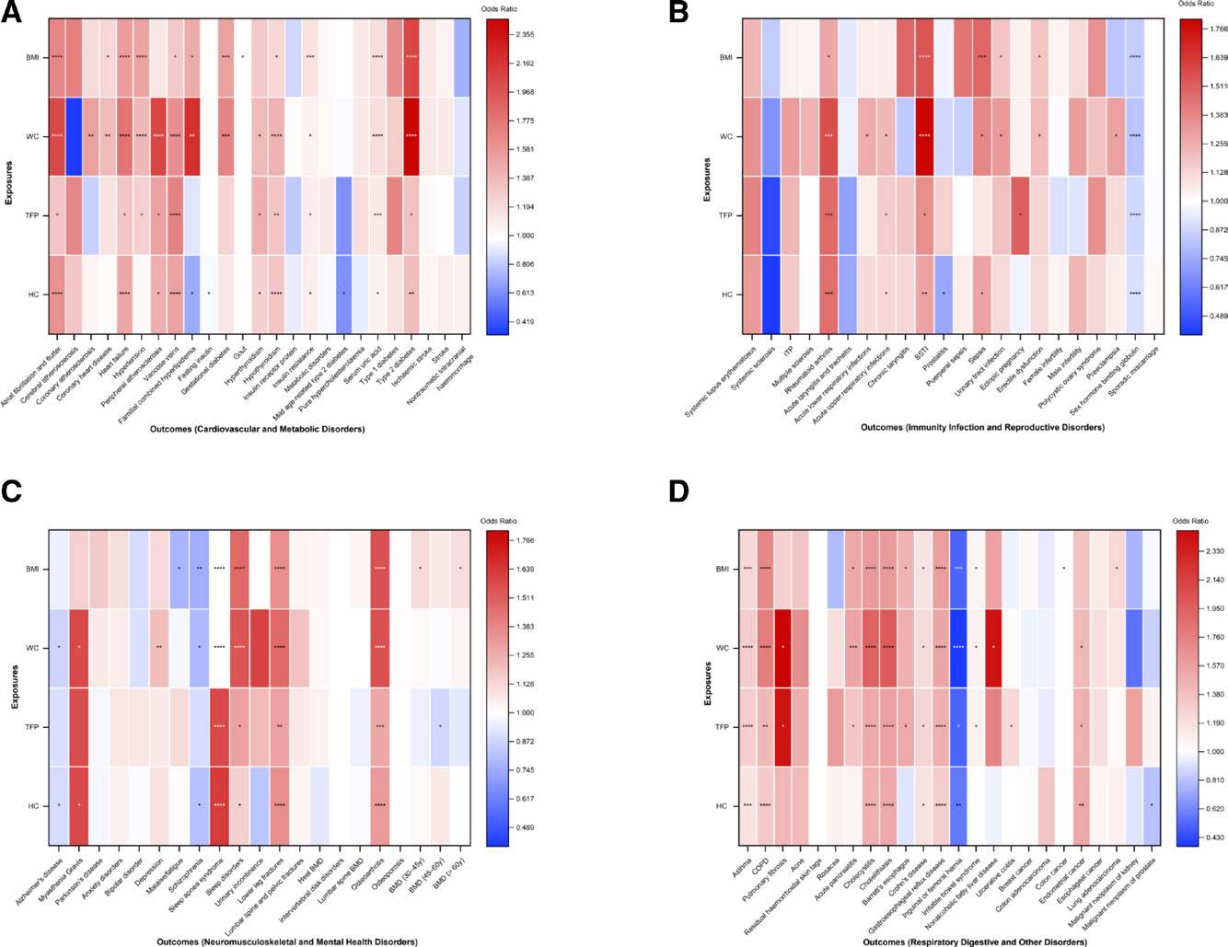
Two-sample Mendelian randomization analysis of 4 adiposity indices on 92 outcomes. TSMR analysis of the 4 adiposity indices on 92 outcomes. (A) Cardiovascular and metabolic disorders. (B) Immunity infection and reproductive disorders. (C) Neuromusculoskeletal and mental health disorders. (D) Respiratory digestive and other disorders. Significance levels: *P* ≥ .05 (no symbol), * .001 ≤ *P* < .05, ** .0001 ≤ *P* < .001, *** .00001 ≤ *P* < .0001, **** *P* < .00001. For binary outcomes, effect estimates are presented as odds ratios (OR) with 95% confidence intervals (CI). For continuous outcomes, the linear beta coefficients from the MR analyses were exponentiated and are presented as OR per 1-standard deviation increase in the exposure, allowing a consistent visual representation across all outcomes. BMD = bone mineral density, COPD = chronic obstructive pulmonary disease, ITP = idiopathic thrombocytopenic purpura, SSTI = infections of the skin and subcutaneous tissue, TSMR = two-sample MR.

Using the IVW method (Bonferroni‑corrected *P* < .0001), we identified significant positive associations between the 4 adiposity indices and 92 outcomes (Table S5, Fig. 2A–D).

BMI was positively associated with 18 outcomes. The strongest associations were observed for sleep apnea syndrome (OR: 2.192, 95% CI: 1.735**–**2.768; *P* = 4.41 × 10^−11^), type 2 diabetes (OR: 2.059, 95% CI: 1.751**–**2.421; *P* = 2.54 × 10^−18^), chronic obstructive pulmonary disease (COPD) (OR: 1.708, 95% CI: 1.491**–**1.957; *P* = 1.23 × 10^−14^), osteoarthritis (OR: 1.554, 95% CI: 1.460**–**1.654; *P* = 8.42 × 10^−44^), heart failure (OR: 1.548, 95% CI: 1.313**–**1.825; *P* = 2.07 × 10^−7^), and hypertension (OR: 1.519, 95% CI: 1.297**–**1.780; *P* = 2.13 × 10^−7^). Additional associations included atrial fibrillation and flutter (AF/AFL), gestational diabetes, insulin resistance, gastroesophageal reflux disease (GERD), serum uric acid (SUA), asthma, cholecystitis, cholelithiasis, infections of the skin and subcutaneous tissue (SSTI), sepsis, sleep disorders, and fracture of the lower leg including ankle.

WC showed the broadest causal associations, being positively associated with 21 outcomes. These associations were predominantly concentrated in CMDs and inflammatory conditions. Among CMDs, WC was associated with type 2 diabetes (OR: 2.451, 95% CI: 2.050–2.931; *P* = 9.07 × 10^−23^), peripheral atherosclerosis (OR: 2.056, 95% CI: 1.539–2.746; *P* = 1.06 × 10^−6^), AF/AFL (OR: 2.043, 95% CI: 1.632–2.557; *P* = 4.47 × 10^−10^), heart failure (OR: 1.799, 95% CI: 1.465–2.210; *P* = 2.13 × 10^−8^), and hypertension (OR: 1.397, 95% CI: 1.183–1.649; *P* = 8.13 × 10^−5^). Other CMDs included gestational diabetes, varicose veins, hypothyroidism, and SUA. F OR: inflammatory conditions, WC was associated with asthma (OR: 1.301, 95% CI: 1.189–1.423; *P* = 9.34 × 10^−9^), cholecystitis (OR: 1.934, 95% CI: 1.597–2.343; *P* = 1.52 × 10^−11^), cholelithiasis (OR: 1.978, 95% CI: 1.628–2.402; *P* = 6.35 × 10^−12^), COPD (OR: 1.773, 95% CI: 1.565–2.008; *P* = 2.08 × 10^−19^), and acute pancreatitis (OR: 1.532, 95% CI: 1.258–1.866; *P* = 2.17 × 10^−5^). Additional associations included SSTI, rheumatoid arthritis, osteoarthritis, and GERD.

HC was positively associated with 13 outcomes. The strongest associations were observed for: sleep apnea syndrome (OR: 1.628, 95% CI: 1.440**–**1.840; *P* = 6.86 × 10^−15^), AF/AFL (OR: 1.608, 95% CI: 1.336**–**1.935; *P* = 4.89 × 10^−7^), varicose veins (OR: 1.533, 95% CI: 1.310**–**1.794; *P* = 9.77 × 10^−8^), cholelithiasis (OR: 1.520, 95% CI: 1.296**–**1.782; *P* = 2.51 × 10^−7^), and heart failure (OR: 1.500, 95% CI: 1.299**–**1.733; *P* = 3.53 × 10^−8^). Additional associations included cholecystitis, hypothyroidism, COPD, fracture of the lower leg including ankle, osteoarthritis, GERD, asthma, and sleep disorders.

TFP was positively associated with 9 outcomes. The strongest associations were observed for: varicose veins (OR: 1.708, 95% CI: 1.409–2.070; *P* = 4.90 × 10^−8^), sleep apnea syndrome (OR: 1.586, 95% CI: 1.358–1.853; *P* = 5.92 × 10^−9^), cholelithiasis (OR: 1.559, 95% CI: 1.298–1.871; *P* = 1.92 × 10^−6^), and cholecystitis (OR: 1.509, 95% CI: 1.266–1.800; *P* = 4.60 × 10^−6^). Additional associations included GERD, asthma, COPD, osteoarthritis, and SUA.

All of these results were consistent with the maximum likelihood estimates. Some heterogeneity was observed in TSMR analyses; however, among 368 tests, significant pleiotropy was detected only for the association between WC and sex hormone-binding globulin (*P* < .05), and MR pleiotropy residual sum and outlier identified outliers but no significant ones (Table S6).

### 3.2. Multivariable Mendelian randomization results

**Figure 3.**
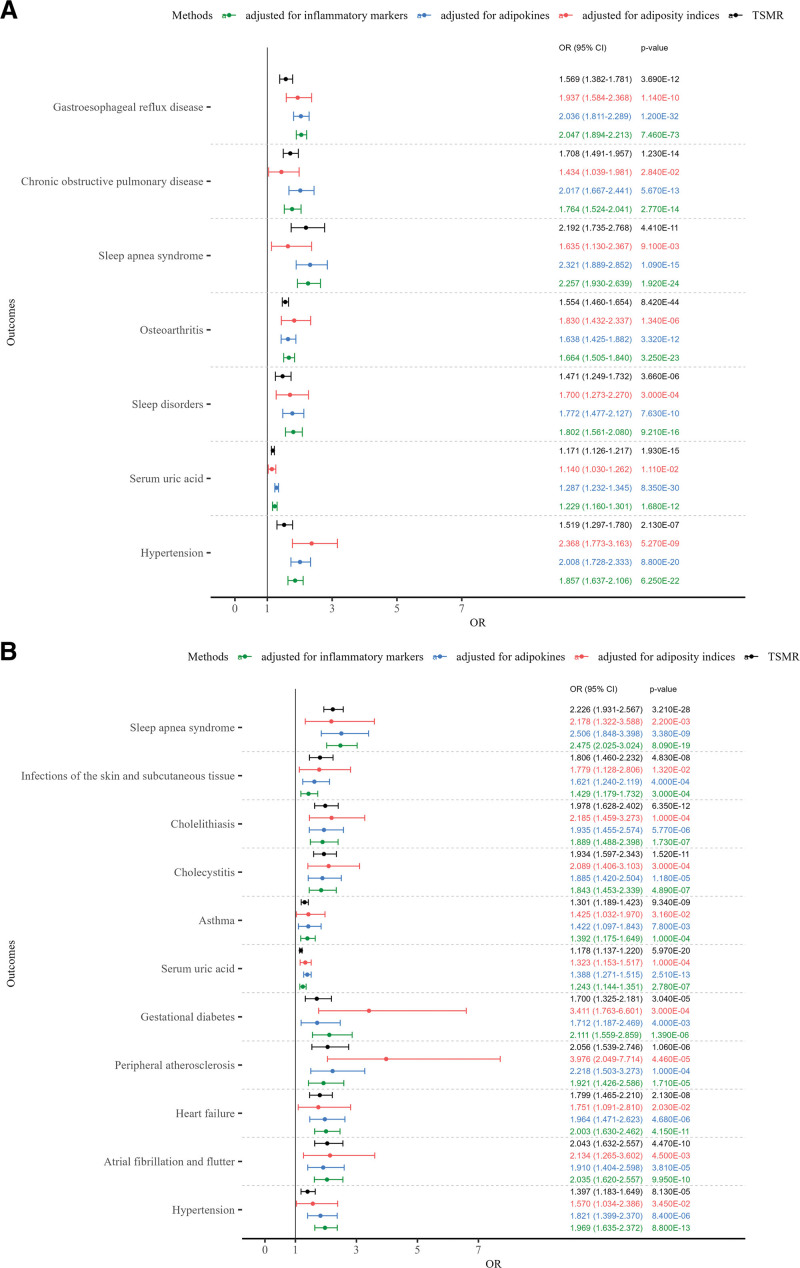
Independent effects of body mass index and waist circumference on outcomes. Forest plots showing effect estimates from MVMR-IVW method. For binary outcomes, effect estimates are presented as odds ratios (OR) with 95% confidence intervals (CI). For continuous outcomes, the linear beta coefficients from the MR analyses were exponentiated and are presented as OR per 1-standard deviation increase in the exposure, allowing a consistent visual representation across all outcomes. (A) BMI for outcomes and (B) WC for outcomes. Significant associations identified in TSMR (*P* < .0001) were further tested in 3 MVMR models: adjusted for other adiposity indices (BMI, WC, HC, TFP), adjusted for adipokines (leptin, MCP-1, and resistin), and adjusted for inflammatory markers (CRP, IL-6, and TNF-α). BMI = body mass index, CRP = C-reactive protein, HC = hip circumference, IL-6 = interleukin-6, IVW = inverse-variance weighted, MCP-1 = monocyte chemoattractant protein-1, MR = Mendelian randomization, MVMR = multivariable MR, TFP = total fat percentage, TNF-α = tumor necrosis factor-alpha, TSMR = two-sample MR, WC = waist circumference.

After mutually adjusting for all 4 adiposity indices using the MVMR-IVW method, distinct independent causal patterns emerged (Table S7, Fig. 3A,B).

BMI remained independently and positively associated with 7 outcomes. The strongest associations were observed for hypertension (OR: 2.368, 95% CI: 1.773–3.163; *P* = 5.27 × 10^−9^), GERD (OR: 1.937, 95% CI: 1.584–2.368; *P* = 1.14 × 10^−10^), osteoarthritis (OR: 1.830, 95% CI: 1.432–2.337; *P* = 1.34 × 10^−6^), sleep disorders (OR: 1.700, 95% CI: 1.273–2.270; *P* = .0003), and sleep apnea syndrome (OR: 1.635, 95% CI: 1.130–2.367; *P* = .0091). Additional independent associations included COPD and SUA.

WC showed independent positive associations with 11 outcomes. The strongest effects were observed for peripheral atherosclerosis (OR 3.976, 95% CI: 2.049–7.714; *P* = 4.46 × 10^−5^), gestational diabetes (OR: 3.411, 95% CI: 1.763–6.601; *P* = .0003), cholelithiasis (OR: 2.185, 95% CI: 1.459–3.273; *P* = .0001), sleep apnea syndrome (OR: 2.178, 95% CI: 1.322–3.588; *P* = .0022), and AF/AFL (OR: 2.134, 95% CI: 1.265–3.602; *P* = .0045). Additional independent associations included cholecystitis, SSTI, heart failure, hypertension, asthma, and SUA.

TFP was independently associated only with GERD (OR: 1.675, 95% CI: 1.389–2.020; *P* = 6.47 × 10^−8^), while HC was positively associated with varicose veins (OR: 1.862, 95% CI: 1.340–2.588; *P* = .0002) and hypothyroidism (OR: 1.449, 95% CI: 1.107–1.895; *P* = .0069). The patterns were largely consistent in the MVMR-Egger analyses (except for BMI on COPD, *P* = .0824). Pleiotropy was observed only in the adiposity-GERD association (*P* = .0005) (Table S7). These results remained stable in the extended MVMR analyses after adjusting for adipokines (leptin, MCP‑1, and resistin) (Table S8) and inflammatory markers (CRP, IL‑6, and TNF‑α) (Table S9).

**Figure 4.**
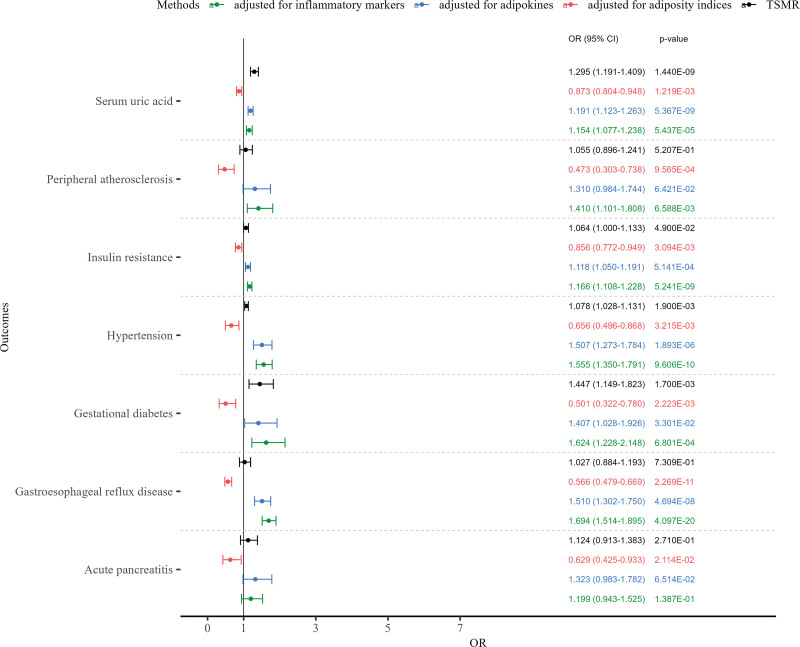
Inverse associations of hip circumference with selected outcomes. Forest plots showing effect estimates from MVMR-IVW method. For binary outcomes, effect estimates are presented as odds ratios (OR) with 95% confidence intervals (CI). For continuous outcomes, the linear beta coefficients from the MR analyses were exponentiated and are presented as OR per 1-standard deviation increase in the exposure, allowing a consistent visual representation across all outcomes. The outcomes shown are those for which HC exhibited inverse associations after adjusting for the other 3 adiposity indices. HC = hip circumference, IVW = inverse-variance weighted, MR = Mendelian randomization, MVMR = multivariable MR.

However, after adjusting for the other 3 adiposity indices (MVMR-IVW method), HC showed inverse associations with acute pancreatitis, GERD, gestational diabetes, hypertension, insulin resistance, peripheral atherosclerosis, and SUA, but these associations reversed to increased risks after further adjustment for adipokines and inflammatory markers (Fig. 4).

## 4. Discussion

This MVMR analysis showed that fat distribution exhibited stronger and more consistent causal associations across the 92 outcomes than overall fat mass. While the overall adiposity index BMI was linked to a broad but heterogeneous set of conditions, and TFP was an independent risk factor only for GERD, indices of fat distribution, specifically WC (abdominal) and HC (gluteofemoral), showed the strongest and most specific links: WC robustly increased the risk for CMDs and inflammatory outcomes, whereas HC exhibited inverse associations.

Elevated WC exhibited the broadest causal associations, showing significant positive associations with 21 outcomes in TSMR, more than BMI (18 outcomes), HC (13 outcomes), and TFP (9 outcomes) (Fig. 2). Notably, while these associations were concentrated in CMDs and inflammatory conditions, the strongest independent effect in MVMR among CMDs was observed for peripheral atherosclerosis (CMD; OR: 3.976, 95% CI: 2.049–7.714; *P* = 4.46 × 10^−5^), and the strongest inflammatory association was seen for cholelithiasis (OR: 2.185, 95% CI: 1.459–3.273; *P* = .0001). These findings are consistent with prior studies showing that WC can unmask individuals at risk for CMDs who are classified as “normal-weight” by BMI.^[9]^ Elevated WC likely reflects excessive visceral adiposity, a condition associated with systemic and vascular inflammation that drives atherosclerosis.^[36]^

Regarding respiratory outcomes, among the 4 adiposity indices, only BMI was an independent risk factor for COPD, whereas only WC was an independent risk factor asthma. Both BMI and WC were associated with an increased risk of sleep apnea syndrome, while none of the indices showed significant associations with pulmonary fibrosis and respiratory infections. This divergence suggests distinct underlying mechanisms: abdominal adiposity (reflected by WC) may primarily exacerbate airway hyperresponsiveness through visceral fat-driven systemic inflammation (e.g., in asthma), whereas general body mass (reflected by BMI) exerts a profound mechanical and ventilatory burden that compromises lung mechanics (e.g., in COPD and sleep apnea), rather than directly causing parenchymal lung diseases.^[37–39]^

For musculoskeletal impacts, the MVMR analysis showed that BMI was the independent causal factor on osteoarthritis. However, neither BMI nor other adiposity indices were causally associated with bone mineral density, osteoporosis, or fractures. This musculoskeletal divergence highlights the complex dual nature of adiposity: while the mechanical burden of a higher BMI directly accelerates physical cartilage wear-and-tear in weight-bearing joints (driving osteoarthritis risk), this same mechanical loading also stimulates bone formation (according to Wolff Law),^[40]^ which counterbalances the detrimental effects of adiposity-driven systemic inflammation on bone microarchitecture. Consequently, the net causal effect on osteoporosis and fracture risk is nullified. Collectively, these findings suggest that general adiposity (BMI) acts as the primary driver for conditions governed by mechanical, ventilatory, and hemodynamic overload (such as osteoarthritis, COPD, and hypertension), distinguishing its role from that of the inflammation-driven effects of other adiposity indices.^[10,41]^

Within immune conditions, adiposity selectively exhibited significant positive associations with rheumatoid arthritis, but not with systemic lupus erythematosus, systemic sclerosis, and multiple sclerosis. This suggests that the causal impact of adiposity-driven metabolic inflammation may be more specific to synovial autoimmunity, rather than acting as a universal trigger for all systemic immune diseases. Consistent with this, obesity-driven elevations in interleukin-6 and tumor necrosis factor-alpha have been shown to specifically promote the recruitment of neutrophils and monocytes to the synovial microenvironment.^[42]^ However, no causal associations were observed between any adiposity indices and a broad spectrum of other conditions, including neurodegenerative diseases (e.g., Alzheimer), reproductive disorders (e.g., infertility), and various malignancies (e.g., breast, colon, and prostate cancers).

HC showed inverse associations with several outcomes after adjusting for other indices, particularly with CMDs (Fig. 4). Biologically, HC reflects subcutaneous adipose tissue, a depot characterized by metabolic plasticity and a beneficial capacity for “browning,” in contrast to the pathogenic profile of visceral fat.^[43]^ Although TFP emerged as an independent risk factor only for GERD, this does not imply that the total amount of fat is biologically irrelevant. Rather, the near-null findings for TFP across other outcomes likely reflect the attenuation of its effects after adjusting for BMI and regional fat indices, as well as potential measurement limitations of TFP and reduced instrument strength in our multivariable models.

Extended MVMR analyses incorporating inflammatory markers and adipokines provided deeper insights into the potential pathways of adiposity-associated risks, revealing a depot-specific and outcome-specific pattern. For CMDs and inflammatory conditions (e.g., peripheral atherosclerosis, heart failure, hypertension, asthma, and cholecystitis), the positive associations of WC and BMI were generally attenuated after adjusting for adipokines (Table S8) and inflammatory markers (Table S9). This attenuation suggests that the detrimental impacts of general and abdominal obesity on these conditions are partially explained by pro‑inflammatory pathways and dysregulated adipokine secretion. Conversely, for conditions fundamentally driven by mechanical stress, such as GERD, COPD, and sleep apnea syndrome, the direct effect estimates for BMI and WC slightly increased or remained robust after adjusting for these markers, reinforcing that physical obstruction, rather than systemic inflammation, primarily drives their pathogenesis.^[37,39,44]^ Most notably, for HC (Fig. 4), its inverse associations with outcomes including acute pancreatitis, GERD, gestational diabetes, hypertension, insulin resistance, peripheral atherosclerosis, and SUA were largely reversed after adjusting for inflammatory markers and adipokines. This reversal provides genetic evidence that the metabolic benefits of gluteofemoral fat are heavily dependent on its capacity to maintain a favorable adipokine profile and suppress systemic inflammation.

Individuals often present with multiple abnormal fat measurements (e.g., high WC and HC) alongside cardiometabolic multimorbidity. In the extended MVMR, adjusting for inflammatory and adipokine markers did not significantly attenuate the positive associations of abdominal adiposity (WC) with these outcomes, suggesting WC drives multimorbidity through multiple parallel pathways. Conversely, the initial inverse associations of gluteofemoral fat (HC) reversed to positive associations upon identical adjustments, indicating that HC’s inverse effects strictly depend on favorable inflammatory and adipokine profiles. Therefore, multimorbidity likely manifests when the multi-pathway toxicity of abdominal fat overwhelms the inflammation- and adipokine-dependent inverse effects of HC.

The specificity of our findings underscores the value of retaining distinct phenotypes. For example, WC was independently associated with sleep apnea syndrome (OR: 2.178, 95% CI: 1.322–3.588; *P* = .0022) but not with broader sleep disorders (OR: 1.501, 95% CI: 0.988–2.279; *P* = .0569) in MVMR analysis (Table S7), suggesting that abdominal adiposity may influence sleep disturbances through pathways related to respiratory or metabolic dysfunction rather than general sleep regulation. Similarly, WC and BMI were independently associated with SUA (Table S7), but none of the 4 adiposity indices were associated with gout (Table S5). These observations would have been masked had the outcomes been combined, highlighting the importance of granular phenotyping in adiposity research.

This study makes a key contribution to this field. First, it moves beyond the limitations of observational studies in establishing causal inference.^[12]^ Previous observational findings on adiposity are often inconsistent and confounded.^[45,46]^ By applying a comprehensive MR framework across 4 distinct adiposity indices and 92 outcomes, our analysis provides stronger genetic evidence and offers a new perspective for addressing these inconsistencies. Second, our analysis directly compared multiple adiposity indices to identify disease-specific causal drivers. Unlike prior MR studies that focused on a single adiposity index or limited health outcomes, our MVMR design directly compared different adiposity measures, pinpointing which obesity type is the primary causal driver for distinct outcomes.^[15,17]^ Moreover, our extended MVMR analysis revealed that inflammatory markers and adipokines can exert distinct biological effects depending on the tissue source.

Despite its strengths, this study had several limitations. First, the genetic data predominantly came from European ancestry populations, suggesting the need for validation in more diverse ethnic groups. Second, although all SNPs associated with potential confounders were removed to minimize pleiotropy, it could not be completely eliminated. Third, because GWAS data were primarily collected from cross-sectional studies, we could not assess how changes in adiposity indices affect the health outcomes of individuals. Therefore, future longitudinal observational studies are warranted.

## 5. Conclusions

A comprehensive MR analysis demonstrated the differential causal effects of various adiposity indices on multiple outcomes. Prospective real-world cohort studies are needed to validate these results and elucidate the underlying biological pathways.

## Acknowledgments

We thank all the participants for sharing the Genome-Wide association study datasets used in this study.

## Author contributions

**Conceptualization:** Xiaohong Chen, Chao Chen.

**Data curation:** Chuyao Xiang, Long Xia, Qian Li.

**Formal analysis:** Chuyao Xiang, Long Xia, Qian Li.

**Funding acquisition:** Xiaohong Chen.

**Methodology:** Xiaohong Chen, Chuyao Xiang, Long Xia, Qian Li, Chao Chen.

**Software:** Chuyao Xiang, Long Xia, Qian Li.

**Visualization:** Chuyao Xiang, Qian Li, Long Xia.

**Validation:** Long Xia.

**Writing – original draft:** Xiaohong Chen.

**Writing – review & editing:** Xiaohong Chen, Chao Chen.


















