# Supplementary material for: Unraveling the causal web of 4 adiposity indices and 92 multi-system outcomes: A body-wide Mendelian randomization study
Source: Medicine (Baltimore). 2026 May 22;105(21):e48986. doi: 10.1097/MD.0000000000048986 (PMC13201005; doi:10.1097/MD.0000000000048986)
Supplement: Supplementary file 1 [file medi-105-e48986-s001.docx]

Table S1. Profiles of exposures in Genome-Wide association study datasets.

| **Exposures** | **GWAS ID (exposures)** | **Year** | **Sample size** | **Consortium** | **Population** | **PMID** |
| --- | --- | --- | --- | --- | --- | --- |
| WC | ebi-a-GCST90014020 | 2021 | 407,661 | EBI | European | 34017140 |
|  | ukb-a-382 | 2017 | 336,639 | Neale Lab | European | NA |
| BMI | ebi-a-GCST90018947 | 2021 | 359,983 | EBI | European | 34594039 |
|  | ukb-a-248 | 2017 | 336,107 | Neale Lab | European | NA |
| HC | ebi-a-GCST90014021 | 2021 | 407,662 | EBI | European | 34017140 |
|  | ukb-a-388 | 2017 | 336,601 | Neale Lab | European | NA |
| TFP | ukb-a-290 | 2017 | 331,113 | Neale Lab | European | NA |

Note: Different exposure data sources were used based on outcome sources to avoid sample overlap between exposure and outcome populations.

Abbreviations: BMI, body mass index; HC, hip circumference; TFP, total fat percentage; WC, waist circumference; EBI, European bioinformatics institute.
