# Supplementary material for: Unraveling the causal web of 4 adiposity indices and 92 multi-system outcomes: A body-wide Mendelian randomization study
Source: Medicine (Baltimore). 2026 May 22;105(21):e48986. doi: 10.1097/MD.0000000000048986 (PMC13201005; doi:10.1097/MD.0000000000048986)
Supplement: Supplementary file 2 [file medi-105-e48986-s002.docx]

Table S2. Profiles of outcomes in genome-wide association studies datasets.

| **Outcomes** | **GWAS ID (outcomes)** | **Year** | **Sample size** | **nCase** | **nControl** | **Consortium** | **Population** | **PMID** |
| --- | --- | --- | --- | --- | --- | --- | --- | --- |
| **Cardiovascular and Metabolic Disorders (CMDs)** | | | | | | | | |
| Atrial fibrillation and flutter | finn-b-I9_AF | 2021 | 138994 | 22068 | 116926 | FinnGen biobank | European | NA |
| Coronary heart disease | finn-b-I9_CHD | 2021 | 218792 | 21012 | 197780 | FinnGen biobank | European | NA |
| Heart failure | finn-b-I9_HEARTFAIL | 2021 | 208178 | 13087 | 195091 | FinnGen biobank | European | NA |
| Hypertension | finn-b-I9_HYPTENSESS | 2021 | 205694 | 42857 | 162837 | FinnGen biobank | European | NA |
| Coronary atherosclerosis | finn-b-I9_CORATHER | 2021 | 211203 | 23363 | 187840 | FinnGen biobank | European | NA |
| Cerebral atherosclerosis | finn-b-I9_CERATHER | 2021 | 203172 | 104 | 203068 | FinnGen biobank | European | NA |
| Peripheral atherosclerosis | finn-b-DM_PERIPHATHERO | 2021 | 168832 | 6631 | 162201 | FinnGen biobank | European | NA |
| Varicose veins | finn-b-I9_VARICVE_EXNONE | 2021 | 218792 | 17027 | 201765 | FinnGen biobank | European | NA |
| Type 1 diabetes | ebi-a-GCST90013891 | 2021 | 407746 | / | / | EBI | European | 34017140 |
| Type 2 diabetes | finn-b-E4_DM2_STRICT | 2021 | 212351 | 29166 | 183185 | FinnGen biobank | European | NA |
| Gestational diabetes | finn-b-GEST_DIABETES | 2021 | 123579 | 5687 | 117892 | FinnGen biobank | European | NA |
| Mild age-related type 2 diabetes | ebi-a-GCST90026416 | 2021 | 5605 | 2861 | 2744 | EBI | European | 34737425 |
| Fasting insulin | ebi-a-GCST90002238 | 2021 | 151013 | / | / | EBI | European | 34059833 |
| Insulin resistance | ebi-a-GCST005179 | 2010 | 37037 | / | / | EBI | European | 20081858 |
| Insulin receptor protein | prot-a-1564 | 2018 | 3301 | / | / | NA | European | 29875488 |
| Hypothyroidism | ebi-a-GCST90013893 | 2021 | 405357 | / | / | EBI | European | 34017140 |
| Hyperthyroidism | ebi-a-GCST90018860 | 2021 | 460499 | 3557 | 456942 | EBI | European | 34594039 |
| Serum uric acid | ebi-a-GCST90018977 | 2021 | 343836 | / | / | EBI | European | 34594039 |
| Gout | ebi-a-GCST90038687 | 2021 | 484598 | 6810 | 477788 | EBI | European | 33959723 |
| Familial combined hyperlipidemia | ebi-a-GCST90104003 | 2021 | 349222 | 3838 | 345384 | EBI | European | 34906840 |
| Pure hypercholesterolaemia | finn-b-E4_HYPERCHOL | 2021 | 206067 | 8808 | 197259 | FinnGen biobank | European | NA |
| Metabolic disorders | finn-b-E4_METABOLIA | 2021 | 218792 | 21533 | 197259 | FinnGen biobank | European | NA |
| Ischaemic stroke | finn-b-I9_STR_EXH_EXNONE | 2021 | 218792 | 10551 | 208241 | FinnGen biobank | European | NA |
| Stroke | finn-b-I9_STR_SAH | 2021 | 212947 | 12632 | 200315 | FinnGen biobank | European | NA |
| Nontraumatic intracranial haemorrhage | finn-b-I9_INTRACRA | 2021 | 205862 | 2794 | 203068 | FinnGen biobank | European | NA |
| **Neuromusculoskeletal and Mental Health Disorders (NMMDs)** | | | | | | | | |
| Alzheimer's Disease | ebi-a-GCST90027158 | 2022 | 85934 | 39106 | 46828 | EBI | European | 35379992 |
| Parkinson's disease | ebi-a-GCST90018894 | 2021 | 480018 | 2638 | 477380 | EBI | European | 34594039 |
| Myasthenia Gravis | ebi-a-GCST90093061 | 2022 | 38243 | 1873 | 36370 | EBI | European | 35074870 |
| Osteoarthritis | ebi-a-GCST90013881 | 2021 | 407746 | / | / | EBI | European | 34017140 |
| Osteoporosis | ebi-a-GCST90038656 | 2021 | 484598 | 7751 | 476847 | EBI | European | 33959723 |
| Total body bone mineral density (age 30–45) | ebi-a-GCST005346 | 2018 | 10062 | / | / | EBI | Mixed | 29304378 |
| Total body bone mineral density (age 45–60) | ebi-a-GCST005350 | 2018 | 18805 | / | / | EBI | European | 29304378 |
| Total body bone mineral density (age over 60) | ebi-a-GCST005349 | 2018 | 22504 | / | / | EBI | Mixed | 29304378 |
| Heel bone mineral density | ebi-a-GCST90025982 | 2021 | 445855 | / | / | EBI | European | 34226706 |
| Lumbar spine bone mineral density | ieu-a-982 | 2015 | 28498 | / | / | GEFOS | Mixed | 26367794 |
| Fracture of lower leg including ankle | finn-b-ST19_FRACT_LOWER_LEG_INCLU_ANKLE | 2021 | 201667 | 10489 | 191178 | FinnGen biobank | European | NA |
| Fracture of lumbar spine and pelvis | finn-b-ST19_FRACT_LUMBAR_SPINE_PELVIS | 2021 | 215698 | 2859 | 212839 | FinnGen biobank | European | NA |
| Intervertebral disk disorders | ukb-b-18279 | 2018 | 463010 | 2214 | 460796 | MRC-IEU | European | NA |
| Anxiety disorders | finn-b-KRA_PSY_ANXIETY | 2021 | 218792 | 20992 | 197800 | FinnGen biobank | European | NA |
| Bipolar disorder | ieu-b-5110 | 2021 | 413466 | 41917 | 371549 | PGC Bipolar Disorder Working Group of the Psychiatric Genomics Consortium | European | 34002096 |
| Depression | ebi-a-GCST90013878 | 2021 | 407746 | / | / | EBI | European | 34017140 |
| Schizophrenia | finn-b-KRA_PSY_SCHIZODEL | 2021 | 218792 | 10091 | 208701 | FinnGen biobank | European | NA |
| Malaise and fatigue | finn-b-R18_MALAI_FATIG | 2021 | 180362 | 7363 | 172999 | FinnGen biobank | European | NA |
| Urinary incontinence | finn-b-R18_UNSPE_URINARY_INCONTINENCE | 2021 | 204267 | 1357 | 202910 | FinnGen biobank | European | NA |
| Sleep apnea syndrome | ebi-a-GCST90018916 | 2021 | 476853 | 13818 | 463035 | EBI | European | 34594039 |
| Sleep disorders | finn-b-SLEEP | 2021 | 216700 | 19155 | 197545 | FinnGen biobank | European | NA |
| **Immunity, Infection, and Reproductive Disorders (IIRDs)** | | | | | | | | |
| Systemic lupus erythematosus | ebi-a-GCST90018917 | 2021 | 482911 | 647 | 482264 | EBI | European | 34594039 |
| Idiopathic thrombocytopenic purpura | ebi-a-GCST90018865 | 2021 | 489424 | 675 | 488749 | EBI | European | 34594039 |
| Rheumatoid arthritis | finn-b-RHEUMA_SEROPOS | 2021 | 177430 | 4596 | 172834 | FinnGen biobank | European | NA |
| Systemic sclerosis | finn-b-SYSTSCLE_STRICT | 2021 | 218606 | 107 | 218499 | FinnGen biobank | European | NA |
| Multiple sclerosis | finn-b-G6_MS | 2021 | 218189 | 1048 | 217141 | FinnGen biobank | European | NA |
| Urinary tract infection | ebi-a-GCST90013890 | 2021 | 397867 | / | / | EBI | European | 34017140 |
| Acute laryngitis and tracheitis | finn-b-J10_LARYNGITIS | 2021 | 185139 | 2194 | 182945 | FinnGen biobank | European | NA |
| Chronic laryngitis and laryngotracheitis | finn-b-J10_CHRONLARYNGITIS | 2021 | 169987 | 2138 | 167849 | FinnGen biobank | European | NA |
| Acute lower respiratory infections | finn-b-J10_LOWERINF | 2021 | 218792 | 10103 | 208689 | FinnGen biobank | European | NA |
| Acute upper respiratory infections | finn-b-J10_UPPERINFEC | 2021 | 218792 | 35847 | 182945 | FinnGen biobank | European | NA |
| Infections of the skin and subcutaneous tissue | finn-b-L12_INFECT_SKIN | 2021 | 218792 | 10343 | 208449 | FinnGen biobank | European | NA |
| Sepsis | ieu-b-4980 | 2021 | 486484 | 11643 | 474841 | UK Biobank | European | NA |
| Puerperal sepsis | finn-b-O15_PUERP_SEPSIS | 2021 | 121441 | 2286 | 119155 | FinnGen biobank | European | NA |
| Prostatitis | finn-b-N14_PROSTATITIS | 2021 | 74658 | 1859 | 72799 | FinnGen biobank | European | NA |
| Female infertility | finn-b-N14_FEMALEINFERT | 2021 | 75450 | 6481 | 68969 | FinnGen biobank | European | NA |
| Male infertility | finn-b-N14_MALEINFERT | 2021 | 73479 | 680 | 72799 | FinnGen biobank | European | NA |
| Sporadic miscarriage | ebi-a-GCST011888 | 2020 | 224105 | 49996 | 174109 | EBI | European | 33239672 |
| Ectopic pregnancy | finn-b-O15_PREG_ECTOP | 2021 | 92451 | 3111 | 89340 | FinnGen biobank | European | NA |
| Erectile dysfunction | ebi-a-GCST006956 | 2018 | 223805 | 6175 | 217630 | EBI | European | 30583798 |
| Sex hormone binding globulin | ebi-a-GCST90014011 | 2021 | 354620 | / | / | EBI | European | 34017140 |
| Polycystic ovary syndrome | ebi-a-GCST90044902 | 2021 | 141355 | 797 | 140558 | EBI | European | 34791234 |
| Preeclampsia | ebi-a-GCST90018906 | 2021 | 267242 | 2355 | 264887 | EBI | European | 34594039 |
| **Respiratory, Digestive, and Other Disorders (RDODs)** | | | | | | | | |
| Asthma | ebi-a-GCST90013888 | 2021 | 401847 | / | / | EBI | European | 34017140 |
| Chronic obstructive pulmonary disease | ebi-a-GCST90018807 | 2021 | 468475 | 13530 | 454945 | EBI | European | 34594039 |
| Pulmonary fibrosis | ebi-a-GCST90018908 | 2021 | 469126 | 1566 | 467560 | EBI | European | 34594039 |
| Acute pancreatitis | ebi-a-GCST90018789 | 2021 | 479902 | 3798 | 476104 | EBI | European | 34594039 |
| Cholecystitis | finn-b-CHOLELITH_BROAD | 2021 | 215027 | 19883 | 195144 | FinnGen biobank | European | NA |
| Cholelithiasis | finn-b-K11_CHOLELITH | 2021 | 214167 | 19023 | 195144 | FinnGen biobank | European | NA |
| Crohn's disease | ebi-a-GCST004132 | 2017 | 40266 | 12194 | 28072 | EBI | Mixed | 28067908 |
| Ulcerative colitis | finn-b-K11_ULCER | 2021 | 214620 | 4320 | 210300 | FinnGen biobank | European | NA |
| Gastroesophageal reflux disease | ebi-a-GCST90000514 | 2021 | 602604 | 129080 | 473524 | EBI | European | 34187846 |
| Irritable bowel syndrome | ebi-a-GCST90016564 | 2021 | 486601 | 53400 | 433201 | EBI | European | 34741163 |
| Barrett's esophagus | ebi-a-GCST003740 | 2016 | 10279 | 6167 | 4112 | EBI | European | 27527254 |
| Nonalcoholic fatty liver disease | finn-b-NAFLD | 2021 | 218792 | 894 | 217898 | FinnGen biobank | European | NA |
| Inguinal or femoral hernia bilateral | finn-b-FEMINGHER_BILATERAL | 2021 | 192104 | 1547 | 190557 | FinnGen biobank | European | NA |
| Acne | finn-b-L12_ACNE | 2021 | 212438 | 1299 | 211139 | FinnGen biobank | European | NA |
| Rosacea | finn-b-L12_ROSACEA | 2021 | 212334 | 1195 | 211139 | FinnGen biobank | European | NA |
| Residual haemorrhoidal skin tags | ukb-b-16882 | 2018 | 463010 | 1309 | 461701 | MRC-IEU | European | NA |
| Breast cancer | ebi-a-GCST90018799 | 2021 | 257730 | 17389 | 240341 | EBI | European | 34594039 |
| Lung adenocarcinoma | ebi-a-GCST004744 | 2017 | 66756 | 11273 | 55483 | EBI | European | 28604730 |
| Colon cancer | ukb-b-20145 | 2018 | 462933 | 1494 | 461439 | MRC-IEU | European | NA |
| Colon adenocarcinoma | finn-b-C3_COLON_ADENO_EXALLC | 2021 | 175402 | 1396 | 174006 | FinnGen biobank | European | NA |
| Endometrial cancer | ebi-a-GCST90018838 | 2021 | 240027 | 2188 | 237839 | EBI | European | 34594039 |
| Esophageal cancer | ebi-a-GCST90018841 | 2021 | 476306 | 998 | 475308 | EBI | European | 34594039 |
| Malignant neoplasm of kidney | finn-b-C3_KIDNEY_NOTRENALPELVIS | 2021 | 218792 | 971 | 217821 | FinnGen biobank | European | NA |
| Malignant neoplasm of prostate | finn-b-C3_PROSTATE_EXALLC | 2021 | 80996 | 6311 | 74685 | FinnGen biobank | European | NA |

Note: 92 diseases were classified into four major systems: Cardiovascular and Metabolic Disorders (CMDs), Immunity Infection and Reproductive Disorders (IIRDs), Neuromusculoskeletal and Mental Health Disorders (NMMDs), and Respiratory Digestive and Other Disorders (RDODs). Some outcomes appear as separate entities due to distinct phenotype definitions and data sources. For example, “Mild age-related type 2 diabetes” (from EBI) and “Type 2 diabetes” (from FinnGen biobank) represent different phenotype definitions; “Sleep apnea syndrome” (from EBI) and “Sleep disorders” (from FinnGen biobank) originate from independent consortia and could not be simply merged. Outcomes include both binary disease endpoints and continuous outcomes (e.g., serum uric acid, bone mineral density, fasting insulin, sex hormone binding globulin, and insulin receptor protein).

Abbreviations: UK, United Kingdom; MRC-IEU, medical research council integrative epidemiology Unit; EBI, European bioinformatics institute; PGC, psychiatric genomics consortium; GEFOS, genetic factors for osteoporosis consortium.
