# Supplementary material for: Unraveling the causal web of 4 adiposity indices and 92 multi-system outcomes: A body-wide Mendelian randomization study
Source: Medicine (Baltimore). 2026 May 22;105(21):e48986. doi: 10.1097/MD.0000000000048986 (PMC13201005; doi:10.1097/MD.0000000000048986)
Supplement: Supplementary file 3 [file medi-105-e48986-s003.docx]

Table S3. Profiles of adjustment factors for complementary multivariate Mendelian randomization analyses in genome-wide association studies datasets.

| **Exposure** | **GWAS ID (exposures)** | **Year** | **Sample size** | **Population** | **PMID** |
| --- | --- | --- | --- | --- | --- |
| Circulating leptin levels | ieu-a-1003 | 2016 | 32161 | European | 26833098 |
| MCP-1 | prot-c-2578_67_2 | 2019 | 1000 | European | 28240269 |
| Resistin levels | prot-a-2524 | 2018 | 3301 | European | 29875488 |
| C-reactive protein | ieu-b-35 | 2018 | 204402 | European | 30388399 |
| IL-6 | prot-c-4673_13_2 | 2019 | 1000 | European | 28240269 |
| TNF-α | prot-c-3722_49_2 | 2019 | 1000 | European | 28240269 |

Abbreviations: MCP-1, monocyte chemoattractant protein-1; IL-6, interleukin-6; TNF-α, tumor necrosis factor-alpha.
