# Supplementary material for: Unraveling the causal web of 4 adiposity indices and 92 multi-system outcomes: A body-wide Mendelian randomization study
Source: Medicine (Baltimore). 2026 May 22;105(21):e48986. doi: 10.1097/MD.0000000000048986 (PMC13201005; doi:10.1097/MD.0000000000048986)
Supplement: Supplementary file 4 [file medi-105-e48986-s004.docx]

Table S4. Potential confounders of different exposure-outcome pairs in two-sample Mendelian randomization analyses.

| **Exposures** | **GWAS ID (exposure)** | **Outcomes** | **GWAS ID (outcome)** | **Confounders** |
| --- | --- | --- | --- | --- |
| WC | ebi-a-GCST90014020 | Acne | finn-b-L12_ACNE | rosacea, anxiety, metabolic disorders, depression, diabetes, stress, smoking |
| HC | ebi-a-GCST90014021 | Acne | finn-b-L12_ACNE | rosacea, anxiety, metabolic disorders, depression, diabetes, stress, smoking |
| BMI | ebi-a-GCST90018947 | Acne | finn-b-L12_ACNE | rosacea, anxiety, metabolic disorders, depression, diabetes, stress, smoking |
| TFP | ukb-a-290 | Acne | finn-b-L12_ACNE | rosacea, anxiety, metabolic disorders, depression, diabetes, stress, smoking |
| WC | ebi-a-GCST90014020 | Acute laryngitis and tracheitis | finn-b-J10_LARYNGITIS | alcohol, diabetes, smoking |
| HC | ebi-a-GCST90014021 | Acute laryngitis and tracheitis | finn-b-J10_LARYNGITIS | alcohol, diabetes, smoking |
| BMI | ebi-a-GCST90018947 | Acute laryngitis and tracheitis | finn-b-J10_LARYNGITIS | alcohol, diabetes, smoking |
| TFP | ukb-a-290 | Acute laryngitis and tracheitis | finn-b-J10_LARYNGITIS | alcohol, diabetes, smoking |
| WC | ebi-a-GCST90014020 | Acute lower respiratory infections | finn-b-J10_LOWERINF | sleep apnea syndrome, bronchiectasis, chronic obstructive pulmonary disease, asthma, diabetes, stroke, heart failure, smoking, depression, malaise and fatigue |
| HC | ebi-a-GCST90014021 | Acute lower respiratory infections | finn-b-J10_LOWERINF | sleep apnea syndrome, bronchiectasis, chronic obstructive pulmonary disease, asthma, diabetes, stroke, heart failure, smoking, depression, malaise and fatigue |
| BMI | ebi-a-GCST90018947 | Acute lower respiratory infections | finn-b-J10_LOWERINF | sleep apnea syndrome, bronchiectasis, chronic obstructive pulmonary disease, asthma, diabetes, stroke, heart failure, smoking, depression, malaise and fatigue |
| TFP | ukb-a-290 | Acute lower respiratory infections | finn-b-J10_LOWERINF | sleep apnea syndrome, bronchiectasis, chronic obstructive pulmonary disease, asthma, diabetes, stroke, heart failure, smoking, depression, malaise and fatigue |
| BMI | ukb-a-248 | Acute pancreatitis | ebi-a-GCST90018789 | cholecystitis, cholelithiasis, biliary ascariasis, gallbladder polyps, smoking, alcohol intake frequency, alcoholic drinks per week, alcohol usually taken with meals, bile duct obstruction, diabetes, insulin resistance, pregnancy, liver cirrhosis, hyperthyroidism, hypothyroidism, C-reactive protein, hba1c, hyperglycemia, exercise, physical activity, educational attainment, income, anxiety disorders, depression, stress, endoscopic retrograde examination of bile duct and pancreatic duct, duodenal ulcer, duodenitis, familial combined hyperlipidemia, pancreatic ductal adenocarcinoma, pancreatic neuroendocrine tumors, intraductal papillary mucinous neoplasm, mucinous cystic neoplasm, solid pseudopapillary neoplasm, acinar cell carcinoma, ampullary cancer, cholangiocarcinoma, bile duct cancer, duodenal cancer, gastric cancer, hepatocellular carcinoma, systemic lupus erythematosus, inflammatory bowel disease, celiac disease, primary sclerosing cholangitis, vasculitis, familial chylomicronemia syndrome, familial partial lipodystrophy, apolipoprotein C-ii deficiency, familial combined hyperlipidemia, abetalipoproteinemia, tangier disease, cholestery ester storage disease, wolman disease, gaucher disease, fabry disease, niemann-pick disease, apolipoprotein a, apolipoprotein b, apolipoprotein C, apolipoprotein d, apolipoprotein e, apolipoprotein J, apolipoprotein m, ldl receptor, vldl receptor, Cd36, scavenger receptor class b type i, fatty acid transport proteins, fatty acid binding proteins, sterol carrier protein-2, steroidogenic acute regulatory protein, microsomal triglyceride transfer protein, atp-binding cassette transporters, igf-1, neutrophil cell count, gamma glutamyltransferase levels |
| TFP | ukb-a-290 | Acute pancreatitis | ebi-a-GCST90018789 | cholecystitis, cholelithiasis, biliary ascariasis, gallbladder polyps, smoking, alcohol intake frequency, alcoholic drinks per week, alcohol usually taken with meals |
| WC | ukb-a-382 | Acute pancreatitis | ebi-a-GCST90018789 | cholecystitis, cholelithiasis, biliary ascariasis, gallbladder polyps, smoking, alcohol intake frequency, alcoholic drinks per week, alcohol usually taken with meals |
| HC | ukb-a-388 | Acute pancreatitis | ebi-a-GCST90018789 | cholecystitis, cholelithiasis, biliary ascariasis, gallbladder polyps, smoking, alcohol intake frequency, alcoholic drinks per week, alcohol usually taken with meals |
| WC | ebi-a-GCST90014020 | Acute upper respiratory infections | finn-b-J10_UPPERINFEC | sleep apnea syndrome, bronchiectasis, chronic obstructive pulmonary disease, asthma, diabetes, stroke, heart failure, smoking, depression, malaise and fatigue |
| HC | ebi-a-GCST90014021 | Acute upper respiratory infections | finn-b-J10_UPPERINFEC | sleep apnea syndrome, bronchiectasis, chronic obstructive pulmonary disease, asthma, diabetes, stroke, heart failure, smoking, depression, malaise and fatigue |
| BMI | ebi-a-GCST90018947 | Acute upper respiratory infections | finn-b-J10_UPPERINFEC | sleep apnea syndrome, bronchiectasis, chronic obstructive pulmonary disease, asthma, diabetes, stroke, heart failure, smoking, depression, malaise and fatigue |
| TFP | ukb-a-290 | Acute upper respiratory infections | finn-b-J10_UPPERINFEC | sleep apnea syndrome, bronchiectasis, chronic obstructive pulmonary disease, asthma, diabetes, stroke, heart failure, smoking, depression, malaise and fatigue |
| BMI | ukb-a-248 | Alzheimer’s disease | ebi-a-GCST90027158 | hypertension, diabetes, gout, sleep apnea syndrome, kidney disease, heart failure, atrial fibrillation, left ventricular hypertrophy, smoking, depression |
| TFP | ukb-a-290 | Alzheimer’s disease | ebi-a-GCST90027158 | hypertension, diabetes, gout, sleep apnea syndrome, kidney disease, heart failure, atrial fibrillation, left ventricular hypertrophy, smoking, depression |
| WC | ukb-a-382 | Alzheimer’s disease | ebi-a-GCST90027158 | hypertension, diabetes, gout, sleep apnea syndrome, kidney disease, heart failure, atrial fibrillation, left ventricular hypertrophy, smoking, depression |
| HC | ukb-a-388 | Alzheimer’s disease | ebi-a-GCST90027158 | hypertension, diabetes, gout, sleep apnea syndrome, kidney disease, heart failure, atrial fibrillation, left ventricular hypertrophy, smoking, depression |
| WC | ebi-a-GCST90014020 | Anxiety disorders | finn-b-KRA_PSY_ANXIETY | malaise and fatigue, sleep disorders, urinary incontinence, depression, stress, infertility, hypertension, heart failure, diabetes, stroke, coronary atherosclerosis |
| HC | ebi-a-GCST90014021 | Anxiety disorders | finn-b-KRA_PSY_ANXIETY | malaise and fatigue, sleep disorders, urinary incontinence, depression, stress, infertility, hypertension, heart failure, diabetes, stroke, coronary atherosclerosis |
| BMI | ebi-a-GCST90018947 | Anxiety disorders | finn-b-KRA_PSY_ANXIETY | malaise and fatigue, sleep disorders, urinary incontinence, depression, stress, infertility, hypertension, heart failure, diabetes, stroke, coronary atherosclerosis |
| TFP | ukb-a-290 | Anxiety disorders | finn-b-KRA_PSY_ANXIETY | malaise and fatigue, sleep disorders, urinary incontinence, depression, stress, infertility, hypertension, heart failure, diabetes, stroke, coronary atherosclerosis |
| BMI | ukb-a-248 | Asthma | ebi-a-GCST90013888 | acute lower respiratory infections, sleep apnea syndrome, chronic obstructive pulmonary disease, diabetes, heart failure, smoking |
| TFP | ukb-a-290 | Asthma | ebi-a-GCST90013888 | acute lower respiratory infections, sleep apnea syndrome, chronic obstructive pulmonary disease, diabetes, heart failure, smoking |
| WC | ukb-a-382 | Asthma | ebi-a-GCST90013888 | acute lower respiratory infections, sleep apnea syndrome, chronic obstructive pulmonary disease, diabetes, heart failure, smoking |
| HC | ukb-a-388 | Asthma | ebi-a-GCST90013888 | acute lower respiratory infections, sleep apnea syndrome, chronic obstructive pulmonary disease, diabetes, heart failure, smoking |
| WC | ebi-a-GCST90014020 | Atrial fibrillation and flutter | finn-b-I9_AF | gout, hypertension, coronary heart disease, heart failure, diabetes, sleep apnea, kidney disease, left ventricular hypertrophy, smoking |
| HC | ebi-a-GCST90014021 | Atrial fibrillation and flutter | finn-b-I9_AF | gout, hypertension, coronary heart disease, heart failure, diabetes, sleep apnea, kidney disease, left ventricular hypertrophy, smoking |
| BMI | ebi-a-GCST90018947 | Atrial fibrillation and flutter | finn-b-I9_AF | gout, hypertension, coronary heart disease, heart failure, diabetes, sleep apnea, kidney disease, left ventricular hypertrophy, smoking |
| TFP | ukb-a-290 | Atrial fibrillation and flutter | finn-b-I9_AF | gout, hypertension, coronary heart disease, heart failure, diabetes, sleep apnea, kidney disease, left ventricular hypertrophy, smoking |
| BMI | ukb-a-248 | Barrett's esophagus | ebi-a-GCST003740 | alcohol, diabetes, smoking |
| TFP | ukb-a-290 | Barrett's esophagus | ebi-a-GCST003740 | alcohol, diabetes, smoking |
| WC | ukb-a-382 | Barrett’s esophagus | ebi-a-GCST003740 | alcohol, diabetes, smoking |
| HC | ukb-a-388 | Barrett’s esophagus | ebi-a-GCST003740 | alcohol, diabetes, smoking |
| WC | ebi-a-GCST90014020 | Bipolar disorder | ieu-b-5110 | malaise and fatigue, sleep disorders, urinary incontinence, depression, stress, infertility, hypertension, heart failure, diabetes, stroke, coronary atherosclerosis |
| HC | ebi-a-GCST90014021 | Bipolar disorder | ieu-b-5110 | malaise and fatigue, sleep disorders, urinary incontinence, depression, stress, infertility, hypertension, heart failure, diabetes, stroke, coronary atherosclerosis |
| BMI | ebi-a-GCST90018947 | Bipolar disorder | ieu-b-5110 | malaise and fatigue, sleep disorders, urinary incontinence, depression, stress, infertility, hypertension, heart failure, diabetes, stroke, coronary atherosclerosis |
| TFP | ukb-a-290 | Bipolar disorder | ieu-b-5110 | malaise and fatigue, sleep disorders, urinary incontinence, depression, stress, infertility, hypertension, heart failure, diabetes, stroke, coronary atherosclerosis |
| BMI | ukb-a-248 | Breast cancer | ebi-a-GCST90018799 | alcohol intake frequency, alcoholic drinks per week, alcohol usually taken with meals, diabetes, polycystic ovary syndrome, insulin resistance, smoking |
| TFP | ukb-a-290 | Breast cancer | ebi-a-GCST90018799 | alcohol intake frequency, alcoholic drinks per week, alcohol usually taken with meals, diabetes, polycystic ovary syndrome, insulin resistance, smoking |
| WC | ukb-a-382 | Breast cancer | ebi-a-GCST90018799 | alcohol intake frequency, alcoholic drinks per week, alcohol usually taken with meals, diabetes, polycystic ovary syndrome, insulin resistance, smoking |
| HC | ukb-a-388 | Breast cancer | ebi-a-GCST90018799 | alcohol intake frequency, alcoholic drinks per week, alcohol usually taken with meals, diabetes, polycystic ovary syndrome, insulin resistance, smoking |
| WC | ebi-a-GCST90014020 | Cerebral atherosclerosis | finn-b-I9_CERATHER | hypertension, gout, diabetes, sleep apnea, kidney disease, smoking |
| HC | ebi-a-GCST90014021 | Cerebral atherosclerosis | finn-b-I9_CERATHER | hypertension, gout, diabetes, sleep apnea, kidney disease, smoking |
| BMI | ebi-a-GCST90018947 | Cerebral atherosclerosis | finn-b-I9_CERATHER | hypertension, gout, diabetes, sleep apnea, kidney disease, smoking |
| TFP | ukb-a-290 | Cerebral atherosclerosis | finn-b-I9_CERATHER | hypertension, gout, diabetes, sleep apnea, kidney disease, smoking |
| WC | ebi-a-GCST90014020 | Cholecystitis | finn-b-CHOLELITH_BROAD | biliary ascariasis, gallbladder polyps, bile duct obstruction, diabetes, pregnancy, liver cirrhosis |
| HC | ebi-a-GCST90014021 | Cholecystitis | finn-b-CHOLELITH_BROAD | biliary ascariasis, gallbladder polyps, bile duct obstruction, diabetes, pregnancy, liver cirrhosis, hyperthyroidism, alcohol intake frequency, alcoholic drinks per week, alcohol usually taken with meals, anxiety disorders, depression, stress, C-reactive protein, hba1c, hyperglycemia, exercise, physical activity, educational attainment, income |
| BMI | ebi-a-GCST90018947 | Cholecystitis | finn-b-CHOLELITH_BROAD | biliary ascariasis, gallbladder polyps, bile duct obstruction, diabetes, pregnancy, liver cirrhosis |
| TFP | ukb-a-290 | Cholecystitis | finn-b-CHOLELITH_BROAD | biliary ascariasis, gallbladder polyps, bile duct obstruction, diabetes, pregnancy, liver cirrhosis |
| WC | ebi-a-GCST90014020 | Cholelithiasis | finn-b-K11_CHOLELITH | biliary ascariasis, gallbladder polyps, bile duct obstruction, diabetes, pregnancy, liver cirrhosis |
| HC | ebi-a-GCST90014021 | Cholelithiasis | finn-b-K11_CHOLELITH | biliary ascariasis, gallbladder polyps, bile duct obstruction, diabetes, pregnancy, liver cirrhosis, hyperthyroidism, alcohol intake frequency, alcoholic drinks per week, alcohol usually taken with meals, anxiety disorders, depression, stress, C-reactive protein, hba1c, hyperglycemia, exercise, physical activity, educational attainment, income |
| BMI | ebi-a-GCST90018947 | Cholelithiasis | finn-b-K11_CHOLELITH | biliary ascariasis, gallbladder polyps, bile duct obstruction, diabetes, pregnancy, liver cirrhosis |
| TFP | ukb-a-290 | Cholelithiasis | finn-b-K11_CHOLELITH | biliary ascariasis, gallbladder polyps, bile duct obstruction, diabetes, pregnancy, liver cirrhosis |
| WC | ebi-a-GCST90014020 | Chronic laryngitis and laryngotracheitis | finn-b-J10_CHRONLARYNGITIS | alcohol, diabetes, smoking |
| HC | ebi-a-GCST90014021 | Chronic laryngitis and laryngotracheitis | finn-b-J10_CHRONLARYNGITIS | alcohol, diabetes, smoking |
| BMI | ebi-a-GCST90018947 | Chronic laryngitis and laryngotracheitis | finn-b-J10_CHRONLARYNGITIS | alcohol, diabetes, smoking |
| TFP | ukb-a-290 | Chronic laryngitis and laryngotracheitis | finn-b-J10_CHRONLARYNGITIS | alcohol, diabetes, smoking |
| BMI | ukb-a-248 | Chronic obstructive pulmonary disease | ebi-a-GCST90018807 | acute lower respiratory infections, sleep apnea syndrome, asthma, diabetes, heart failure, smoking |
| TFP | ukb-a-290 | Chronic obstructive pulmonary disease | ebi-a-GCST90018807 | acute lower respiratory infections, sleep apnea syndrome, asthma, diabetes, heart failure, smoking, silicosis, asbestosis, Coal worker's pneumoconiosis, rheumatoid arthritis, alcohol intake frequency, alcoholic drinks per week, alcohol usually taken with meals, anxiety disorders, depression, stress, C-reactive protein, hba1c, hyperglycemia, exercise, physical activity, educational attainment, income |
| WC | ukb-a-382 | Chronic obstructive pulmonary disease | ebi-a-GCST90018807 | acute lower respiratory infections, sleep apnea syndrome, asthma, diabetes, heart failure, smoking |
| HC | ukb-a-388 | Chronic obstructive pulmonary disease | ebi-a-GCST90018807 | acute lower respiratory infections, sleep apnea syndrome, asthma, diabetes, heart failure, smoking |
| WC | ebi-a-GCST90014020 | Colon adenocarcinoma | finn-b-C3_COLON_ADENO_EXALLC | alcohol, diabetes, smoking |
| HC | ebi-a-GCST90014021 | Colon adenocarcinoma | finn-b-C3_COLON_ADENO_EXALLC | alcohol, diabetes, smoking |
| BMI | ebi-a-GCST90018947 | Colon adenocarcinoma | finn-b-C3_COLON_ADENO_EXALLC | alcohol, diabetes, smoking |
| TFP | ukb-a-290 | Colon adenocarcinoma | finn-b-C3_COLON_ADENO_EXALLC | alcohol, diabetes, smoking |
| BMI | ukb-a-248 | Colon cancer | ukb-b-20145 | alcohol intake frequency, diabetes, polycystic ovary syndrome, insulin resistance, smoking, gout, alcohol intake frequency, alcoholic drinks per week, alcohol usually taken with meals, anxiety disorders, depression, stress, C-reactive protein, hba1c, hyperglycemia, exercise, physical activity, educational attainment, income |
| TFP | ukb-a-290 | Colon cancer | ukb-b-20145 | alcohol intake frequency, diabetes, polycystic ovary syndrome, insulin resistance, smoking, gout |
| WC | ukb-a-382 | Colon cancer | ukb-b-20145 | alcohol intake frequency, diabetes, polycystic ovary syndrome, insulin resistance, smoking, gout |
| HC | ukb-a-388 | Colon cancer | ukb-b-20145 | alcohol intake frequency, diabetes, polycystic ovary syndrome, insulin resistance, smoking, gout |
| WC | ebi-a-GCST90014020 | Coronary atherosclerosis | finn-b-I9_CORATHER | hypertension, gout, heart failure, diabetes, sleep apnea, kidney disease, smoking |
| HC | ebi-a-GCST90014021 | Coronary atherosclerosis | finn-b-I9_CORATHER | hypertension, gout, heart failure, diabetes, sleep apnea, kidney disease, smoking |
| BMI | ebi-a-GCST90018947 | Coronary atherosclerosis | finn-b-I9_CORATHER | hypertension, gout, heart failure, diabetes, sleep apnea, kidney disease, smoking |
| TFP | ukb-a-290 | Coronary atherosclerosis | finn-b-I9_CORATHER | hypertension, gout, heart failure, diabetes, sleep apnea, kidney disease, smoking |
| WC | ebi-a-GCST90014020 | Coronary heart disease | finn-b-I9_CHD | hypertension, gout, heart failure, diabetes, sleep apnea, kidney disease, smoking |
| HC | ebi-a-GCST90014021 | Coronary heart disease | finn-b-I9_CHD | hypertension, gout, heart failure, diabetes, sleep apnea, kidney disease, smoking |
| BMI | ebi-a-GCST90018947 | Coronary heart disease | finn-b-I9_CHD | hypertension, gout, heart failure, diabetes, sleep apnea, kidney disease, smoking |
| TFP | ukb-a-290 | Coronary heart disease | finn-b-I9_CHD | hypertension, gout, heart failure, diabetes, sleep apnea, kidney disease, smoking |
| BMI | ukb-a-248 | Crohn's disease | ebi-a-GCST004132 | thymoma, psoriasis, addiction |
| TFP | ukb-a-290 | Crohn's disease | ebi-a-GCST004132 | thymoma, psoriasis, addiction |
| WC | ukb-a-382 | Crohn's disease | ebi-a-GCST004132 | thymoma, psoriasis, addiction |
| HC | ukb-a-388 | Crohn's disease | ebi-a-GCST004132 | thymoma, psoriasis, addiction |
| BMI | ukb-a-248 | Depression | ebi-a-GCST90013878 | malaise and fatigue, sleep disorders, urinary incontinence, stress, infertility, hypertension, heart failure, diabetes, stroke |
| TFP | ukb-a-290 | Depression | ebi-a-GCST90013878 | malaise and fatigue, sleep disorders, urinary incontinence, stress, infertility, hypertension, heart failure, diabetes, stroke |
| WC | ukb-a-382 | Depression | ebi-a-GCST90013878 | malaise and fatigue, sleep disorders, urinary incontinence, stress, infertility, hypertension, heart failure, diabetes, stroke, alcohol intake frequency, alcoholic drinks per week, alcohol usually taken with meals, anxiety disorders, C-reactive protein, hba1c, hyperglycemia, exercise, physical activity, educational attainment, income, chronic obstructive pulmonary disease, asthma |
| HC | ukb-a-388 | Depression | ebi-a-GCST90013878 | malaise and fatigue, sleep disorders, urinary incontinence, stress, infertility, hypertension, heart failure, diabetes, stroke |
| WC | ebi-a-GCST90014020 | Ectopic pregnancy | finn-b-O15_PREG_ECTOP | puerperal sepsis, pelvic inflammatory, tubal surgery, smoking, diabetes |
| HC | ebi-a-GCST90014021 | Ectopic pregnancy | finn-b-O15_PREG_ECTOP | puerperal sepsis, pelvic inflammatory, tubal surgery, smoking, diabetes |
| BMI | ebi-a-GCST90018947 | Ectopic pregnancy | finn-b-O15_PREG_ECTOP | puerperal sepsis, pelvic inflammatory, tubal surgery, smoking, diabetes |
| TFP | ukb-a-290 | Ectopic pregnancy | finn-b-O15_PREG_ECTOP | puerperal sepsis, pelvic inflammatory, tubal surgery, smoking, diabetes |
| BMI | ukb-a-248 | Endometrial cancer | ebi-a-GCST90018838 | endometrial hyperplasia, ectopic pregnancy, infertility, puerperal sepsis, pelvic inflammatory, tubal surgery, smoking, diabetes |
| TFP | ukb-a-290 | Endometrial cancer | ebi-a-GCST90018838 | endometrial hyperplasia, ectopic pregnancy, infertility, puerperal sepsis, pelvic inflammatory, tubal surgery, smoking, diabetes |
| WC | ukb-a-382 | Endometrial cancer | ebi-a-GCST90018838 | endometrial hyperplasia, ectopic pregnancy, infertility, puerperal sepsis, pelvic inflammatory, tubal surgery, smoking, diabetes |
| HC | ukb-a-388 | Endometrial cancer | ebi-a-GCST90018838 | endometrial hyperplasia, ectopic pregnancy, infertility, puerperal sepsis, pelvic inflammatory, tubal surgery, smoking, diabetes |
| BMI | ukb-a-248 | Erectile dysfunction | ebi-a-GCST006956 | thymoma, psoriasis, addiction |
| TFP | ukb-a-290 | Erectile dysfunction | ebi-a-GCST006956 | thymoma, psoriasis, addiction |
| WC | ukb-a-382 | Erectile dysfunction | ebi-a-GCST006956 | thymoma, psoriasis, addiction |
| HC | ukb-a-388 | Erectile dysfunction | ebi-a-GCST006956 | thymoma, psoriasis, addiction |
| BMI | ukb-a-248 | Esophageal cancer | ebi-a-GCST90018841 | alcohol intake frequency, alcoholic drinks per week, alcohol usually taken with meals, diabetes, polycystic ovary syndrome, smoking |
| TFP | ukb-a-290 | Esophageal cancer | ebi-a-GCST90018841 | alcohol intake frequency, alcoholic drinks per week, alcohol usually taken with meals, diabetes, polycystic ovary syndrome, smoking |
| WC | ukb-a-382 | Esophageal cancer | ebi-a-GCST90018841 | alcohol intake frequency, alcoholic drinks per week, alcohol usually taken with meals, diabetes, polycystic ovary syndrome, smoking |
| HC | ukb-a-388 | Esophageal cancer | ebi-a-GCST90018841 | alcohol intake frequency, alcoholic drinks per week, alcohol usually taken with meals, diabetes, polycystic ovary syndrome, smoking |
| BMI | ukb-a-248 | Familial combined hyperlipidemia | ebi-a-GCST90104003 | alcohol, diabetes, cancer |
| TFP | ukb-a-290 | Familial combined hyperlipidemia | ebi-a-GCST90104003 | alcohol, diabetes, cancer |
| WC | ukb-a-382 | Familial combined hyperlipidemia | ebi-a-GCST90104003 | alcohol, diabetes, cancer |
| HC | ukb-a-388 | Familial combined hyperlipidemia | ebi-a-GCST90104003 | alcohol, diabetes, cancer |
| BMI | ukb-a-248 | Fasting insulin | ebi-a-GCST90002238 | gout, hyperthyroidism, hypothyroidism, pancreatitis, atherosclerosis, heart failure, sleep apnea, smoking, sleep disorders, anxiety, depression, stroke, hyperthyroidism, alcohol intake frequency, alcoholic drinks per week, alcohol usually taken with meals, stress, C-reactive protein, hba1c, hyperglycemia, exercise, physical activity, educational attainment, income |
| TFP | ukb-a-290 | Fasting insulin | ebi-a-GCST90002238 | gout, hyperthyroidism, hypothyroidism, pancreatitis, coronary heart disease, heart failure, sleep apnea, smoking, sleep disorders, anxiety, depression, stroke |
| WC | ukb-a-382 | Fasting insulin | ebi-a-GCST90002238 | gout, hyperthyroidism, hypothyroidism, pancreatitis, atherosclerosis, heart failure, sleep apnea, smoking, sleep disorders, anxiety, depression, stroke, hyperthyroidism, alcohol intake frequency, alcoholic drinks per week, alcohol usually taken with meals, stress, C-reactive protein, hba1c, hyperglycemia, exercise, physical activity, educational attainment, income |
| HC | ukb-a-388 | Fasting insulin | ebi-a-GCST90002238 | gout, hyperthyroidism, hypothyroidism, pancreatitis, coronary heart disease, heart failure, sleep apnea, smoking, sleep disorders, anxiety, depression, stroke |
| WC | ebi-a-GCST90014020 | Female infertility | finn-b-N14_FEMALEINFERT | ectopic pregnancy, puerperal sepsis, pelvic inflammatory, tubal surgery, smoking, diabetes, anxiety, depression, stress, thymoma, psoriasis, addiction |
| HC | ebi-a-GCST90014021 | Female infertility | finn-b-N14_FEMALEINFERT | ectopic pregnancy, puerperal sepsis, pelvic inflammatory, tubal surgery, smoking, diabetes, anxiety, depression, stress, thymoma, psoriasis, addiction |
| BMI | ebi-a-GCST90018947 | Female infertility | finn-b-N14_FEMALEINFERT | ectopic pregnancy, puerperal sepsis, pelvic inflammatory, tubal surgery, smoking, diabetes, anxiety, depression, stress, thymoma, psoriasis, addiction, insulin resistance, hyperandrogenism, estrogen, irregular menstruation, anovulation, amenorrhea, sleep disorders, anxiety, depression, stress, diabetes, cardiovascular disease, hypertension, kidney disease |
| TFP | ukb-a-290 | Female infertility | finn-b-N14_FEMALEINFERT | ectopic pregnancy, puerperal sepsis, pelvic inflammatory, tubal surgery, smoking, diabetes, anxiety, depression, stress, thymoma, psoriasis, addiction, insulin resistance, hyperandrogenism, estrogen, irregular menstruation, anovulation, amenorrhea, sleep disorders, anxiety, depression, stress, diabetes, cardiovascular disease, hypertension, kidney disease |
| WC | ebi-a-GCST90014020 | Fracture of lower leg, including ankle | finn-b-ST19_FRACT_LOWER_LEG_INCLU_ANKLE | osteoporosis, osteoarthritis, hypocalcemia, calcium deficiency, vitamin d deficiency |
| HC | ebi-a-GCST90014021 | Fracture of lower leg, including ankle | finn-b-ST19_FRACT_LOWER_LEG_INCLU_ANKLE | osteoporosis, osteoarthritis, hypocalcemia, calcium deficiency, vitamin d deficiency |
| BMI | ebi-a-GCST90018947 | Fracture of lower leg, including ankle | finn-b-ST19_FRACT_LOWER_LEG_INCLU_ANKLE | osteoporosis, osteoarthritis, hypocalcemia, calcium deficiency, vitamin d deficiency |
| TFP | ukb-a-290 | Fracture of lower leg, including ankle | finn-b-ST19_FRACT_LOWER_LEG_INCLU_ANKLE | osteoporosis, osteoarthritis, hypocalcemia, calcium deficiency, vitamin d deficiency |
| WC | ebi-a-GCST90014020 | Fracture of lumbar spine and pelvis | finn-b-ST22_FRACT_LUMBAR_SPINE_PELVIS | osteoporosis, osteoarthritis, hypocalcemia, calcium deficiency, vitamin d deficiency |
| HC | ebi-a-GCST90014021 | Fracture of lumbar spine and pelvis | finn-b-ST20_FRACT_LUMBAR_SPINE_PELVIS | osteoporosis, osteoarthritis, hypocalcemia, calcium deficiency, vitamin d deficiency |
| BMI | ebi-a-GCST90018947 | Fracture of lumbar spine and pelvis | finn-b-ST19_FRACT_LUMBAR_SPINE_PELVIS | osteoporosis, osteoarthritis, hypocalcemia, calcium deficiency, vitamin d deficiency |
| TFP | ukb-a-290 | Fracture of lumbar spine and pelvis | finn-b-ST21_FRACT_LUMBAR_SPINE_PELVIS | osteoporosis, osteoarthritis, hypocalcemia, calcium deficiency, vitamin d deficiency |
| BMI | ukb-a-248 | Gastroesophageal reflux disease | ebi-a-GCST90000514 | alcohol intake frequency, alcoholic drinks per week, alcohol usually taken with meals, hiatal hernia, smoking, anxiety disorders, bipolar disorder, malaise and fatigue, sleep disorders, depression, stress, pregnancy, connective tissue disorders |
| TFP | ukb-a-290 | Gastroesophageal reflux disease | ebi-a-GCST90000514 | alcohol intake frequency, alcoholic drinks per week, alcohol usually taken with meals, hiatal hernia, smoking, anxiety disorders, bipolar disorder, malaise and fatigue, sleep disorders, depression, stress, pregnancy, connective tissue disorders |
| WC | ukb-a-382 | Gastroesophageal reflux disease | ebi-a-GCST90000514 | alcohol intake frequency, alcoholic drinks per week, alcohol usually taken with meals, hiatal hernia, smoking, anxiety disorders, bipolar disorder, malaise and fatigue, sleep disorders, depression, stress, pregnancy, connective tissue disorders |
| HC | ukb-a-388 | Gastroesophageal reflux disease | ebi-a-GCST90000514 | alcohol intake frequency, alcoholic drinks per week, alcohol usually taken with meals, hiatal hernia, smoking, anxiety disorders, bipolar disorder, malaise and fatigue, sleep disorders, depression, stress, pregnancy, connective tissue disorders |
| WC | ebi-a-GCST90014020 | Gestational diabetes | finn-b-GEST_DIABETES | gout, hyperthyroidism, hypothyroidism, pancreatitis, atherosclerosis, heart failure, sleep apnea, smoking, sleep disorders, anxiety, depression, stroke |
| HC | ebi-a-GCST90014021 | Gestational diabetes | finn-b-GEST_DIABETES | gout, hyperthyroidism, hypothyroidism, pancreatitis, atherosclerosis, heart failure, sleep apnea, smoking, sleep disorders, anxiety, depression, stroke |
| BMI | ebi-a-GCST90018947 | Gestational diabetes | finn-b-GEST_DIABETES | gout, hyperthyroidism, hypothyroidism, pancreatitis, atherosclerosis, heart failure, sleep apnea, smoking, sleep disorders, anxiety, depression, stroke |
| TFP | ukb-a-290 | Gestational diabetes | finn-b-GEST_DIABETES | gout, hyperthyroidism, hypothyroidism, pancreatitis, atherosclerosis, heart failure, sleep apnea, smoking, sleep disorders, anxiety, depression, stroke |
| BMI | ukb-a-248 | Gout | ebi-a-GCST90038687 | alcohol intake frequency, alcoholic drinks per week, alcohol usually taken with meals, smoking, kidney disease, diabetes |
| TFP | ukb-a-290 | Gout | ebi-a-GCST90038687 | alcohol intake frequency, alcoholic drinks per week, alcohol usually taken with meals, smoking, kidney disease, diabetes |
| WC | ukb-a-382 | Gout | ebi-a-GCST90038687 | alcohol intake frequency, alcoholic drinks per week, alcohol usually taken with meals, smoking, kidney disease, diabetes |
| HC | ukb-a-388 | Gout | ebi-a-GCST90038687 | alcohol intake frequency, alcoholic drinks per week, alcohol usually taken with meals, smoking, kidney disease, diabetes |
| WC | ebi-a-GCST90014020 | Heart failure | finn-b-I9_HEARTFAIL | atrial fibrillation and flutter, gout, hypertension, coronary heart disease, diabetes, sleep apnea, kidney disease, left ventricular hypertrophy, cardiomyopathy, smoking |
| HC | ebi-a-GCST90014021 | Heart failure | finn-b-I9_HEARTFAIL | atrial fibrillation and flutter, gout, hypertension, coronary heart disease, diabetes, sleep apnea, kidney disease, left ventricular hypertrophy, cardiomyopathy, smoking |
| BMI | ebi-a-GCST90018947 | Heart failure | finn-b-I9_HEARTFAIL | atrial fibrillation and flutter, gout, hypertension, coronary heart disease, diabetes, sleep apnea, kidney disease, left ventricular hypertrophy, cardiomyopathy, smoking |
| TFP | ukb-a-290 | Heart failure | finn-b-I9_HEARTFAIL | atrial fibrillation and flutter, gout, hypertension, coronary heart disease, diabetes, sleep apnea, kidney disease, left ventricular hypertrophy, cardiomyopathy, smoking |
| BMI | ukb-a-248 | Heel bone mineral density | ebi-a-GCST90025982 | calcium deficiency, vitamin d deficiency, malnutrition |
| TFP | ukb-a-290 | Heel bone mineral density | ebi-a-GCST90025982 | calcium deficiency, vitamin d deficiency, malnutrition |
| WC | ukb-a-382 | Heel bone mineral density | ebi-a-GCST90025982 | calcium deficiency, vitamin d deficiency, malnutrition |
| HC | ukb-a-388 | Heel bone mineral density | ebi-a-GCST90025982 | calcium deficiency, vitamin d deficiency, malnutrition |
| WC | ebi-a-GCST90014020 | Hypertension | finn-b-I9_HYPTENSESS | renal artery stenosis, hyperaldosteronism, aldosteronism, Cushing's syndrome, pheochromocytoma, gout, diabetes, sleep apnea, kidney disease, metabolic disorders, left ventricular hypertrophy, smoking, hyperthyroidism |
| HC | ebi-a-GCST90014021 | Hypertension | finn-b-I9_HYPTENSESS | renal artery stenosis, hyperaldosteronism, aldosteronism, Cushing's syndrome, pheochromocytoma, gout, diabetes, sleep apnea, kidney disease, metabolic disorders, left ventricular hypertrophy, smoking, hyperthyroidism , alcohol intake frequency, alcoholic drinks per week, alcohol usually taken with meals, anxiety disorders, depression, stress, C-reactive protein, hba1c, hyperglycemia, exercise, physical activity, educational attainment, income |
| BMI | ebi-a-GCST90018947 | Hypertension | finn-b-I9_HYPTENSESS | renal artery stenosis, hyperaldosteronism, aldosteronism, Cushing's syndrome, pheochromocytoma, gout, diabetes, sleep apnea, kidney disease, metabolic disorders, left ventricular hypertrophy, smoking, hyperthyroidism |
| TFP | ukb-a-290 | Hypertension | finn-b-I9_HYPTENSESS | renal artery stenosis, hyperaldosteronism, aldosteronism, Cushing's syndrome, pheochromocytoma, gout, diabetes, sleep apnea, kidney disease, metabolic disorders, left ventricular hypertrophy, smoking, hyperthyroidism |
| BMI | ukb-a-248 | Hyperthyroidism | ebi-a-GCST90018860 | pregnancy, smoking, kidney disease, diabetes, anxiety disorders, bipolar disorder, sleep disorders, depression, stress, amiodarone, thyroid cancer |
| TFP | ukb-a-290 | Hyperthyroidism | ebi-a-GCST90018860 | pregnancy, smoking, kidney disease, diabetes, anxiety disorders, bipolar disorder, sleep disorders, depression, stress, amiodarone, thyroid cancer |
| WC | ukb-a-382 | Hyperthyroidism | ebi-a-GCST90018860 | pregnancy, smoking, kidney disease, diabetes, anxiety disorders, bipolar disorder, sleep disorders, depression, stress, amiodarone, thyroid cancer, iodine, graves' disease, hashimoto's thyroiditis, radiation, pituitary, selenium, vitamin d, alcohol intake frequency, alcoholic drinks per week, alcohol usually taken with meals, stress, C-reactive protein, hba1c, hyperglycemia, exercise, physical activity, educational attainment, income |
| HC | ukb-a-388 | Hyperthyroidism | ebi-a-GCST90018860 | pregnancy, smoking, kidney disease, diabetes, anxiety disorders, bipolar disorder, sleep disorders, depression, stress, amiodarone, thyroid cancer, iodine, graves' disease, hashimoto's thyroiditis, radiation, pituitary, selenium, vitamin d, alcohol intake frequency, alcoholic drinks per week, alcohol usually taken with meals, stress, C-reactive protein, hba1c, hyperglycemia, exercise, physical activity, educational attainment, income |
| BMI | ukb-a-248 | Hypothyroidism | ebi-a-GCST90013893 | pregnancy, smoking, kidney disease, diabetes, anxiety disorders, bipolar disorder, sleep disorders, depression, stress, thyroid cancer |
| TFP | ukb-a-290 | Hypothyroidism | ebi-a-GCST90013893 | pregnancy, smoking, kidney disease, diabetes, anxiety disorders, bipolar disorder, sleep disorders, depression, stress, thyroid cancer |
| WC | ukb-a-382 | Hypothyroidism | ebi-a-GCST90013893 | pregnancy, smoking, kidney disease, diabetes, anxiety disorders, bipolar disorder, sleep disorders, depression, stress, thyroid cancer |
| HC | ukb-a-388 | Hypothyroidism | ebi-a-GCST90013893 | pregnancy, smoking, kidney disease, diabetes, anxiety disorders, bipolar disorder, sleep disorders, depression, stress, thyroid cancer |
| BMI | ukb-a-248 | Idiopathic thrombocytopenic purpura | ebi-a-GCST90018865 | thymoma, psoriasis, addiction |
| TFP | ukb-a-290 | Idiopathic thrombocytopenic purpura | ebi-a-GCST90018865 | thymoma, psoriasis, addiction |
| WC | ukb-a-382 | Idiopathic thrombocytopenic purpura | ebi-a-GCST90018865 | thymoma, psoriasis, addiction |
| HC | ukb-a-388 | Idiopathic thrombocytopenic purpura | ebi-a-GCST90018865 | thymoma, psoriasis, addiction |
| WC | ebi-a-GCST90014020 | infections of the Skin and subcutaneous tissue | finn-b-L12_INFECT_SKIN | chronic urticaria, eczema, acne, rosacea, anxiety, diabetes, stress, smoking |
| HC | ebi-a-GCST90014021 | infections of the Skin and subcutaneous tissue | finn-b-L12_INFECT_SKIN | chronic urticaria, eczema, acne, rosacea, anxiety, diabetes, stress, smoking |
| BMI | ebi-a-GCST90018947 | infections of the Skin and subcutaneous tissue | finn-b-L12_INFECT_SKIN | chronic urticaria, eczema, acne, rosacea, anxiety, diabetes, stress, smoking |
| TFP | ukb-a-290 | infections of the Skin and subcutaneous tissue | finn-b-L12_INFECT_SKIN | chronic urticaria, eczema, acne, rosacea, anxiety, diabetes, stress, smoking |
| WC | ebi-a-GCST90014020 | Prostatitis | finn-b-N14_PROSTATITIS | smoking, alcohol, lifetime number of sexual partners, sexual dysfunction |
| HC | ebi-a-GCST90014021 | Prostatitis | finn-b-N14_PROSTATITIS | smoking, alcohol, lifetime number of sexual partners, sexual dysfunction |
| BMI | ebi-a-GCST90018947 | Prostatitis | finn-b-N14_PROSTATITIS | smoking, alcohol, lifetime number of sexual partners, sexual dysfunction, anxiety, depression, stress, diabetes, cardiovascular disease, heart failure, hypertension, insulin resistance, hyperglycemia, cortisol, diuretics |
| TFP | ukb-a-290 | Prostatitis | finn-b-N14_PROSTATITIS | smoking, alcohol, lifetime number of sexual partners, sexual dysfunction |
| WC | ebi-a-GCST90014020 | Inguinal or femoral hernia, bilateral | finn-b-FEMINGHER_BILATERAL | constipation, pregnancy, cough on most days |
| HC | ebi-a-GCST90014021 | Inguinal or femoral hernia, bilateral | finn-b-FEMINGHER_BILATERAL | constipation, pregnancy, cough on most days |
| BMI | ebi-a-GCST90018947 | Inguinal or femoral hernia, bilateral | finn-b-FEMINGHER_BILATERAL | constipation, pregnancy, cough on most days |
| TFP | ukb-a-290 | Inguinal or femoral hernia, bilateral | finn-b-FEMINGHER_BILATERAL | constipation, pregnancy, cough on most days |
| WC | ebi-a-GCST90014020 | Insulin receptor protein | prot-a-1564 | gout, hyperthyroidism, hypothyroidism, pancreatitis, atherosclerosis, heart failure, sleep apnea, smoking, sleep disorders, anxiety, depression |
| HC | ebi-a-GCST90014021 | Insulin receptor protein | prot-a-1564 | gout, hyperthyroidism, hypothyroidism, pancreatitis, atherosclerosis, heart failure, sleep apnea, smoking, sleep disorders, anxiety, depression |
| BMI | ebi-a-GCST90018947 | Insulin receptor protein | prot-a-1564 | gout, hyperthyroidism, hypothyroidism, pancreatitis, atherosclerosis, heart failure, sleep apnea, smoking, sleep disorders, anxiety, depression |
| TFP | ukb-a-290 | Insulin receptor protein | prot-a-1564 | gout, hyperthyroidism, hypothyroidism, pancreatitis, atherosclerosis, heart failure, sleep apnea, smoking, sleep disorders, anxiety, depression |
| BMI | ukb-a-248 | Insulin resistance | ebi-a-GCST005179 | gout, hyperthyroidism, hypothyroidism, pancreatitis, atherosclerosis, heart failure, sleep apnea, smoking, sleep disorders, anxiety, depression, stroke |
| TFP | ukb-a-290 | Insulin resistance | ebi-a-GCST005179 | gout, hyperthyroidism, hypothyroidism, pancreatitis, atherosclerosis, heart failure, sleep apnea, smoking, sleep disorders, anxiety, depression, stroke |
| WC | ukb-a-382 | Insulin resistance | ebi-a-GCST005179 | gout, hyperthyroidism, hypothyroidism, pancreatitis, atherosclerosis, heart failure, sleep apnea, smoking, sleep disorders, anxiety, depression, stroke |
| HC | ukb-a-388 | Insulin resistance | ebi-a-GCST005179 | gout, hyperthyroidism, hypothyroidism, pancreatitis, atherosclerosis, heart failure, sleep apnea, smoking, sleep disorders, anxiety, depression, stroke |
| BMI | ukb-a-248 | Irritable bowel syndrome | ebi-a-GCST90016564 | thymoma, psoriasis, addiction |
| TFP | ukb-a-290 | Irritable bowel syndrome | ebi-a-GCST90016564 | thymoma, psoriasis, addiction |
| WC | ukb-a-382 | Irritable bowel syndrome | ebi-a-GCST90016564 | thymoma, psoriasis, addiction |
| HC | ukb-a-388 | Irritable bowel syndrome | ebi-a-GCST90016564 | thymoma, psoriasis, addiction |
| WC | ebi-a-GCST90014020 | Ischemic stroke | finn-b-I9_STR_EXH_EXNONE | nontraumatic intracranial haemmorrhage, hypertension, diabetes, sleep apnea syndrome, kidney disease, gout, heart failure, atrial fibrillation, left ventricular hypertrophy, smoking, depression |
| HC | ebi-a-GCST90014021 | Ischemic stroke | finn-b-I9_STR_EXH_EXNONE | nontraumatic intracranial haemmorrhage, hypertension, diabetes, sleep apnea syndrome, kidney disease, gout, heart failure, atrial fibrillation, left ventricular hypertrophy, smoking, depression |
| BMI | ebi-a-GCST90018947 | Ischemic stroke | finn-b-I9_STR_EXH_EXNONE | nontraumatic intracranial haemmorrhage, hypertension, diabetes, sleep apnea syndrome, kidney disease, gout, heart failure, atrial fibrillation, left ventricular hypertrophy, smoking, depression |
| TFP | ukb-a-290 | Ischemic stroke | finn-b-I9_STR_EXH_EXNONE | nontraumatic intracranial haemmorrhage, hypertension, diabetes, sleep apnea syndrome, kidney disease, gout, heart failure, atrial fibrillation, left ventricular hypertrophy, smoking, depression |
| WC | ebi-a-GCST90014020 | Intervertebral disk disorders | ukb-b-18279 | lumbar spine bone mineral density, smoking |
| HC | ebi-a-GCST90014021 | Intervertebral disk disorders | ukb-b-18279 | lumbar spine bone mineral density, smoking |
| BMI | ebi-a-GCST90018947 | Intervertebral disk disorders | ukb-b-18279 | lumbar spine bone mineral density, smoking |
| TFP | ukb-a-290 | Intervertebral disk disorders | ukb-b-18279 | lumbar spine bone mineral density, smoking |
| WC | ebi-a-GCST90014020 | Lumbar spine bone mineral density | ieu-a-982 | calcium deficiency, vitamin d deficiency, malnutrition |
| HC | ebi-a-GCST90014021 | Lumbar spine bone mineral density | ieu-a-982 | calcium deficiency, vitamin d deficiency, malnutrition |
| BMI | ebi-a-GCST90018947 | Lumbar spine bone mineral density | ieu-a-982 | calcium deficiency, vitamin d deficiency, malnutrition |
| TFP | ukb-a-290 | Lumbar spine bone mineral density | ieu-a-982 | calcium deficiency, vitamin d deficiency, malnutrition |
| BMI | ukb-a-248 | Lung adenocarcinoma | ebi-a-GCST004744 | alcohol, diabetes, smoking |
| TFP | ukb-a-290 | Lung adenocarcinoma | ebi-a-GCST004744 | alcohol, diabetes, smoking |
| WC | ukb-a-382 | Lung adenocarcinoma | ebi-a-GCST004744 | alcohol, diabetes, smoking |
| HC | ukb-a-388 | Lung adenocarcinoma | ebi-a-GCST004744 | alcohol, diabetes, smoking |
| WC | ebi-a-GCST90014020 | Malaise and fatigue | finn-b-R18_MALAI_FATIG | sleep disorders, urinary incontinence, depression, stress, infertility, hypertension, heart failure, diabetes, stroke, coronary atherosclerosis |
| HC | ebi-a-GCST90014021 | Malaise and fatigue | finn-b-R18_MALAI_FATIG | sleep disorders, urinary incontinence, depression, stress, infertility, hypertension, heart failure, diabetes, stroke, coronary atherosclerosis |
| BMI | ebi-a-GCST90018947 | Malaise and fatigue | finn-b-R18_MALAI_FATIG | sleep disorders, urinary incontinence, depression, stress, infertility, hypertension, heart failure, diabetes, stroke, coronary atherosclerosis |
| TFP | ukb-a-290 | Malaise and fatigue | finn-b-R18_MALAI_FATIG | sleep disorders, urinary incontinence, depression, stress, infertility, hypertension, heart failure, diabetes, stroke, coronary atherosclerosis |
| WC | ebi-a-GCST90014020 | Male infertility | finn-b-N14_MALEINFERT | thymoma, psoriasis, addiction |
| HC | ebi-a-GCST90014021 | Male infertility | finn-b-N14_MALEINFERT | thymoma, psoriasis, addiction |
| BMI | ebi-a-GCST90018947 | Male infertility | finn-b-N14_MALEINFERT | thymoma, psoriasis, addiction |
| TFP | ukb-a-290 | Male infertility | finn-b-N14_MALEINFERT | thymoma, psoriasis, addiction |
| WC | ebi-a-GCST90014020 | Malignant neoplasm of kidney | finn-b-C3_KIDNEY_NOTRENALPELVIS | gout, hypertension, heart failure, diabetes, smoking, kidney stones, renal calculi, pyelonephritis |
| HC | ebi-a-GCST90014021 | Malignant neoplasm of kidney | finn-b-C3_KIDNEY_NOTRENALPELVIS | gout, hypertension, heart failure, diabetes, smoking, kidney stones, renal calculi, pyelonephritis |
| BMI | ebi-a-GCST90018947 | Malignant neoplasm of kidney | finn-b-C3_KIDNEY_NOTRENALPELVIS | gout, hypertension, heart failure, diabetes, smoking, kidney stones, renal calculi, pyelonephritis |
| TFP | ukb-a-290 | Malignant neoplasm of kidney | finn-b-C3_KIDNEY_NOTRENALPELVIS | gout, hypertension, heart failure, diabetes, smoking, kidney stones, renal calculi, pyelonephritis |
| WC | ebi-a-GCST90014020 | Malignant neoplasm of prostate | finn-b-C3_PROSTATE_EXALLC | smoking, alcohol, lifetime number of sexual partners, sexual dysfunction |
| HC | ebi-a-GCST90014021 | Malignant neoplasm of prostate | finn-b-C3_PROSTATE_EXALLC | smoking, alcohol, lifetime number of sexual partners, sexual dysfunction |
| BMI | ebi-a-GCST90018947 | Malignant neoplasm of prostate | finn-b-C3_PROSTATE_EXALLC | smoking, alcohol, lifetime number of sexual partners, sexual dysfunction |
| TFP | ukb-a-290 | Malignant neoplasm of prostate | finn-b-C3_PROSTATE_EXALLC | smoking, alcohol, lifetime number of sexual partners, sexual dysfunction |
| WC | ebi-a-GCST90014020 | Metabolic disorders | finn-b-E4_METABOLIA | gout, atherosclerosis, hypertension, coronary heart disease, diabetes, sleep apnea, kidney disease, smoking |
| HC | ebi-a-GCST90014021 | Metabolic disorders | finn-b-E4_METABOLIA | gout, atherosclerosis, hypertension, coronary heart disease, diabetes, sleep apnea, kidney disease, smoking |
| BMI | ebi-a-GCST90018947 | Metabolic disorders | finn-b-E4_METABOLIA | gout, atherosclerosis, hypertension, coronary heart disease, diabetes, sleep apnea, kidney disease, smoking |
| TFP | ukb-a-290 | Metabolic disorders | finn-b-E4_METABOLIA | gout, atherosclerosis, hypertension, coronary heart disease, diabetes, sleep apnea, kidney disease, smoking |
| BMI | ukb-a-248 | Mild age-related type 2 diabetes | ebi-a-GCST90026416 | gout, hyperthyroidism, hypothyroidism, pancreatitis, atherosclerosis, heart failure, sleep apnea, smoking, sleep disorders, anxiety, depression, stroke |
| TFP | ukb-a-290 | Mild age-related type 2 diabetes | ebi-a-GCST90026416 | gout, hyperthyroidism, hypothyroidism, pancreatitis, atherosclerosis, heart failure, sleep apnea, smoking, sleep disorders, anxiety, depression, stroke |
| WC | ukb-a-382 | Mild age-related type 2 diabetes | ebi-a-GCST90026416 | gout, hyperthyroidism, hypothyroidism, pancreatitis, atherosclerosis, heart failure, sleep apnea, smoking, sleep disorders, anxiety, depression, stroke |
| HC | ukb-a-388 | Mild age-related type 2 diabetes | ebi-a-GCST90026416 | gout, hyperthyroidism, hypothyroidism, pancreatitis, atherosclerosis, heart failure, sleep apnea, smoking, sleep disorders, anxiety, depression, stroke |
| WC | ebi-a-GCST90014020 | Multiple sclerosis | finn-b-G6_MS | thymoma, psoriasis, addiction |
| HC | ebi-a-GCST90014021 | Multiple sclerosis | finn-b-G6_MS | thymoma, psoriasis, addiction |
| BMI | ebi-a-GCST90018947 | Multiple sclerosis | finn-b-G6_MS | thymoma, psoriasis, addiction |
| TFP | ukb-a-290 | Multiple sclerosis | finn-b-G6_MS | thymoma, psoriasis, addiction |
| BMI | ukb-a-248 | Myasthenia gravis | ebi-a-GCST90093061 | thymoma, psoriasis, addiction |
| TFP | ukb-a-290 | Myasthenia gravis | ebi-a-GCST90093061 | thymoma, psoriasis, addiction, insulin resistance, sleep disorders, anxiety, depression, stress, diabetes, cardiovascular disease, hypertension, kidney disease, pregnancy, pneumonia, thyroid cancer, hyperthyroidism, hypothyroidism, infection, sepsis |
| WC | ukb-a-382 | Myasthenia gravis | ebi-a-GCST90093061 | thymoma, psoriasis, addiction |
| HC | ukb-a-388 | Myasthenia gravis | ebi-a-GCST90093061 | thymoma, psoriasis, addiction |
| WC | ebi-a-GCST90014020 | Nonalcoholic fatty liver disease | finn-b-NAFLD | alcohol intake frequency, alcoholic drinks per week, alcohol usually taken with meals, diabetes, polycystic ovary syndrome, insulin resistance, sleep apnea syndrome, hypothyroidism |
| HC | ebi-a-GCST90014021 | Nonalcoholic fatty liver disease | finn-b-NAFLD | alcohol intake frequency, alcoholic drinks per week, alcohol usually taken with meals, diabetes, polycystic ovary syndrome, insulin resistance, sleep apnea syndrome, hypothyroidism |
| BMI | ebi-a-GCST90018947 | Nonalcoholic fatty liver disease | finn-b-NAFLD | alcohol intake frequency, alcoholic drinks per week, alcohol usually taken with meals, diabetes, polycystic ovary syndrome, insulin resistance, sleep apnea syndrome, hypothyroidism |
| TFP | ukb-a-290 | Nonalcoholic fatty liver disease | finn-b-NAFLD | alcohol intake frequency, alcoholic drinks per week, alcohol usually taken with meals, diabetes, polycystic ovary syndrome, insulin resistance, sleep apnea syndrome, hypothyroidism |
| WC | ebi-a-GCST90014020 | Nontraumatic intracranial haemmorrhage | finn-b-I9_INTRACRA | ischemic stroke, gout, hypertension, diabetes, sleep apnea syndrome, kidney disease, heart failure, atrial fibrillation, left ventricular hypertrophy, smoking, depression |
| HC | ebi-a-GCST90014021 | Nontraumatic intracranial haemmorrhage | finn-b-I9_INTRACRA | ischemic stroke, gout, hypertension, diabetes, sleep apnea syndrome, kidney disease, heart failure, atrial fibrillation, left ventricular hypertrophy, smoking, depression |
| BMI | ebi-a-GCST90018947 | Nontraumatic intracranial haemmorrhage | finn-b-I9_INTRACRA | ischemic stroke, gout, hypertension, diabetes, sleep apnea syndrome, kidney disease, heart failure, atrial fibrillation, left ventricular hypertrophy, smoking, depression |
| TFP | ukb-a-290 | Nontraumatic intracranial haemmorrhage | finn-b-I9_INTRACRA | ischemic stroke, gout, hypertension, diabetes, sleep apnea syndrome, kidney disease, heart failure, atrial fibrillation, left ventricular hypertrophy, smoking, depression |
| BMI | ukb-a-248 | Osteoarthritis | ebi-a-GCST90013881 | rheumatoid arthritis, gout, diabetes, hemochromatosis |
| TFP | ukb-a-290 | Osteoarthritis | ebi-a-GCST90013881 | rheumatoid arthritis, gout, diabetes, hemochromatosis |
| WC | ukb-a-382 | Osteoarthritis | ebi-a-GCST90013881 | rheumatoid arthritis, gout, diabetes, hemochromatosis |
| HC | ukb-a-388 | Osteoarthritis | ebi-a-GCST90013881 | rheumatoid arthritis, gout, diabetes, hemochromatosis |
| BMI | ukb-a-248 | Osteoporosis | ebi-a-GCST90038656 | hypocalcemia, calcium deficiency, vitamin d deficiency |
| TFP | ukb-a-290 | Osteoporosis | ebi-a-GCST90038656 | hypocalcemia, calcium deficiency, vitamin d deficiency |
| WC | ukb-a-382 | Osteoporosis | ebi-a-GCST90038656 | hypocalcemia, calcium deficiency, vitamin d deficiency, bipolar disorder, sleep disorders, depression, stress, hypoparathyroidism, radiation, alcohol intake frequency, alcoholic drinks per week, alcohol usually taken with meals, stress, C-reactive protein, hba1c, hyperglycemia, exercise, physical activity, educational attainment, income |
| HC | ukb-a-388 | Osteoporosis | ebi-a-GCST90038656 | hypocalcemia, calcium deficiency, vitamin d deficiency |
| BMI | ukb-a-248 | Parkinson's disease | ebi-a-GCST90018894 | stroke, hypertension, diabetes, smoking |
| TFP | ukb-a-290 | Parkinson's disease | ebi-a-GCST90018894 | stroke, hypertension, diabetes, smoking |
| WC | ukb-a-382 | Parkinson's disease | ebi-a-GCST90018894 | stroke, hypertension, diabetes, smoking |
| HC | ukb-a-388 | Parkinson's disease | ebi-a-GCST90018894 | stroke, hypertension, diabetes, smoking |
| WC | ebi-a-GCST90014020 | Peripheral atherosclerosis | finn-b-DM_PERIPHATHERO | gout, hypertension, diabetes, sleep apnea, kidney disease, smoking |
| HC | ebi-a-GCST90014021 | Peripheral atherosclerosis | finn-b-DM_PERIPHATHERO | gout, hypertension, diabetes, sleep apnea, kidney disease, smoking |
| BMI | ebi-a-GCST90018947 | Peripheral atherosclerosis | finn-b-DM_PERIPHATHERO | gout, hypertension, diabetes, sleep apnea, kidney disease, smoking |
| TFP | ukb-a-290 | Peripheral atherosclerosis | finn-b-DM_PERIPHATHERO | gout, hypertension, diabetes, sleep apnea, kidney disease, smoking |
| BMI | ukb-a-248 | Polycystic ovary syndrome | ebi-a-GCST90044902 | alcohol intake frequency, alcoholic drinks per week, alcohol usually taken with meals, diabetes, smoking |
| TFP | ukb-a-290 | Polycystic ovary syndrome | ebi-a-GCST90044902 | alcohol intake frequency, alcoholic drinks per week, alcohol usually taken with meals, diabetes, smoking |
| WC | ukb-a-382 | Polycystic ovary syndrome | ebi-a-GCST90044902 | alcohol intake frequency, alcoholic drinks per week, alcohol usually taken with meals, diabetes, smoking |
| HC | ukb-a-388 | Polycystic ovary syndrome | ebi-a-GCST90044902 | alcohol intake frequency, alcoholic drinks per week, alcohol usually taken with meals, diabetes, smoking |
| BMI | ukb-a-248 | Preeclampsia | ebi-a-GCST90018906 | essential hypertension, coronary heart disease, heart failure, diabetes, sleep apnea, kidney disease, left ventricular hypertrophy, smoking, anxiety disorders, bipolar disorder, sleep disorders, depression, alcohol intake frequency, alcoholic drinks per week, alcohol usually taken with meals, stress, C-reactive protein, hba1c, hyperglycemia, exercise, physical activity, educational attainment, income |
| TFP | ukb-a-290 | Preeclampsia | ebi-a-GCST90018906 | essential hypertension, coronary heart disease, heart failure, diabetes, sleep apnea, kidney disease, left ventricular hypertrophy, smoking, anxiety disorders, bipolar disorder, sleep disorders, depression |
| WC | ukb-a-382 | Preeclampsia | ebi-a-GCST90018906 | essential hypertension, coronary heart disease, heart failure, diabetes, sleep apnea, kidney disease, left ventricular hypertrophy, smoking, anxiety disorders, bipolar disorder, sleep disorders, depression |
| HC | ukb-a-388 | Preeclampsia | ebi-a-GCST90018906 | essential hypertension, coronary heart disease, heart failure, diabetes, sleep apnea, kidney disease, left ventricular hypertrophy, smoking, anxiety disorders, bipolar disorder, sleep disorders, depression |
| WC | ebi-a-GCST90014020 | Puerperal sepsis | finn-b-O15_PUERP_SEPSIS | tubal surgery, smoking, diabetes, anxiety, depression, stress, alcohol, diabetes, cancer |
| HC | ebi-a-GCST90014021 | Puerperal sepsis | finn-b-O15_PUERP_SEPSIS | tubal surgery, smoking, diabetes, anxiety, depression, stress, alcohol, diabetes, cancer |
| BMI | ebi-a-GCST90018947 | Puerperal sepsis | finn-b-O15_PUERP_SEPSIS | tubal surgery, smoking, diabetes, anxiety, depression, stress, alcohol, diabetes, cancer |
| TFP | ukb-a-290 | Puerperal sepsis | finn-b-O15_PUERP_SEPSIS | tubal surgery, smoking, diabetes, anxiety, depression, stress, alcohol, diabetes, cancer |
| BMI | ukb-a-248 | Pulmonary fibrosis | ebi-a-GCST90018908 | alcohol, diabetes, smoking |
| TFP | ukb-a-290 | Pulmonary fibrosis | ebi-a-GCST90018908 | alcohol, diabetes, smoking |
| WC | ukb-a-382 | Pulmonary fibrosis | ebi-a-GCST90018908 | alcohol, diabetes, smoking |
| HC | ukb-a-388 | Pulmonary fibrosis | ebi-a-GCST90018908 | alcohol, diabetes, smoking |
| WC | ebi-a-GCST90014020 | Pure hypercholesterolaemia | finn-b-E4_HYPERCHOL | alcohol, diabetes, cancer |
| HC | ebi-a-GCST90014021 | Pure hypercholesterolaemia | finn-b-E4_HYPERCHOL | alcohol, diabetes, cancer |
| BMI | ebi-a-GCST90018947 | Pure hypercholesterolaemia | finn-b-E4_HYPERCHOL | alcohol, diabetes, cancer |
| TFP | ukb-a-290 | Pure hypercholesterolaemia | finn-b-E4_HYPERCHOL | alcohol, diabetes, cancer |
| BMI | ukb-a-248 | Residual haemorrhoidal skin tags | ukb-b-16882 | anxiety disorders, bipolar disorder, malaise and fatigue, sleep disorders, depression, stress, infertility, hypertension, heart failure, smoking |
| TFP | ukb-a-290 | Residual haemorrhoidal skin tags | ukb-b-16882 | anxiety disorders, bipolar disorder, malaise and fatigue, sleep disorders, depression, stress, infertility, hypertension, heart failure, smoking |
| WC | ukb-a-382 | Residual haemorrhoidal skin tags | ukb-b-16882 | anxiety disorders, bipolar disorder, malaise and fatigue, sleep disorders, depression, stress, infertility, hypertension, heart failure, smoking |
| HC | ukb-a-388 | Residual haemorrhoidal skin tags | ukb-b-16882 | anxiety disorders, bipolar disorder, malaise and fatigue, sleep disorders, depression, stress, infertility, hypertension, heart failure, smoking |
| WC | ebi-a-GCST90014020 | Rheumatoid arthritis | finn-b-RHEUMA_SEROPOS | thymoma, psoriasis, addiction |
| HC | ebi-a-GCST90014021 | Rheumatoid arthritis | finn-b-RHEUMA_SEROPOS | thymoma, psoriasis, addiction |
| BMI | ebi-a-GCST90018947 | Rheumatoid arthritis | finn-b-RHEUMA_SEROPOS | thymoma, psoriasis, addiction |
| TFP | ukb-a-290 | Rheumatoid arthritis | finn-b-RHEUMA_SEROPOS | thymoma, psoriasis, addiction |
| WC | ebi-a-GCST90014020 | Rosacea | finn-b-L12_ROSACEA | acne, anxiety, metabolic disorders, depression, diabetes, stress, smoking |
| HC | ebi-a-GCST90014021 | Rosacea | finn-b-L12_ROSACEA | acne, anxiety, metabolic disorders, depression, diabetes, stress, smoking |
| BMI | ebi-a-GCST90018947 | Rosacea | finn-b-L12_ROSACEA | acne, anxiety, metabolic disorders, depression, diabetes, stress, smoking |
| TFP | ukb-a-290 | Rosacea | finn-b-L12_ROSACEA | acne, anxiety, metabolic disorders, depression, diabetes, stress, smoking |
| WC | ebi-a-GCST90014020 | Schizophrenia | finn-b-KRA_PSY_SCHIZODEL | thymoma, psoriasis, addiction |
| HC | ebi-a-GCST90014021 | Schizophrenia | finn-b-KRA_PSY_SCHIZODEL | thymoma, psoriasis, addiction |
| BMI | ebi-a-GCST90018947 | Schizophrenia | finn-b-KRA_PSY_SCHIZODEL | thymoma, psoriasis, addiction |
| TFP | ukb-a-290 | Schizophrenia | finn-b-KRA_PSY_SCHIZODEL | thymoma, psoriasis, addiction |
| WC | ebi-a-GCST90014020 | Sepsis | ieu-b-4980 | alcohol, diabetes, cancer |
| HC | ebi-a-GCST90014021 | Sepsis | ieu-b-4980 | alcohol, diabetes, cancer |
| BMI | ebi-a-GCST90018947 | Sepsis | ieu-b-4980 | alcohol, diabetes, cancer |
| TFP | ukb-a-290 | Sepsis | ieu-b-4980 | alcohol, diabetes, cancer |
| BMI | ukb-a-248 | Serum uric acid | ebi-a-GCST90018977 | kidney problem, renal failure, sleep disorders, anxiety, depression, stress, diabetes, cardiovascular disease, heart failure, hypertension, insulin resistance, hyperglycemia, inflammatory, alcohol, pregnancy, pneumonia, thyroid cancer, hyperthyroidism, hypothyroidism, infection, sepsis, cortisol, diuretics, beta blocking agents, aspirin, physical activity, smoking, renal disease, purine, yeast, C-reactive protein, hba1c, hyperglycemia, exercise, procalcitonin, il-6, tnf, atp-binding cassette transporters,igf-1, neutrophil cell count, gamma glutamyltransferase levels, cancer, leukemia, lymphoma, multiple myeloma, hemolytic anemia, myelofibrosis, metabolic disorder, fasting glucose, glycated haemoglobin hba1c levels, resistance, resistin levels, sleep apnea syndrome, educational attainment, income, stroke, nonalcoholic fatty liver disease, kidney stones, renal calculi, pyelonephritis, eczema, thyrotropin-releasing hormone, corticotropin-releasing factor-binding protein |
| TFP | ukb-a-290 | Serum uric acid | ebi-a-GCST90018977 | kidney problem, renal failure, sleep disorders, anxiety, depression, stress, diabetes, cardiovascular disease, heart failure, hypertension, insulin resistance, hyperglycemia, inflammatory, alcohol, pregnancy, pneumonia, thyroid cancer, hyperthyroidism, hypothyroidism, infection, sepsis, cortisol, diuretics |
| WC | ukb-a-382 | Serum uric acid | ebi-a-GCST90018977 | hypothyroidism, kidney problem, renal failure |
| HC | ukb-a-388 | Serum uric acid | ebi-a-GCST90018977 | kidney problem, renal failure, sleep disorders, anxiety, depression, stress, diabetes, cardiovascular disease, heart failure, hypertension, insulin resistance, hyperglycemia, inflammatory, alcohol, pregnancy, pneumonia, thyroid cancer, hyperthyroidism, hypothyroidism, infection, sepsis, cortisol, diuretics, beta blocking agents, aspirin, physical activity, smoking, renal disease, purine, yeast, C-reactive protein, hba1c, hyperglycemia, exercise, procalcitonin, il-6, tnf, atp-binding cassette transporters, igf-2, neutrophil cell count, gamma glutamyltransferase levels, cancer, leukemia, lymphoma, multiple myeloma, hemolytic anemia, myelofibrosis |
| BMI | ukb-a-248 | Sex hormone binding globulin | ebi-a-GCST90014011 | anxiety disorders, bipolar disorder, malaise and fatigue, sleep disorders, depression, stress, hypertension, heart failure, smoking, gout, hypertension, coronary heart disease, diabetes, sleep apnea, kidney disease, hyperthyroidism, alcohol intake frequency, alcoholic drinks per week, alcohol usually taken with meals, anxiety disorders, C-reactive protein, hba1c, hyperglycemia, exercise, physical activity, educational attainment, income |
| TFP | ukb-a-290 | Sex hormone binding globulin | ebi-a-GCST90014011 | anxiety disorders, bipolar disorder, malaise and fatigue, sleep disorders, depression, stress, hypertension, heart failure, smoking |
| WC | ukb-a-382 | Sex hormone binding globulin | ebi-a-GCST90014011 | anxiety disorders, bipolar disorder, malaise and fatigue, chronic fatigue syndrome, sleep disorders, depression, stress, hypertension, heart failure, smoking, gout, coronary heart disease, diabetes, insulin resistance, sleep apnea, hyperthyroidism ,hypothyroidism, alcohol intake frequency, alcoholic drinks per week, alcohol usually taken with meals, C-reactive protein, hba1c, hyperglycemia, exercise, physical activity, educational attainment, income ,contraceptives, birth control pills, hepatitis, kidney disease, kidney failure, serum albumin levels |
| HC | ukb-a-388 | Sex hormone binding globulin | ebi-a-GCST90014011 | anxiety disorders, bipolar disorder, malaise and fatigue, sleep disorders, depression, stress, hypertension, heart failure, smoking |
| BMI | ukb-a-248 | Sleep apnea syndrome | ebi-a-GCST90018916 | alcohol intake frequency, alcoholic drinks per week, alcohol usually taken with meals, sinusitis, anxiety disorders, bipolar disorder, malaise and fatigue, sleep disorders, depression, stress, hypertension, heart failure, smoking, gout, hypertension, coronary heart disease, diabetes, sleep apnea, kidney disease, hyperthyroidism, C-reactive protein, hba1c, hyperglycemia, exercise, physical activity, educational attainment, income |
| TFP | ukb-a-290 | Sleep apnea syndrome | ebi-a-GCST90018916 | alcohol intake frequency, alcoholic drinks per week, alcohol usually taken with meals, sinusitis |
| WC | ukb-a-382 | Sleep apnea syndrome | ebi-a-GCST90018916 | alcohol intake frequency, alcoholic drinks per week, alcohol usually taken with meals, sinusitis |
| HC | ukb-a-388 | Sleep apnea syndrome | ebi-a-GCST90018916 | alcohol intake frequency, alcoholic drinks per week, alcohol usually taken with meals, sinusitis |
| WC | ebi-a-GCST90014020 | Sleep disorders | finn-b-SLEEP | malaise and fatigue, urinary incontinence, depression, stress, infertility, hypertension, heart failure, diabetes, stroke, coronary atherosclerosis |
| HC | ebi-a-GCST90014021 | Sleep disorders | finn-b-SLEEP | malaise and fatigue, urinary incontinence, depression, stress, infertility, hypertension, heart failure, diabetes, stroke, coronary atherosclerosis |
| BMI | ebi-a-GCST90018947 | Sleep disorders | finn-b-SLEEP | malaise and fatigue, urinary incontinence, depression, stress, infertility, hypertension, heart failure, diabetes, stroke, coronary atherosclerosis |
| TFP | ukb-a-290 | Sleep disorders | finn-b-SLEEP | malaise and fatigue, urinary incontinence, depression, stress, infertility, hypertension, heart failure, diabetes, stroke, coronary atherosclerosis |
| BMI | ukb-a-248 | Sporadic miscarriage | ebi-a-GCST011888 | ectopic pregnancy, puerperal sepsis,pelvic inflammatory, tubal surgery, smoking, diabetes |
| TFP | ukb-a-290 | Sporadic miscarriage | ebi-a-GCST011888 | ectopic pregnancy, puerperal sepsis,pelvic inflammatory, tubal surgery, smoking, diabetes |
| WC | ukb-a-382 | Sporadic miscarriage | ebi-a-GCST011888 | ectopic pregnancy, puerperal sepsis,pelvic inflammatory, tubal surgery, smoking, diabetes |
| HC | ukb-a-388 | Sporadic miscarriage | ebi-a-GCST011888 | ectopic pregnancy, puerperal sepsis,pelvic inflammatory, tubal surgery, smoking, diabetes |
| WC | ebi-a-GCST90014020 | Stroke | finn-b-I9_STR_SAH | hypertension, diabetes, sleep apnea syndrome, kidney disease, heart failure, atrial fibrillation, left ventricular hypertrophy, smoking, depression, gout |
| HC | ebi-a-GCST90014021 | Stroke | finn-b-I9_STR_SAH | hypertension, diabetes, sleep apnea syndrome, kidney disease, heart failure, atrial fibrillation, left ventricular hypertrophy, smoking, depression, gout |
| BMI | ebi-a-GCST90018947 | Stroke | finn-b-I9_STR_SAH | hypertension, diabetes, sleep apnea syndrome, kidney disease, heart failure, atrial fibrillation, left ventricular hypertrophy, smoking, depression, gout |
| TFP | ukb-a-290 | Stroke | finn-b-I9_STR_SAH | hypertension, diabetes, sleep apnea syndrome, kidney disease, heart failure, atrial fibrillation, left ventricular hypertrophy, smoking, depression, gout |
| BMI | ukb-a-248 | Systemic lupus erythematosus | ebi-a-GCST90018917 | thymoma, psoriasis, addiction |
| TFP | ukb-a-290 | Systemic lupus erythematosus | ebi-a-GCST90018917 | thymoma, psoriasis, addiction |
| WC | ukb-a-382 | Systemic lupus erythematosus | ebi-a-GCST90018917 | thymoma, psoriasis, addiction |
| HC | ukb-a-388 | Systemic lupus erythematosus | ebi-a-GCST90018917 | thymoma, psoriasis, addiction |
| WC | ebi-a-GCST90014020 | Systemic sclerosis | finn-b-SYSTSCLE_STRICT | thymoma, psoriasis, addiction |
| HC | ebi-a-GCST90014021 | Systemic sclerosis | finn-b-SYSTSCLE_STRICT | thymoma, psoriasis, addiction |
| BMI | ebi-a-GCST90018947 | Systemic sclerosis | finn-b-SYSTSCLE_STRICT | thymoma, psoriasis, addiction |
| TFP | ukb-a-290 | Systemic sclerosis | finn-b-SYSTSCLE_STRICT | thymoma, psoriasis, addiction |
| BMI | ukb-a-248 | Total body bone mineral density (age 30–45) | ebi-a-GCST005346 | calcium deficiency, vitamin d deficiency, malnutrition |
| TFP | ukb-a-290 | Total body bone mineral density (age 30–45) | ebi-a-GCST005346 | calcium deficiency, vitamin d deficiency, malnutrition |
| WC | ukb-a-382 | Total body bone mineral density (age 30–45) | ebi-a-GCST005346 | calcium deficiency, vitamin d deficiency, malnutrition |
| HC | ukb-a-388 | Total body bone mineral density (age 30–45) | ebi-a-GCST005346 | calcium deficiency, vitamin d deficiency, malnutrition |
| BMI | ukb-a-248 | Total body bone mineral density (age 45–60) | ebi-a-GCST005350 | calcium deficiency, vitamin d deficiency, malnutrition |
| TFP | ukb-a-290 | Total body bone mineral density (age 45–60) | ebi-a-GCST005350 | calcium deficiency, vitamin d deficiency, malnutrition |
| WC | ukb-a-382 | Total body bone mineral density (age 45–60) | ebi-a-GCST005350 | calcium deficiency, vitamin d deficiency, malnutrition |
| HC | ukb-a-388 | Total body bone mineral density (age 45–60) | ebi-a-GCST005350 | calcium deficiency, vitamin d deficiency, malnutrition, estrogen, testosterone, growth hormone, anxiety, depression, stress, diabetes, cardiovascular disease, heart failure, hypertension, insulin resistance, hyperglycemia, inflammatory, alcohol, pregnancy, lactation, pneumonia, thyroid cancer, hyperthyroidism, hypothyroidism, infection, sepsis, cortisol |
| BMI | ukb-a-248 | Total body bone mineral density (age over 60) | ebi-a-GCST005349 | calcium deficiency, vitamin d deficiency, malnutrition |
| TFP | ukb-a-290 | Total body bone mineral density (age over 60) | ebi-a-GCST005349 | calcium deficiency, vitamin d deficiency, malnutrition |
| WC | ukb-a-382 | Total body bone mineral density (age over 60) | ebi-a-GCST005349 | calcium deficiency, vitamin d deficiency, malnutrition |
| HC | ukb-a-388 | Total body bone mineral density (age over 60) | ebi-a-GCST005349 | calcium deficiency, vitamin d deficiency, malnutrition |
| BMI | ukb-a-248 | Type 1 diabetes | ebi-a-GCST90013891 | gout, hyperthyroidism, hypothyroidism, pancreatitis, atherosclerosis, heart failure, sleep apnea, smoking, sleep disorders, anxiety, depression, stroke |
| TFP | ukb-a-290 | Type 1 diabetes | ebi-a-GCST90013891 | gout, hyperthyroidism, hypothyroidism, pancreatitis, atherosclerosis, heart failure, sleep apnea, smoking, sleep disorders, anxiety, depression, stroke |
| WC | ukb-a-382 | Type 1 diabetes | ebi-a-GCST90013891 | gout, hyperthyroidism, hypothyroidism, pancreatitis, atherosclerosis, heart failure, sleep apnea, smoking, sleep disorders, anxiety, depression, stroke |
| HC | ukb-a-388 | Type 1 diabetes | ebi-a-GCST90013891 | gout, hyperthyroidism, hypothyroidism, pancreatitis, atherosclerosis, heart failure, sleep apnea, smoking, sleep disorders, anxiety, depression, stroke |
| WC | ebi-a-GCST90014020 | Type 2 diabetes | finn-b-E4_DM2_STRICT | gout, hyperthyroidism, hypothyroidism, pancreatitis, atherosclerosis, heart failure, sleep apnea, smoking, sleep disorders, anxiety, depression, stroke |
| HC | ebi-a-GCST90014021 | Type 2 diabetes | finn-b-E4_DM2_STRICT | gout, hyperthyroidism, hypothyroidism, pancreatitis, atherosclerosis, heart failure, sleep apnea, smoking, sleep disorders, anxiety, depression, stroke |
| BMI | ebi-a-GCST90018947 | Type 2 diabetes | finn-b-E4_DM2_STRICT | gout, hyperthyroidism, hypothyroidism, pancreatitis, atherosclerosis, heart failure, sleep apnea, smoking, sleep disorders, anxiety, depression, stroke |
| TFP | ukb-a-290 | Type 2 diabetes | finn-b-E4_DM2_STRICT | gout, hyperthyroidism, hypothyroidism, pancreatitis, atherosclerosis, heart failure, sleep apnea, smoking, sleep disorders, anxiety, depression, stroke ,alcohol intake frequency, alcoholic drinks per week, alcohol usually taken with meals, stress, C-reactive protein, exercise, physical activity, educational attainment, income, cortisol levels, glucocorticoids, familial combined hyperlipidemia, intake of sugar added to cereal, intake of sugar added to coffee, intake of sugar added to tea |
| WC | ebi-a-GCST90014020 | Ulcerative colitis | finn-b-K11_ULCER | thymoma, psoriasis, addiction |
| HC | ebi-a-GCST90014021 | Ulcerative colitis | finn-b-K11_ULCER | thymoma, psoriasis, addiction |
| BMI | ebi-a-GCST90018947 | Ulcerative colitis | finn-b-K11_ULCER | thymoma, psoriasis, addiction |
| TFP | ukb-a-290 | Ulcerative colitis | finn-b-K11_ULCER | thymoma, psoriasis, addiction |
| WC | ebi-a-GCST90014020 | Urinary incontinence | finn-b-R18_UNSPE_URINARY_INCONTINENCE | malaise and fatigue, sleep disorders, depression, stress, infertility, hypertension, heart failure, diabetes, stroke, coronary atherosclerosis |
| HC | ebi-a-GCST90014021 | Urinary incontinence | finn-b-R18_UNSPE_URINARY_INCONTINENCE | malaise and fatigue, sleep disorders, depression, stress, infertility, hypertension, heart failure, diabetes, stroke, coronary atherosclerosis |
| BMI | ebi-a-GCST90018947 | Urinary incontinence | finn-b-R18_UNSPE_URINARY_INCONTINENCE | malaise and fatigue, sleep disorders, depression, stress, infertility, hypertension, heart failure, diabetes, stroke, coronary atherosclerosis, menopausal, perimenopausal, alcohol intake frequency, alcoholic drinks per week, alcohol usually taken with meals, smoking, C-reactive protein, hba1c, hyperglycemia, exercise, physical activity, educational attainment, income, cough on most days, chronic obstructive pulmonary disease, constipation, diuretics, urinary tract infections |
| TFP | ukb-a-290 | Urinary incontinence | finn-b-R18_UNSPE_URINARY_INCONTINENCE | malaise and fatigue, sleep disorders, depression, stress, infertility, hypertension, heart failure, diabetes, stroke, coronary atherosclerosis |
| BMI | ukb-a-248 | Urinary tract infection or kidney infection | ebi-a-GCST90013890 | alcohol, diabetes, smoking |
| TFP | ukb-a-290 | Urinary tract infection or kidney infection | ebi-a-GCST90013890 | alcohol, diabetes, smoking |
| WC | ukb-a-382 | Urinary tract infection or kidney infection | ebi-a-GCST90013890 | alcohol, diabetes, smoking |
| HC | ukb-a-388 | Urinary tract infection or kidney infection | ebi-a-GCST90013890 | alcohol, diabetes, smoking |
| WC | ebi-a-GCST90014020 | Varicose veins | finn-b-I9_VARICVE_EXNONE | pregnancy, hypertension, heart failure, diabetes, smoking |
| HC | ebi-a-GCST90014021 | Varicose veins | finn-b-I9_VARICVE_EXNONE | pregnancy, hypertension, heart failure, diabetes, smoking |
| BMI | ebi-a-GCST90018947 | Varicose veins | finn-b-I9_VARICVE_EXNONE | pregnancy, hypertension, heart failure, diabetes, smoking |
| TFP | ukb-a-290 | Varicose veins | finn-b-I9_VARICVE_EXNONE | pregnancy, hypertension, heart failure, diabetes, smoking |

Abbreviations: WC, waist circumference; HC, hip circumference; TFP, total fat percentage; BMI, body mass index; GWAS, Genome-Wide association study.
