# Supplementary material for: Unraveling the causal web of 4 adiposity indices and 92 multi-system outcomes: A body-wide Mendelian randomization study
Source: Medicine (Baltimore). 2026 May 22;105(21):e48986. doi: 10.1097/MD.0000000000048986 (PMC13201005; doi:10.1097/MD.0000000000048986)
Supplement: Supplementary file 5 [file medi-105-e48986-s005.docx]

Table S5. Results of two-sample Mendelian randomization analyses on the causal effects of adiposity indices with 92 outcomes.

| **Outcomes** | **Exposure** | **nSNP** | **F-statistic** | **Methods** | **p-value** | **Effect size (95%CI)** |
| --- | --- | --- | --- | --- | --- | --- |
| **Cardiovascular and metabolic disorders** | | | | | | |
| Atrial fibrillation and flutter | BMI | 118 | 24.06 | MR Egger | 0.0079 | 2.388 (1.267, 4.499) |
|  |  |  |  | Inverse variance weighted | 2.09E-07 | 1.680 (1.381, 2.043) |
|  |  |  |  | Maximum likelihood | 1.03E-09 | 1.701 (1.434, 2.017) |
| Atrial fibrillation and flutter | HC | 98 | 28.49 | MR Egger | 0.6960 | 1.110 (0.659, 1.869) |
|  |  |  |  | Inverse variance weighted | 4.89E-07 | 1.608 (1.336, 1.935) |
|  |  |  |  | Maximum likelihood | 1.51E-10 | 1.633 (1.406, 1.898) |
| Atrial fibrillation and flutter | TFP | 160 | 22.35 | MR Egger | 0.9150 | 0.944 (0.328, 2.714) |
|  |  |  |  | Inverse variance weighted | 0.0222 | 1.318 (1.040, 1.671) |
|  |  |  |  | Maximum likelihood | 0.0075 | 1.327 (1.078, 1.632) |
| Atrial fibrillation and flutter | WC | 234 | 28.36 | MR Egger | 0.0188 | 2.480 (1.179, 5.215) |
|  |  |  |  | Inverse variance weighted (fixed effects) | 4.47E-10 | 2.043 (1.632, 2.557) |
|  |  |  |  | Maximum likelihood | 3.47E-10 | 2.073 (1.651, 2.602) |
| Cerebral atherosclerosis | BMI | 125 | 23.68 | MR Egger | 0.5258 | 0.183 (0.001, 34.398) |
|  |  |  |  | Inverse variance weighted (fixed effects) | 0.5066 | 1.718 (0.348, 8.483) |
|  |  |  |  | Maximum likelihood | 0.5034 | 1.733 (0.346, 8.681) |
| Cerebral atherosclerosis | HC | 96 | 28.59 | MR Egger | 0.7708 | 0.546 (0.009, 31.721) |
|  |  |  |  | Inverse variance weighted (fixed effects) | 0.7006 | 1.315 (0.326, 5.311) |
|  |  |  |  | Maximum likelihood | 0.6985 | 1.321 (0.323, 5.399) |
| Cerebral atherosclerosis | TFP | 118 | 22.74 | MR Egger | 0.3337 | 0.008 (0.000, 129.650) |
|  |  |  |  | Inverse variance weighted | 0.6396 | 1.678 (0.192, 14.665) |
|  |  |  |  | Maximum likelihood | 0.5949 | 1.690 (0.244, 11.708) |
| Cerebral atherosclerosis | WC | 136 | 28.26 | MR Egger | 0.2287 | 0.014 (0.000, 13.838) |
|  |  |  |  | Inverse variance weighted (fixed effects) | 0.2835 | 0.336 (0.046, 2.463) |
|  |  |  |  | Maximum likelihood | 0.2851 | 0.334 (0.045, 2.495) |
| Coronary atherosclerosis | BMI | 119 | 23.88 | MR Egger | 0.1811 | 1.463 (0.840, 2.547) |
|  |  |  |  | Inverse variance weighted | 0.0564 | 1.186 (0.995, 1.414) |
|  |  |  |  | Maximum likelihood | 0.0252 | 1.192 (1.022, 1.391) |
| Coronary atherosclerosis | HC | 98 | 28.31 | MR Egger | 0.4830 | 1.161 (0.766, 1.759) |
|  |  |  |  | Inverse variance weighted | 0.5521 | 1.045 (0.904, 1.209) |
|  |  |  |  | Maximum likelihood | 0.4994 | 1.046 (0.918, 1.192) |
| Coronary atherosclerosis | TFP | 77 | 23.97 | MR Egger | 0.2111 | 0.012 (0.000, 11.652) |
|  |  |  |  | Inverse variance weighted (fixed effects) | 0.8424 | 0.836 (0.143, 4.875) |
|  |  |  |  | Maximum likelihood | 0.8408 | 0.833 (0.141, 4.937) |
| Coronary atherosclerosis | WC | 144 | 28.25 | MR Egger | 0.0602 | 2.016 (0.979, 4.151) |
|  |  |  |  | Inverse variance weighted | 0.0001 | 1.530 (1.228, 1.906) |
|  |  |  |  | Maximum likelihood | 5.66E-06 | 1.545 (1.281, 1.865) |
| Coronary heart disease | BMI | 113 | 23.88 | MR Egger | 0.6114 | 1.154 (0.665, 2.004) |
|  |  |  |  | Inverse variance weighted | 0.0254 | 1.220 (1.025, 1.452) |
|  |  |  |  | Maximum likelihood | 0.0119 | 1.225 (1.046, 1.436) |
| Coronary heart disease | HC | 91 | 28.37 | MR Egger | 0.8318 | 0.956 (0.633, 1.445) |
|  |  |  |  | Inverse variance weighted (fixed effects) | 0.8657 | 1.011 (0.886, 1.155) |
|  |  |  |  | Maximum likelihood | 0.8645 | 1.012 (0.885, 1.156) |
| Coronary heart disease | TFP | 63 | 22.98 | MR Egger | 0.8113 | 0.881 (0.311, 2.493) |
|  |  |  |  | Inverse variance weighted | 0.2293 | 1.155 (0.913, 1.462) |
|  |  |  |  | Maximum likelihood | 0.1227 | 1.159 (0.961, 1.398) |
| Coronary heart disease | WC | 146 | 28.25 | MR Egger | 0.2564 | 1.505 (0.746, 3.033) |
|  |  |  |  | Inverse variance weighted (fixed effects) | 0.0009 | 1.381 (1.141, 1.671) |
|  |  |  |  | Maximum likelihood | 0.0007 | 1.395 (1.150, 1.693) |
| Gestational diabetes | BMI | 255 | 25.04 | MR Egger | 0.1219 | 1.851 (0.852, 4.022) |
|  |  |  |  | Inverse variance weighted (fixed effects) | 0.0001 | 1.524 (1.236, 1.878) |
|  |  |  |  | Maximum likelihood | 0.0001 | 1.535 (1.243, 1.897) |
| Gestational diabetes | HC | 176 | 30.16 | MR Egger | 0.4024 | 0.764 (0.408, 1.431) |
|  |  |  |  | Inverse variance weighted | 0.2710 | 1.124 (0.913, 1.383) |
|  |  |  |  | Maximum likelihood | 0.1917 | 1.128 (0.942, 1.350) |
| Gestational diabetes | TFP | 81 | 23.17 | MR Egger | 0.5800 | 1.421 (0.411, 4.908) |
|  |  |  |  | Inverse variance weighted | 0.1248 | 1.240 (0.942, 1.632) |
|  |  |  |  | Maximum likelihood | 0.0751 | 1.245 (0.978, 1.584) |
| Gestational diabetes | WC | 138 | 27.69 | MR Egger | 0.0504 | 2.435 (1.007, 5.888) |
|  |  |  |  | Inverse variance weighted (fixed effects) | 3.04E-05 | 1.700 (1.325, 2.181) |
|  |  |  |  | Maximum likelihood | 3.50E-05 | 1.703 (1.323, 2.191) |
| Heart failure | BMI | 62 | 23.82 | MR Egger | 0.0922 | 1.667 (0.923, 3.011) |
|  |  |  |  | Inverse variance weighted (fixed effects) | 2.07E-07 | 1.548 (1.313, 1.825) |
|  |  |  |  | Maximum likelihood | 1.64E-07 | 1.563 (1.322, 1.847) |
| Heart failure | HC | 80 | 28.6 | MR Egger | 0.5239 | 1.147 (0.752, 1.750) |
|  |  |  |  | Inverse variance weighted (fixed effects) | 3.53E-08 | 1.500 (1.299, 1.733) |
|  |  |  |  | Maximum likelihood | 2.60E-08 | 1.512 (1.307, 1.750) |
| Heart failure | TFP | 97 | 22.57 | MR Egger | 0.8677 | 1.091 (0.394, 3.020) |
|  |  |  |  | Inverse variance weighted | 0.0158 | 1.325 (1.054, 1.665) |
|  |  |  |  | Maximum likelihood | 0.0047 | 1.337 (1.093, 1.635) |
| Heart failure | WC | 236 | 28.26 | MR Egger | 0.0015 | 3.092 (1.568, 6.097) |
|  |  |  |  | Inverse variance weighted (fixed effects) | 2.13E-08 | 1.799 (1.465, 2.210) |
|  |  |  |  | Maximum likelihood | 9.33E-09 | 1.836 (1.492, 2.260) |
| Hypertension | BMI | 93 | 22.52 | MR Egger | 0.2269 | 1.457 (0.793, 2.675) |
|  |  |  |  | Inverse variance weighted | 2.13E-07 | 1.519 (1.297, 1.780) |
|  |  |  |  | Maximum likelihood | 5.67E-12 | 1.533 (1.357, 1.731) |
| Hypertension | HC | 181 | 29.26 | MR Egger | 0.4536 | 0.842 (0.539, 1.317) |
|  |  |  |  | Inverse variance weighted | 0.7309 | 1.027 (0.884, 1.193) |
|  |  |  |  | Maximum likelihood | 0.6244 | 1.028 (0.921, 1.147) |
| Hypertension | TFP | 84 | 22.27 | MR Egger | 0.4184 | 0.708 (0.309, 1.625) |
|  |  |  |  | Inverse variance weighted | 0.0190 | 1.252 (1.038, 1.511) |
|  |  |  |  | Maximum likelihood | 0.0013 | 1.261 (1.094, 1.452) |
| Hypertension | WC | 173 | 27.08 | MR Egger | 0.6569 | 1.151 (0.620, 2.135) |
|  |  |  |  | Inverse variance weighted | 8.13E-05 | 1.397 (1.183, 1.649) |
|  |  |  |  | Maximum likelihood | 4.11E-06 | 1.407 (1.217, 1.627) |
| Insulin receptor protein | BMI | 104 | 25.98 | MR Egger | 0.4787 | 0.254 (−0.448, 0.956) |
|  |  |  |  | Inverse variance weighted (fixed effects) | 0.1562 | −0.160 (−0.383, 0.061) |
|  |  |  |  | Maximum likelihood | 0.1581 | −0.161 (−0.386, 0.063) |
| Insulin receptor protein | HC | 179 | 29.6 | MR Egger | 0.4424 | −0.251 (−0.889, 0.387) |
|  |  |  |  | Inverse variance weighted (fixed effects) | 0.4589 | −0.077 (−0.281, 0.127) |
|  |  |  |  | Maximum likelihood | 0.4599 | −0.077 (−0.282, 0.128) |
| Insulin receptor protein | TFP | 112 | 22.52 | MR Egger | 0.1739 | −0.833 (−2.025, 0.360) |
|  |  |  |  | Inverse variance weighted (fixed effects) | 0.1257 | −0.217 (−0.496, 0.061) |
|  |  |  |  | Maximum likelihood | 0.1225 | −0.221 (−0.502, 0.059) |
| Insulin receptor protein | WC | 32 | 27.98 | MR Egger | 0.9168 | −0.053 (−1.061, 0.954) |
|  |  |  |  | Inverse variance weighted (fixed effects) | 0.9851 | −0.003 (−0.289, 0.284) |
|  |  |  |  | Maximum likelihood | 0.9855 | −0.003 (−0.292, 0.287) |
| Metabolic disorders | BMI | 315 | 23.88 | MR Egger | 0.5421 | 1.140 (0.748, 1.738) |
|  |  |  |  | Inverse variance weighted (fixed effects) | 0.8420 | 1.012 (0.897, 1.143) |
|  |  |  |  | Maximum likelihood | 0.8407 | 1.013 (0.896, 1.144) |
| Metabolic disorders | HC | 182 | 28.47 | MR Egger | 0.1738 | 0.804 (0.587, 1.100) |
|  |  |  |  | Inverse variance weighted (fixed effects) | 0.3589 | 0.952 (0.857, 1.058) |
|  |  |  |  | Maximum likelihood | 0.3604 | 0.952 (0.855, 1.058) |
| Metabolic disorders | TFP | 148 | 22.92 | MR Egger | 0.7177 | 0.865 (0.395, 1.893) |
|  |  |  |  | Inverse variance weighted | 0.2263 | 1.115 (0.935, 1.329) |
|  |  |  |  | Maximum likelihood | 0.1502 | 1.118 (0.961, 1.300) |
| Metabolic disorders | WC | 250 | 28.13 | MR Egger | 0.8360 | 1.065 (0.585, 1.940) |
|  |  |  |  | Inverse variance weighted | 0.8228 | 1.021 (0.853, 1.222) |
|  |  |  |  | Maximum likelihood | 0.7878 | 1.021 (0.876, 1.190) |
| Peripheral atherosclerosis | BMI | 317 | 22.68 | MR Egger | 0.7565 | 0.851 (0.307, 2.359) |
|  |  |  |  | Inverse variance weighted | 0.3533 | 1.136 (0.868, 1.487) |
|  |  |  |  | Maximum likelihood | 0.2770 | 1.140 (0.900, 1.444) |
| Peripheral atherosclerosis | HC | 182 | 27.85 | MR Egger | 0.4977 | 1.273 (0.634, 2.556) |
|  |  |  |  | Inverse variance weighted | 0.0017 | 1.447 (1.149, 1.823) |
|  |  |  |  | Maximum likelihood | 0.0003 | 1.461 (1.190, 1.794) |
| Peripheral atherosclerosis | TFP | 59 | 22.5 | MR Egger | 0.2406 | 2.227 (0.591, 8.398) |
|  |  |  |  | Inverse variance weighted (fixed effects) | 0.0030 | 1.534 (1.157, 2.035) |
|  |  |  |  | Maximum likelihood | 0.0025 | 1.553 (1.167, 2.067) |
| Peripheral atherosclerosis | WC | 65 | 27.16 | MR Egger | 0.1213 | 2.490 (0.793, 7.819) |
|  |  |  |  | Inverse variance weighted (fixed effects) | 1.06E-06 | 2.056 (1.539, 2.746) |
|  |  |  |  | Maximum likelihood | 8.71E-07 | 2.086 (1.556, 2.796) |
| Type 2 diabetes | BMI | 160 | 22.79 | MR Egger | 0.0285 | 2.302 (1.099, 4.819) |
|  |  |  |  | Inverse variance weighted | 2.54E-18 | 2.059 (1.751, 2.421) |
|  |  |  |  | Maximum likelihood | 7.06E-31 | 2.124 (1.869, 2.413) |
| Type 2 diabetes | HC | 165 | 28.29 | MR Egger | 0.6197 | 0.879 (0.530, 1.460) |
|  |  |  |  | Inverse variance weighted | 0.0001 | 1.364 (1.162, 1.601) |
|  |  |  |  | Maximum likelihood | 2.70E-09 | 1.377 (1.239, 1.530) |
| Type 2 diabetes | TFP | 63 | 22.17 | MR Egger | 0.6265 | 0.764 (0.259, 2.251) |
|  |  |  |  | Inverse variance weighted | 0.0250 | 1.347 (1.038, 1.747) |
|  |  |  |  | Maximum likelihood | 0.0005 | 1.365 (1.145, 1.627) |
| Type 2 diabetes | WC | 115 | 26.6 | MR Egger | 0.0173 | 2.347 (1.174, 4.690) |
|  |  |  |  | Inverse variance weighted | 9.07E-23 | 2.451 (2.050, 2.931) |
|  |  |  |  | Maximum likelihood | 7.45E-33 | 2.535 (2.176, 2.954) |
| Varicose veins | BMI | 133 | 23.99 | MR Egger | 0.2776 | 1.360 (0.783, 2.362) |
|  |  |  |  | Inverse variance weighted (fixed effects) | 0.0013 | 1.297 (1.107, 1.520) |
|  |  |  |  | Maximum likelihood | 0.0010 | 1.308 (1.114, 1.536) |
| Varicose veins | HC | 145 | 28.52 | MR Egger | 0.0018 | 2.093 (1.327, 3.301) |
|  |  |  |  | Inverse variance weighted | 9.77E-08 | 1.533 (1.310, 1.794) |
|  |  |  |  | Maximum likelihood | 2.66E-10 | 1.548 (1.352, 1.773) |
| Varicose veins | TFP | 73 | 22.24 | MR Egger | 0.4952 | 1.380 (0.549, 3.467) |
|  |  |  |  | Inverse variance weighted (fixed effects) | 4.90E-08 | 1.708 (1.409, 2.070) |
|  |  |  |  | Maximum likelihood | 3.21E-08 | 1.734 (1.427, 2.107) |
| Varicose veins | WC | 92 | 27.99 | MR Egger | 0.0522 | 1.982 (1.003, 3.920) |
|  |  |  |  | Inverse variance weighted (fixed effects) | 9.83E-07 | 1.636 (1.343, 1.992) |
|  |  |  |  | Maximum likelihood | 9.44E-07 | 1.646 (1.349, 2.009) |
| Fasting insulin | BMI | 92 | 21.64 | MR Egger | 0.2450 | −0.068 (−0.184, 0.046) |
|  |  |  |  | Inverse variance weighted | 0.6272 | −0.007 (−0.036, 0.022) |
|  |  |  |  | Maximum likelihood | 0.5753 | −0.007 (−0.033, 0.018) |
| Fasting insulin | HC | 175 | 24.93 | MR Egger | 0.2190 | −0.043 (−0.111, 0.026) |
|  |  |  |  | Inverse variance weighted | 0.0012 | −0.037 (−0.059, −0.014) |
|  |  |  |  | Maximum likelihood | 1.68E-05 | −0.037 (−0.053, −0.020) |
| Fasting insulin | TFP | 114 | 24.21 | MR Egger | 0.6144 | −0.030 (−0.150, 0.088) |
|  |  |  |  | Inverse variance weighted | 0.1027 | −0.027 (−0.059, 0.005) |
|  |  |  |  | Maximum likelihood | 0.0181 | −0.027 (−0.050, −0.005) |
| Fasting insulin | WC | 167 | 23.86 | MR Egger | 0.0924 | −0.066 (−0.142, 0.010) |
|  |  |  |  | Inverse variance weighted | 0.8667 | −0.002 (−0.026, 0.022) |
|  |  |  |  | Maximum likelihood | 0.8354 | −0.002 (−0.021, 0.018) |
| Gout | BMI | 255 | 26.62 | MR Egger | 0.8441 | 0.999 (0.994, 1.005) |
|  |  |  |  | Inverse variance weighted | 0.0477 | 1.002 (1.000, 1.004) |
|  |  |  |  | Maximum likelihood | 0.0179 | 1.002 (1.000, 1.004) |
| Gout | HC | 175 | 25.89 | MR Egger | 0.5172 | 0.997 (0.990, 1.005) |
|  |  |  |  | Inverse variance weighted | 0.1138 | 0.998 (0.996, 1.000) |
|  |  |  |  | Maximum likelihood | 0.0455 | 0.998 (0.996, 1.000) |
| Gout | TFP | 100 | 26.73 | MR Egger | 0.1711 | 0.991 (0.979, 1.004) |
|  |  |  |  | Inverse variance weighted | 0.2133 | 0.998 (0.994, 1.001) |
|  |  |  |  | Maximum likelihood | 0.1070 | 0.998 (0.995, 1.000) |
| Gout | WC | 176 | 24.23 | MR Egger | 0.9520 | 1.000 (0.992, 1.008) |
|  |  |  |  | Inverse variance weighted | 0.4099 | 1.001 (0.999, 1.004) |
|  |  |  |  | Maximum likelihood | 0.3513 | 1.001 (0.999, 1.003) |
| Hyperthyroidism | BMI | 87 | 22.92 | MR Egger | 0.1178 | 2.292 (0.819, 6.415) |
|  |  |  |  | Inverse variance weighted (fixed effects) | 0.0535 | 1.307 (0.996, 1.716) |
|  |  |  |  | Maximum likelihood | 0.0526 | 1.312 (0.997, 1.728) |
| Hyperthyroidism | HC | 163 | 26.54 | MR Egger | 0.0245 | 1.954 (1.096, 3.484) |
|  |  |  |  | Inverse variance weighted (fixed effects) | 0.0178 | 1.248 (1.039, 1.499) |
|  |  |  |  | Maximum likelihood | 0.0162 | 1.254 (1.043, 1.509) |
| Hyperthyroidism | TFP | 81 | 23.75 | MR Egger | 0.9965 | 0.998 (0.335, 2.969) |
|  |  |  |  | Inverse variance weighted (fixed effects) | 0.0118 | 1.448 (1.085, 1.932) |
|  |  |  |  | Maximum likelihood | 0.0110 | 1.459 (1.090, 1.952) |
| Hyperthyroidism | WC | 175 | 24.61 | MR Egger | 0.0437 | 2.061 (1.026, 4.142) |
|  |  |  |  | Inverse variance weighted (fixed effects) | 0.0022 | 1.375 (1.121, 1.686) |
|  |  |  |  | Maximum likelihood | 0.0020 | 1.384 (1.126, 1.701) |
| Hypothyroidism | BMI | 78 | 24.08 | MR Egger | 0.5270 | 0.829 (0.465, 1.478) |
|  |  |  |  | Inverse variance weighted | 0.0164 | 1.215 (1.036, 1.424) |
|  |  |  |  | Maximum likelihood | 0.0019 | 1.219 (1.076, 1.382) |
| Hypothyroidism | HC | 138 | 29.72 | MR Egger | 0.1462 | 1.240 (0.929, 1.656) |
|  |  |  |  | Inverse variance weighted (fixed effects) | 9.36E-10 | 1.310 (1.201, 1.428) |
|  |  |  |  | Maximum likelihood | 1.01E-09 | 1.313 (1.203, 1.434) |
| Hypothyroidism | TFP | 80 | 30.75 | MR Egger | 0.4580 | 0.809 (0.464, 1.411) |
|  |  |  |  | Inverse variance weighted | 0.0008 | 1.319 (1.121, 1.551) |
|  |  |  |  | Maximum likelihood | 5.62E-06 | 1.328 (1.175, 1.501) |
| Hypothyroidism | WC | 147 | 27.67 | MR Egger | 0.1069 | 1.365 (0.937, 1.987) |
|  |  |  |  | Inverse variance weighted | 6.53E-09 | 1.418 (1.260, 1.596) |
|  |  |  |  | Maximum likelihood | 1.13E-12 | 1.428 (1.294, 1.575) |
| Insulin resistance | BMI | 62 | 23.67 | MR Egger | 0.9774 | 0.004 (−0.263, 0.271) |
|  |  |  |  | Inverse variance weighted (fixed effects) | 6.05E-06 | 0.136 (0.078, 0.196) |
|  |  |  |  | Maximum likelihood | 6.11E-06 | 0.139 (0.079, 0.199) |
| Insulin resistance | HC | 84 | 26.37 | MR Egger | 0.0700 | 0.160 (−0.011, 0.330) |
|  |  |  |  | Inverse variance weighted (fixed effects) | 0.0019 | 0.075 (0.028, 0.123) |
|  |  |  |  | Maximum likelihood | 0.0020 | 0.076 (0.028, 0.124) |
| Insulin resistance | TFP | 92 | 22.74 | MR Egger | 0.3334 | −0.132 (−0.397, 0.134) |
|  |  |  |  | Inverse variance weighted | 0.0167 | 0.080 (0.015, 0.146) |
|  |  |  |  | Maximum likelihood | 0.0029 | 0.081 (0.028, 0.135) |
| Insulin resistance | WC | 80 | 21.42 | MR Egger | 0.3607 | −0.137 (−0.429, 0.155) |
|  |  |  |  | Inverse variance weighted (fixed effects) | 0.0209 | 0.070 (0.011, 0.128) |
|  |  |  |  | Maximum likelihood | 0.0211 | 0.070 (0.011, 0.130) |
| Type 1 diabetes | BMI | 118 | 24 | MR Egger | 0.1013 | 12.024 (0.629, 229.937) |
|  |  |  |  | Inverse variance weighted (fixed effects) | 0.1919 | 1.639 (0.780, 3.443) |
|  |  |  |  | Maximum likelihood | 0.1949 | 1.643 (0.776, 3.479) |
| Type 1 diabetes | HC | 160 | 29.62 | MR Egger | 0.5865 | 1.634 (0.280, 9.545) |
|  |  |  |  | Inverse variance weighted (fixed effects) | 0.6578 | 1.143 (0.633, 2.062) |
|  |  |  |  | Maximum likelihood | 0.6592 | 1.143 (0.631, 2.072) |
| Type 1 diabetes | TFP | 112 | 28.81 | MR Egger | 0.2718 | 4.880 (0.293, 81.316) |
|  |  |  |  | Inverse variance weighted (fixed effects) | 0.1742 | 1.710 (0.789, 3.708) |
|  |  |  |  | Maximum likelihood | 0.1736 | 1.718 (0.788, 3.746) |
| Type 1 diabetes | WC | 157 | 27.5 | MR Egger | 0.7816 | 1.349 (0.163, 11.161) |
|  |  |  |  | Inverse variance weighted (fixed effects) | 0.4841 | 1.280 (0.641, 2.559) |
|  |  |  |  | Maximum likelihood | 0.4898 | 1.279 (0.636, 2.570) |
| Serum uric acid | BMI | 28 | 21.46 | MR Egger | 0.8478 | 0.016 (−0.142, 0.173) |
|  |  |  |  | Inverse variance weighted (fixed effects) | 1.93E-15 | 0.158 (0.119, 0.196) |
|  |  |  |  | Maximum likelihood | 2.39E-15 | 0.161 (0.121, 0.201) |
| Serum uric acid | HC | 32 | 23.02 | MR Egger | 0.5706 | −0.057 (−0.250, 0.136) |
|  |  |  |  | Inverse variance weighted | 0.0490 | 0.062 (0.000, 0.125) |
|  |  |  |  | Maximum likelihood | 0.0007 | 0.064 (0.027, 0.100) |
| Serum uric acid | WC | 114 | 21.1 | MR Egger | 0.7774 | 0.023 (−0.132, 0.177) |
|  |  |  |  | Inverse variance weighted | 5.97E-20 | 0.164 (0.128, 0.199) |
|  |  |  |  | Maximum likelihood | 1.51E-46 | 0.169 (0.146, 0.191) |
| Serum uric acid | TFP | 44 | 23.38 | MR Egger | 0.4455 | −0.084 (−0.298, 0.130) |
|  |  |  |  | Inverse variance weighted | 2.72E-05 | 0.108 (0.057, 0.159) |
|  |  |  |  | Maximum likelihood | 5.03E-10 | 0.110 (0.075, 0.144) |
| Pure hypercholesterolaemia | BMI | 97 | 25.05 | MR Egger | 0.8654 | 0.945 (0.492, 1.815) |
|  |  |  |  | Inverse variance weighted (fixed effects) | 0.3561 | 1.111 (0.889, 1.388) |
|  |  |  |  | Maximum likelihood | 0.3518 | 1.113 (0.889, 1.394) |
| Pure hypercholesterolaemia | HC | 73 | 31.89 | MR Egger | 0.3796 | 0.786 (0.461, 1.340) |
|  |  |  |  | Inverse variance weighted (fixed effects) | 0.4083 | 0.913 (0.737, 1.132) |
|  |  |  |  | Maximum likelihood | 0.4117 | 0.913 (0.736, 1.134) |
| Pure hypercholesterolaemia | WC | 59 | 28.76 | MR Egger | 0.7968 | 1.115 (0.488, 2.547) |
|  |  |  |  | Inverse variance weighted (fixed effects) | 0.4700 | 1.110 (0.837, 1.472) |
|  |  |  |  | Maximum likelihood | 0.4624 | 1.113 (0.837, 1.479) |
| Pure hypercholesterolaemia | TFP | 39 | 26.54 | MR Egger | 0.2017 | 0.430 (0.120, 1.535) |
|  |  |  |  | Inverse variance weighted (fixed effects) | 0.3198 | 1.167 (0.861, 1.581) |
|  |  |  |  | Maximum likelihood | 0.3112 | 1.172 (0.862, 1.592) |
| Familial combined hyperlipidemia | BMI | 85 | 22.89 | MR Egger | 0.9055 | 0.949 (0.400, 2.251) |
|  |  |  |  | Inverse variance weighted (fixed effects) | 0.0071 | 1.462 (1.109, 1.928) |
|  |  |  |  | Maximum likelihood | 0.0072 | 1.467 (1.110, 1.940) |
| Familial combined hyperlipidemia | HC | 65 | 25.67 | MR Egger | 0.0599 | 0.375 (0.137, 1.023) |
|  |  |  |  | Inverse variance weighted | 0.0387 | 0.700 (0.499, 0.982) |
|  |  |  |  | Maximum likelihood | 0.0198 | 0.701 (0.520, 0.945) |
| Familial combined hyperlipidemia | WC | 40 | 22.08 | MR Egger | 0.9396 | 1.074 (0.173, 6.646) |
|  |  |  |  | Inverse variance weighted (fixed effects) | 0.0008 | 2.194 (1.383, 3.479) |
|  |  |  |  | Maximum likelihood | 0.0008 | 2.225 (1.396, 3.548) |
| Familial combined hyperlipidemia | TFP | 43 | 25.17 | MR Egger | 0.3024 | 0.458 (0.106, 1.983) |
|  |  |  |  | Inverse variance weighted (fixed effects) | 0.6224 | 0.903 (0.601, 1.357) |
|  |  |  |  | Maximum likelihood | 0.6236 | 0.902 (0.598, 1.361) |
| Mild age-related type 2 diabetes | BMI | 117 | 22.56 | MR Egger | 0.6826 | 0.738 (0.173, 3.147) |
|  |  |  |  | Inverse variance weighted (fixed effects) | 0.8900 | 0.972 (0.646, 1.462) |
|  |  |  |  | Maximum likelihood | 0.8901 | 0.971 (0.643, 1.468) |
| Mild age-related type 2 diabetes | HC | 118 | 23.82 | MR Egger | 0.6022 | 1.405 (0.393, 5.024) |
|  |  |  |  | Inverse variance weighted (fixed effects) | 0.0184 | 0.623 (0.420, 0.923) |
|  |  |  |  | Maximum likelihood | 0.0180 | 0.619 (0.416, 0.921) |
| Mild age-related type 2 diabetes | WC | 71 | 20.68 | MR Egger | 0.7007 | 0.600 (0.045, 8.008) |
|  |  |  |  | Inverse variance weighted (fixed effects) | 0.8951 | 0.959 (0.518, 1.777) |
|  |  |  |  | Maximum likelihood | 0.8947 | 0.959 (0.515, 1.787) |
| Mild age-related type 2 diabetes | TFP | 87 | 23 | MR Egger | 0.1435 | 0.223 (0.031, 1.634) |
|  |  |  |  | Inverse variance weighted (fixed effects) | 0.0654 | 0.620 (0.373, 1.031) |
|  |  |  |  | Maximum likelihood | 0.0726 | 0.625 (0.374, 1.044) |
| Ischaemic stroke | BMI | 216 | 24.69 | MR Egger | 0.8940 | 0.953 (0.471, 1.929) |
|  |  |  |  | Inverse variance weighted | 0.3280 | 1.122 (0.891, 1.414) |
|  |  |  |  | Maximum likelihood | 0.2295 | 1.126 (0.928, 1.367) |
| Ischaemic stroke | HC | 120 | 28.71 | MR Egger | 0.8401 | 0.955 (0.610, 1.495) |
|  |  |  |  | Inverse variance weighted (fixed effects) | 0.6224 | 1.040 (0.890, 1.214) |
|  |  |  |  | Maximum likelihood | 0.6221 | 1.040 (0.890, 1.216) |
| Ischaemic stroke | TFP | 187 | 23.08 | MR Egger | 0.5435 | 0.741 (0.283, 1.940) |
|  |  |  |  | Inverse variance weighted (fixed effects) | 0.7671 | 1.033 (0.834, 1.279) |
|  |  |  |  | Maximum likelihood | 0.7644 | 1.034 (0.833, 1.282) |
| Ischaemic stroke | WC | 213 | 28.23 | MR Egger | 0.5405 | 0.771 (0.337, 1.766) |
|  |  |  |  | Inverse variance weighted | 0.3339 | 1.135 (0.878, 1.468) |
|  |  |  |  | Maximum likelihood | 0.2570 | 1.138 (0.910, 1.424) |
| Stroke | BMI | 112 | 24.73 | MR Egger | 0.5434 | 0.822 (0.437, 1.545) |
|  |  |  |  | Inverse variance weighted | 0.7245 | 1.038 (0.842, 1.280) |
|  |  |  |  | Maximum likelihood | 0.6784 | 1.039 (0.867, 1.246) |
| Stroke | HC | 46 | 28.71 | MR Egger | 0.6145 | 0.894 (0.577, 1.383) |
|  |  |  |  | Inverse variance weighted (fixed effects) | 0.2550 | 1.088 (0.941, 1.259) |
|  |  |  |  | Maximum likelihood | 0.2527 | 1.089 (0.941, 1.262) |
| Stroke | TFP | 38 | 22.96 | MR Egger | 0.2885 | 0.612 (0.248, 1.506) |
|  |  |  |  | Inverse variance weighted (fixed effects) | 0.7718 | 0.971 (0.797, 1.183) |
|  |  |  |  | Maximum likelihood | 0.7720 | 0.971 (0.795, 1.185) |
| Stroke | WC | 91 | 28.26 | MR Egger | 0.4687 | 0.757 (0.357, 1.604) |
|  |  |  |  | Inverse variance weighted | 0.3943 | 1.107 (0.876, 1.399) |
|  |  |  |  | Maximum likelihood | 0.3309 | 1.109 (0.900, 1.365) |
| Nontraumatic intracranial haemorrhage | BMI | 315 | 24.57 | MR Egger | 0.6031 | 0.746 (0.248, 2.244) |
|  |  |  |  | Inverse variance weighted (fixed effects) | 0.0639 | 0.719 (0.507, 1.019) |
|  |  |  |  | Maximum likelihood | 0.0644 | 0.717 (0.504, 1.020) |
| Nontraumatic intracranial haemorrhage | HC | 181 | 28.94 | MR Egger | 0.7028 | 0.845 (0.357, 2.001) |
|  |  |  |  | Inverse variance weighted (fixed effects) | 0.6596 | 1.066 (0.803, 1.415) |
|  |  |  |  | Maximum likelihood | 0.6567 | 1.067 (0.801, 1.421) |
| Nontraumatic intracranial haemorrhage | TFP | 185 | 22.96 | MR Egger | 0.1592 | 0.289 (0.052, 1.599) |
|  |  |  |  | Inverse variance weighted (fixed effects) | 0.3495 | 0.830 (0.561, 1.227) |
|  |  |  |  | Maximum likelihood | 0.3500 | 0.829 (0.558, 1.229) |
| Nontraumatic intracranial haemorrhage | WC | 234 | 28.28 | MR Egger | 0.8394 | 1.154 (0.290, 4.597) |
|  |  |  |  | Inverse variance weighted (fixed effects) | 0.5534 | 0.885 (0.592, 1.324) |
|  |  |  |  | Maximum likelihood | 0.5659 | 0.888 (0.591, 1.333) |
| **Neuromusculoskeletal and mental health disorders** | | | | | | |
| Anxiety disorders | BMI | 130 | 24.59 | MR Egger | 0.5937 | 0.882 (0.558, 1.396) |
|  |  |  |  | Inverse variance weighted | 0.2018 | 1.100 (0.950, 1.273) |
|  |  |  |  | Maximum likelihood | 0.1561 | 1.102 (0.964, 1.260) |
| Anxiety disorders | HC | 93 | 29.44 | MR Egger | 0.3085 | 0.844 (0.610, 1.168) |
|  |  |  |  | Inverse variance weighted (fixed effects) | 0.5120 | 1.038 (0.929, 1.159) |
|  |  |  |  | Maximum likelihood | 0.5049 | 1.039 (0.929, 1.161) |
| Anxiety disorders | TFP | 73 | 22.6 | MR Egger | 0.8709 | 1.058 (0.536, 2.091) |
|  |  |  |  | Inverse variance weighted (fixed effects) | 0.3331 | 1.080 (0.925, 1.260) |
|  |  |  |  | Maximum likelihood | 0.3272 | 1.081 (0.925, 1.264) |
| Anxiety disorders | WC | 154 | 30.37 | MR Egger | 0.2018 | 0.784 (0.540, 1.137) |
|  |  |  |  | Inverse variance weighted (fixed effects) | 0.4772 | 1.054 (0.912, 1.217) |
|  |  |  |  | Maximum likelihood | 0.4746 | 1.054 (0.912, 1.219) |
| Bipolar disorder | BMI | 178 | 29.92 | MR Egger | 0.6225 | 1.137 (0.683, 1.893) |
|  |  |  |  | Inverse variance weighted | 0.1998 | 0.890 (0.744, 1.064) |
|  |  |  |  | Maximum likelihood | 0.0721 | 0.888 (0.780, 1.011) |
| Bipolar disorder | HC | 131 | 22.93 | MR Egger | 0.2113 | 1.229 (0.891, 1.694) |
|  |  |  |  | Inverse variance weighted | 0.7468 | 0.980 (0.869, 1.106) |
|  |  |  |  | Maximum likelihood | 0.6792 | 0.980 (0.891, 1.078) |
| Bipolar disorder | TFP | 79 | 28.4 | MR Egger | 0.5427 | 1.244 (0.619, 2.498) |
|  |  |  |  | Inverse variance weighted | 0.4816 | 1.075 (0.879, 1.313) |
|  |  |  |  | Maximum likelihood | 0.3209 | 1.077 (0.930, 1.248) |
| Bipolar disorder | WC | 119 | 20.73 | MR Egger | 0.6388 | 0.880 (0.516, 1.499) |
|  |  |  |  | Inverse variance weighted | 0.2345 | 0.898 (0.751, 1.073) |
|  |  |  |  | Maximum likelihood | 0.1191 | 0.896 (0.781, 1.029) |
| Fracture of lower leg including ankle | BMI | 65 | 26.29 | MR Egger | 0.1489 | 1.361 (0.897, 2.064) |
|  |  |  |  | Inverse variance weighted (fixed effects) | 8.22E-08 | 1.362 (1.216, 1.525) |
|  |  |  |  | Maximum likelihood | 8.88E-08 | 1.365 (1.218, 1.531) |
| Fracture of lower leg including ankle | HC | 137 | 29.98 | MR Egger | 0.0564 | 1.463 (0.992, 2.158) |
|  |  |  |  | Inverse variance weighted (fixed effects) | 1.80E-07 | 1.357 (1.210, 1.522) |
|  |  |  |  | Maximum likelihood | 1.80E-07 | 1.361 (1.212, 1.528) |
| Fracture of lower leg including ankle | TFP | 90 | 23.41 | MR Egger | 0.3798 | 1.326 (0.708, 2.483) |
|  |  |  |  | Inverse variance weighted | 0.0006 | 1.305 (1.121, 1.519) |
|  |  |  |  | Maximum likelihood | 0.0001 | 1.313 (1.141, 1.511) |
| Fracture of lower leg including ankle | WC | 178 | 28.1 | MR Egger | 0.1669 | 1.417 (0.866, 2.320) |
|  |  |  |  | Inverse variance weighted (fixed effects) | 1.01E-07 | 1.473 (1.277, 1.698) |
|  |  |  |  | Maximum likelihood | 9.40E-08 | 1.480 (1.282, 1.709) |
| Fracture of lumbar spine and pelvis | BMI | 98 | 26.33 | MR Egger | 0.9016 | 0.953 (0.446, 2.039) |
|  |  |  |  | Inverse variance weighted (fixed effects) | 0.7847 | 1.030 (0.835, 1.270) |
|  |  |  |  | Maximum likelihood | 0.7883 | 1.029 (0.833, 1.272) |
| Fracture of lumbar spine and pelvis | HC | 179 | 29.98 | MR Egger | 0.9172 | 1.038 (0.516, 2.086) |
|  |  |  |  | Inverse variance weighted (fixed effects) | 0.7410 | 1.037 (0.837, 1.283) |
|  |  |  |  | Maximum likelihood | 0.7430 | 1.037 (0.836, 1.286) |
| Fracture of lumbar spine and pelvis | TFP | 114 | 23.34 | MR Egger | 0.6229 | 0.760 (0.255, 2.264) |
|  |  |  |  | Inverse variance weighted (fixed effects) | 0.6268 | 1.066 (0.824, 1.378) |
|  |  |  |  | Maximum likelihood | 0.6226 | 1.067 (0.823, 1.384) |
| Fracture of lumbar spine and pelvis | WC | 64 | 30.04 | MR Egger | 0.1711 | 0.531 (0.216, 1.306) |
|  |  |  |  | Inverse variance weighted (fixed effects) | 0.3128 | 1.196 (0.845, 1.693) |
|  |  |  |  | Maximum likelihood | 0.3066 | 1.200 (0.846, 1.704) |
| Malaise and fatigue | BMI | 17 | 24.75 | MR Egger | 0.5138 | 0.785 (0.381, 1.619) |
|  |  |  |  | Inverse variance weighted | 0.0257 | 0.770 (0.612, 0.969) |
|  |  |  |  | Maximum likelihood | 0.0126 | 0.767 (0.623, 0.945) |
| Malaise and fatigue | HC | 23 | 29.37 | MR Egger | 0.5556 | 0.864 (0.533, 1.402) |
|  |  |  |  | Inverse variance weighted (fixed effects) | 0.8790 | 1.013 (0.855, 1.202) |
|  |  |  |  | Maximum likelihood | 0.8778 | 1.014 (0.854, 1.203) |
| Malaise and fatigue | TFP | 190 | 22.6 | MR Egger | 0.9618 | 0.973 (0.314, 3.010) |
|  |  |  |  | Inverse variance weighted (fixed effects) | 0.3580 | 1.119 (0.881, 1.421) |
|  |  |  |  | Maximum likelihood | 0.3530 | 1.122 (0.880, 1.429) |
| Malaise and fatigue | WC | 74 | 28.53 | MR Egger | 0.3246 | 0.666 (0.297, 1.490) |
|  |  |  |  | Inverse variance weighted (fixed effects) | 0.8081 | 0.970 (0.759, 1.240) |
|  |  |  |  | Maximum likelihood | 0.8103 | 0.970 (0.757, 1.243) |
| Sleep disorders | BMI | 117 | 24.1 | MR Egger | 0.8903 | 1.037 (0.616, 1.746) |
|  |  |  |  | Inverse variance weighted | 3.66E-06 | 1.471 (1.249, 1.732) |
|  |  |  |  | Maximum likelihood | 5.00E-08 | 1.487 (1.289, 1.715) |
| Sleep disorders | HC | 87 | 28.85 | MR Egger | 0.6402 | 0.919 (0.645, 1.309) |
|  |  |  |  | Inverse variance weighted (fixed effects) | 0.0227 | 1.144 (1.019, 1.284) |
|  |  |  |  | Maximum likelihood | 0.0217 | 1.147 (1.020, 1.289) |
| Sleep disorders | TFP | 43 | 22.27 | MR Egger | 0.2147 | 1.744 (0.730, 4.165) |
|  |  |  |  | Inverse variance weighted | 0.0086 | 1.300 (1.069, 1.580) |
|  |  |  |  | Maximum likelihood | 0.0016 | 1.308 (1.107, 1.546) |
| Sleep disorders | WC | 105 | 28.27 | MR Egger | 0.1554 | 1.576 (0.846, 2.939) |
|  |  |  |  | Inverse variance weighted | 8.19E-06 | 1.551 (1.279, 1.881) |
|  |  |  |  | Maximum likelihood | 2.31E-07 | 1.562 (1.319, 1.850) |
| Urinary incontinence | BMI | 92 | 25.96 | MR Egger | 0.2289 | 2.634 (0.550, 12.622) |
|  |  |  |  | Inverse variance weighted (fixed effects) | 0.9801 | 1.007 (0.585, 1.734) |
|  |  |  |  | Maximum likelihood | 0.9796 | 1.007 (0.582, 1.741) |
| Urinary incontinence | HC | 146 | 29.37 | MR Egger | 0.0888 | 0.339 (0.098, 1.169) |
|  |  |  |  | Inverse variance weighted | 0.3780 | 0.821 (0.530, 1.273) |
|  |  |  |  | Maximum likelihood | 0.3278 | 0.823 (0.557, 1.216) |
| Urinary incontinence | TFP | 79 | 22.6 | MR Egger | 0.1093 | 7.610 (0.653, 88.658) |
|  |  |  |  | Inverse variance weighted (fixed effects) | 0.4829 | 1.214 (0.706, 2.087) |
|  |  |  |  | Maximum likelihood | 0.4814 | 1.217 (0.704, 2.106) |
| Urinary incontinence | WC | 99 | 28.54 | MR Egger | 0.4048 | 0.430 (0.060, 3.104) |
|  |  |  |  | Inverse variance weighted (fixed effects) | 0.0944 | 1.604 (0.922, 2.789) |
|  |  |  |  | Maximum likelihood | 0.0887 | 1.626 (0.929, 2.846) |
| Alzheimer's disease | BMI | 79 | 22.71 | MR Egger | 0.1775 | 1.332 (0.881, 2.012) |
|  |  |  |  | Inverse variance weighted | 0.3788 | 0.949 (0.844, 1.067) |
|  |  |  |  | Maximum likelihood | 0.3087 | 0.948 (0.854, 1.051) |
| Alzheimer's disease | HC | 161 | 25.46 | MR Egger | 0.2908 | 0.881 (0.696, 1.114) |
|  |  |  |  | Inverse variance weighted | 0.0017 | 0.886 (0.821, 0.956) |
|  |  |  |  | Maximum likelihood | 0.0005 | 0.884 (0.825, 0.948) |
| Alzheimer's disease | TFP | 92 | 27.06 | MR Egger | 0.2166 | 0.781 (0.529, 1.153) |
|  |  |  |  | Inverse variance weighted | 0.0933 | 0.908 (0.812, 1.016) |
|  |  |  |  | Maximum likelihood | 0.0489 | 0.907 (0.824, 1.000) |
| Alzheimer's disease | WC | 169 | 23.63 | MR Egger | 0.0484 | 0.733 (0.540, 0.996) |
|  |  |  |  | Inverse variance weighted | 0.0027 | 0.862 (0.783, 0.950) |
|  |  |  |  | Maximum likelihood | 0.0002 | 0.861 (0.797, 0.931) |
| Depression | BMI | 98 | 24.04 | MR Egger | 0.7703 | 0.938 (0.613, 1.436) |
|  |  |  |  | Inverse variance weighted (fixed effects) | 0.0711 | 1.108 (0.991, 1.239) |
|  |  |  |  | Maximum likelihood | 0.0698 | 1.110 (0.992, 1.243) |
| Depression | HC | 157 | 29.79 | MR Egger | 0.5342 | 0.914 (0.690, 1.211) |
|  |  |  |  | Inverse variance weighted | 0.0663 | 1.092 (0.994, 1.200) |
|  |  |  |  | Maximum likelihood | 0.0312 | 1.094 (1.008, 1.186) |
| Depression | TFP | 90 | 31.03 | MR Egger | 0.2614 | 1.261 (0.844, 1.884) |
|  |  |  |  | Inverse variance weighted (fixed effects) | 0.2806 | 1.065 (0.950, 1.194) |
|  |  |  |  | Maximum likelihood | 0.2781 | 1.066 (0.950, 1.196) |
| Depression | WC | 154 | 27.55 | MR Egger | 0.9373 | 0.987 (0.714, 1.365) |
|  |  |  |  | Inverse variance weighted | 0.0007 | 1.202 (1.082, 1.337) |
|  |  |  |  | Maximum likelihood | 0.0001 | 1.207 (1.097, 1.327) |
| Osteoarthritis | BMI | 224 | 28.83 | MR Egger | 0.0028 | 1.316 (1.101, 1.573) |
|  |  |  |  | Inverse variance weighted | 8.42E-44 | 1.554 (1.460, 1.654) |
|  |  |  |  | Maximum likelihood | 2.10E-51 | 1.558 (1.471, 1.651) |
| Osteoarthritis | HC | 162 | 29.22 | MR Egger | 0.5146 | 1.087 (0.846, 1.397) |
|  |  |  |  | Inverse variance weighted | 3.06E-11 | 1.322 (1.218, 1.436) |
|  |  |  |  | Maximum likelihood | 4.47E-16 | 1.329 (1.241, 1.423) |
| Osteoarthritis | TFP | 97 | 29.92 | MR Egger | 0.2208 | 1.316 (0.850, 2.038) |
|  |  |  |  | Inverse variance weighted | 0.0005 | 1.281 (1.136, 1.444) |
|  |  |  |  | Maximum likelihood | 2.42E-07 | 1.288 (1.170, 1.418) |
| Osteoarthritis | WC | 152 | 27.42 | MR Egger | 0.0093 | 1.407 (1.091, 1.813) |
|  |  |  |  | Inverse variance weighted (fixed effects) | 2.42E-27 | 1.556 (1.436, 1.685) |
|  |  |  |  | Maximum likelihood | 3.10E-27 | 1.565 (1.443, 1.698) |
| Osteoporosis | BMI | 259 | 26.47 | MR Egger | 0.3322 | 0.997 (0.991, 1.003) |
|  |  |  |  | Inverse variance weighted | 0.6383 | 1.000 (0.998, 1.002) |
|  |  |  |  | Maximum likelihood | 0.5766 | 1.000 (0.999, 1.002) |
| Osteoporosis | HC | 236 | 25.62 | MR Egger | 0.2546 | 0.996 (0.990, 1.003) |
|  |  |  |  | Inverse variance weighted | 0.1928 | 0.999 (0.997, 1.001) |
|  |  |  |  | Maximum likelihood | 0.1428 | 0.999 (0.997, 1.000) |
| Osteoporosis | TFP | 193 | 24.09 | MR Egger | 0.7800 | 1.001 (0.992, 1.011) |
|  |  |  |  | Inverse variance weighted | 0.5208 | 1.001 (0.998, 1.003) |
|  |  |  |  | Maximum likelihood | 0.4461 | 1.001 (0.999, 1.003) |
| Osteoporosis | WC | 32 | 24.21 | MR Egger | 0.9957 | 1.000 (0.982, 1.019) |
|  |  |  |  | Inverse variance weighted | 0.4785 | 1.002 (0.996, 1.009) |
|  |  |  |  | Maximum likelihood | 0.3463 | 1.003 (0.997, 1.008) |
| Parkinson's disease | BMI | 93 | 22.29 | MR Egger | 0.3280 | 1.864 (0.539, 6.445) |
|  |  |  |  | Inverse variance weighted (fixed effects) | 0.3469 | 1.171 (0.843, 1.626) |
|  |  |  |  | Maximum likelihood | 0.3368 | 1.176 (0.845, 1.638) |
| Parkinson's disease | HC | 175 | 26.2 | MR Egger | 0.5768 | 1.220 (0.608, 2.446) |
|  |  |  |  | Inverse variance weighted (fixed effects) | 0.7915 | 0.971 (0.783, 1.205) |
|  |  |  |  | Maximum likelihood | 0.7921 | 0.971 (0.781, 1.208) |
| Parkinson's disease | TFP | 100 | 26.56 | MR Egger | 0.8986 | 1.078 (0.341, 3.411) |
|  |  |  |  | Inverse variance weighted (fixed effects) | 0.8843 | 0.977 (0.718, 1.330) |
|  |  |  |  | Maximum likelihood | 0.8844 | 0.977 (0.716, 1.334) |
| Parkinson's disease | WC | 181 | 24.38 | MR Egger | 0.7985 | 0.899 (0.397, 2.036) |
|  |  |  |  | Inverse variance weighted (fixed effects) | 0.5959 | 1.068 (0.836, 1.365) |
|  |  |  |  | Maximum likelihood | 0.5928 | 1.070 (0.835, 1.370) |
| Sleep apnea syndrome | BMI | 62 | 23.83 | MR Egger | 0.0109 | 3.655 (1.389, 9.617) |
|  |  |  |  | Inverse variance weighted | 4.41E-11 | 2.192 (1.735, 2.768) |
|  |  |  |  | Maximum likelihood | 1.39E-15 | 2.239 (1.837, 2.729) |
| Sleep apnea syndrome | HC | 214 | 24.59 | MR Egger | 0.3492 | 1.218 (0.807, 1.838) |
|  |  |  |  | Inverse variance weighted | 6.86E-15 | 1.628 (1.440, 1.840) |
|  |  |  |  | Maximum likelihood | 3.86E-23 | 1.642 (1.488, 1.811) |
| Sleep apnea syndrome | TFP | 169 | 23.55 | MR Egger | 0.1593 | 1.558 (0.843, 2.880) |
|  |  |  |  | Inverse variance weighted | 5.92E-09 | 1.586 (1.358, 1.853) |
|  |  |  |  | Maximum likelihood | 6.29E-14 | 1.605 (1.418, 1.816) |
| Sleep apnea syndrome | WC | 180 | 22.89 | MR Egger | 0.0077 | 2.102 (1.225, 3.609) |
|  |  |  |  | Inverse variance weighted | 3.21E-28 | 2.226 (1.931, 2.567) |
|  |  |  |  | Maximum likelihood | 7.77E-38 | 2.269 (2.002, 2.570) |
| Intervertebral disk disorders | BMI | 216 | 25.57 | MR Egger | 0.7561 | 0.999 (0.994, 1.004) |
|  |  |  |  | Inverse variance weighted (fixed effects) | 0.0625 | 1.001 (1.000, 1.003) |
|  |  |  |  | Maximum likelihood | 0.0626 | 1.001 (1.000, 1.003) |
| Intervertebral disk disorders | HC | 181 | 31.27 | MR Egger | 0.9672 | 1.000 (0.995, 1.004) |
|  |  |  |  | Inverse variance weighted (fixed effects) | 0.5066 | 1.000 (0.999, 1.002) |
|  |  |  |  | Maximum likelihood | 0.5065 | 1.000 (0.999, 1.002) |
| Intervertebral disk disorders | WC | 136 | 27.84 | MR Egger | 0.9851 | 1.000 (0.994, 1.007) |
|  |  |  |  | Inverse variance weighted (fixed effects) | 0.3324 | 1.001 (0.999, 1.003) |
|  |  |  |  | Maximum likelihood | 0.3348 | 1.001 (0.999, 1.003) |
| Intervertebral disk disorders | TFP | 120 | 22.85 | MR Egger | 0.6970 | 0.998 (0.990, 1.006) |
|  |  |  |  | Inverse variance weighted | 0.2275 | 1.001 (0.999, 1.003) |
|  |  |  |  | Maximum likelihood | 0.1700 | 1.001 (1.000, 1.003) |
| Lumbar spine bone mineral density | BMI | 302 | 26.42 | MR Egger | 0.3632 | 0.122 (−0.140, 0.384) |
|  |  |  |  | Inverse variance weighted | 0.4879 | 0.027 (−0.048, 0.102) |
|  |  |  |  | Maximum likelihood | 0.4467 | 0.027 (−0.042, 0.095) |
| Lumbar spine bone mineral density | HC | 217 | 29.48 | MR Egger | 0.6207 | −0.069 (−0.345, 0.206) |
|  |  |  |  | Inverse variance weighted | 0.6764 | −0.019 (−0.106, 0.070) |
|  |  |  |  | Maximum likelihood | 0.6220 | −0.019 (−0.094, 0.056) |
| Lumbar spine bone mineral density | WC | 187 | 27.21 | MR Egger | 0.7067 | 0.073 (−0.308, 0.455) |
|  |  |  |  | Inverse variance weighted | 0.9704 | 0.002 (−0.108, 0.112) |
|  |  |  |  | Maximum likelihood | 0.9657 | 0.002 (−0.094, 0.098) |
| Lumbar spine bone mineral density | TFP | 156 | 22.99 | MR Egger | 0.8225 | 0.042 (−0.322, 0.405) |
|  |  |  |  | Inverse variance weighted | 0.2944 | −0.051 (−0.146, 0.044) |
|  |  |  |  | Maximum likelihood | 0.2448 | −0.051 (−0.137, 0.035) |
| Heel bone mineral density | BMI | 17 | 26.42 | MR Egger | 0.4514 | 0.083 (−0.128, 0.295) |
|  |  |  |  | Inverse variance weighted | 0.5374 | 0.027 (−0.058, 0.112) |
|  |  |  |  | Maximum likelihood | 0.2054 | 0.029 (−0.015, 0.072) |
| Heel bone mineral density | HC | 19 | 20.57 | MR Egger | 0.7013 | 0.109 (−0.437, 0.654) |
|  |  |  |  | Inverse variance weighted | 0.0503 | −0.079 (−0.159, 0.000) |
|  |  |  |  | Maximum likelihood | 0.0037 | −0.078 (−0.131, −0.025) |
| Heel bone mineral density | WC | 16 | 21.75 | MR Egger | 0.2703 | 0.141 (−0.100, 0.383) |
|  |  |  |  | Inverse variance weighted | 0.7214 | 0.018 (−0.082, 0.119) |
|  |  |  |  | Maximum likelihood | 0.4840 | 0.020 (−0.036, 0.075) |
| Heel bone mineral density | TFP | 23 | 22.62 | MR Egger | 0.4291 | 0.086 (−0.123, 0.295) |
|  |  |  |  | Inverse variance weighted | 0.2580 | 0.040 (−0.029, 0.109) |
|  |  |  |  | Maximum likelihood | 0.0804 | 0.042 (−0.005, 0.088) |
| Total body bone mineral density (age over 60) | BMI | 255 | 26.4 | MR Egger | 0.2876 | 0.125 (−0.104, 0.354) |
|  |  |  |  | Inverse variance weighted | 0.0214 | 0.092 (0.014, 0.170) |
|  |  |  |  | Maximum likelihood | 0.0065 | 0.092 (0.026, 0.159) |
| Total body bone mineral density (age over 60) | HC | 234 | 25.26 | MR Egger | 0.2749 | −0.137 (−0.384, 0.109) |
|  |  |  |  | Inverse variance weighted | 0.8900 | −0.006 (−0.088, 0.076) |
|  |  |  |  | Maximum likelihood | 0.8696 | −0.006 (−0.075, 0.063) |
| Total body bone mineral density (age over 60) | WC | 190 | 24.42 | MR Egger | 0.5395 | 0.100 (−0.218, 0.418) |
|  |  |  |  | Inverse variance weighted | 0.5445 | 0.031 (−0.070, 0.134) |
|  |  |  |  | Maximum likelihood | 0.4737 | 0.032 (−0.055, 0.120) |
| Total body bone mineral density (age over 60) | TFP | 192 | 24.28 | MR Egger | 0.1603 | −0.248 (−0.594, 0.097) |
|  |  |  |  | Inverse variance weighted | 0.0992 | −0.080 (−0.174, 0.015) |
|  |  |  |  | Maximum likelihood | 0.0663 | −0.080 (−0.165, 0.005) |
| Total body bone mineral density (age 45–60) | BMI | 257 | 26.62 | MR Egger | 0.2959 | −0.122 (−0.351, 0.107) |
|  |  |  |  | Inverse variance weighted | 0.1383 | 0.059 (−0.019, 0.138) |
|  |  |  |  | Maximum likelihood | 0.1001 | 0.060 (−0.012, 0.132) |
| Total body bone mineral density (age 45–60) | HC | 74 | 24.42 | MR Egger | 0.2902 | −0.250 (−0.707, 0.209) |
|  |  |  |  | Inverse variance weighted | 0.4215 | 0.062 (−0.089, 0.214) |
|  |  |  |  | Maximum likelihood | 0.3518 | 0.064 (−0.070, 0.198) |
| Total body bone mineral density (age 45–60) | WC | 193 | 24.46 | MR Egger | 0.9923 | −0.002 (−0.312, 0.308) |
|  |  |  |  | Inverse variance weighted (fixed effects) | 0.6880 | −0.019 (−0.114, 0.075) |
|  |  |  |  | Maximum likelihood | 0.6883 | −0.019 (−0.114, 0.076) |
| Total body bone mineral density (age 45–60) | TFP | 197 | 24.16 | MR Egger | 0.7571 | 0.062 (−0.330, 0.454) |
|  |  |  |  | Inverse variance weighted | 0.0197 | −0.128 (−0.236, −0.020) |
|  |  |  |  | Maximum likelihood | 0.0063 | −0.129 (−0.221, −0.037) |
| Total body bone mineral density (age 30–45) | BMI | 257 | 26.69 | MR Egger | 0.3318 | 0.156 (−0.158, 0.470) |
|  |  |  |  | Inverse variance weighted | 0.0450 | 0.109 (0.002, 0.216) |
|  |  |  |  | Maximum likelihood | 0.0285 | 0.111 (0.012, 0.210) |
| Total body bone mineral density (age 30–45) | HC | 240 | 25.24 | MR Egger | 0.7026 | −0.066 (−0.406, 0.274) |
|  |  |  |  | Inverse variance weighted | 0.7022 | 0.022 (−0.090, 0.133) |
|  |  |  |  | Maximum likelihood | 0.6749 | 0.022 (−0.081, 0.126) |
| Total body bone mineral density (age 30–45) | WC | 192 | 24.53 | MR Egger | 0.1477 | 0.331 (−0.115, 0.777) |
|  |  |  |  | Inverse variance weighted | 0.8317 | 0.016 (−0.128, 0.159) |
|  |  |  |  | Maximum likelihood | 0.8149 | 0.016 (−0.117, 0.148) |
| Total body bone mineral density (age 30–45) | TFP | 195 | 24.27 | MR Egger | 0.8268 | −0.061 (−0.602, 0.480) |
|  |  |  |  | Inverse variance weighted | 0.4924 | −0.051 (−0.200, 0.096) |
|  |  |  |  | Maximum likelihood | 0.4248 | −0.052 (−0.180, 0.076) |
| Myasthenia gravis | BMI | 227 | 26.74 | MR Egger | 0.3120 | 1.471 (0.697, 3.106) |
|  |  |  |  | Inverse variance weighted (fixed effects) | 0.2753 | 1.151 (0.894, 1.483) |
|  |  |  |  | Maximum likelihood | 0.2706 | 1.154 (0.894, 1.490) |
| Myasthenia gravis | HC | 209 | 25.44 | MR Egger | 0.7074 | 1.181 (0.497, 2.807) |
|  |  |  |  | Inverse variance weighted | 0.0023 | 1.578 (1.176, 2.118) |
|  |  |  |  | Maximum likelihood | 0.0007 | 1.591 (1.218, 2.078) |
| Myasthenia gravis | WC | 165 | 25.02 | MR Egger | 0.2698 | 1.835 (0.627, 5.375) |
|  |  |  |  | Inverse variance weighted (fixed effects) | 0.0075 | 1.587 (1.131, 2.226) |
|  |  |  |  | Maximum likelihood | 0.0079 | 1.588 (1.129, 2.235) |
| Myasthenia gravis | TFP | 55 | 23.69 | MR Egger | 0.0419 | 11.749 (1.160, 119.035) |
|  |  |  |  | Inverse variance weighted (fixed effects) | 0.1280 | 1.568 (0.879, 2.796) |
|  |  |  |  | Maximum likelihood | 0.1259 | 1.579 (0.880, 2.836) |
| Schizophrenia | BMI | 315 | 28.76 | MR Egger | 0.1042 | 0.704 (0.461, 1.074) |
|  |  |  |  | Inverse variance weighted (fixed effects) | 0.0003 | 0.749 (0.640, 0.875) |
|  |  |  |  | Maximum likelihood | 0.0003 | 0.748 (0.639, 0.876) |
| Schizophrenia | HC | 250 | 32.54 | MR Egger | 0.1322 | 0.699 (0.438, 1.113) |
|  |  |  |  | Inverse variance weighted (fixed effects) | 0.0108 | 0.811 (0.691, 0.953) |
|  |  |  |  | Maximum likelihood | 0.0112 | 0.811 (0.689, 0.953) |
| Schizophrenia | WC | 218 | 30.6 | MR Egger | 0.0577 | 0.518 (0.263, 1.018) |
|  |  |  |  | Inverse variance weighted | 0.0448 | 0.789 (0.625, 0.994) |
|  |  |  |  | Maximum likelihood | 0.0209 | 0.788 (0.645, 0.965) |
| Schizophrenia | TFP | 181 | 24.81 | MR Egger | 0.8222 | 0.913 (0.412, 2.022) |
|  |  |  |  | Inverse variance weighted | 0.2837 | 0.891 (0.721, 1.101) |
|  |  |  |  | Maximum likelihood | 0.2430 | 0.891 (0.734, 1.082) |
| **Immunity infection and reproductive disorders** | | | | | | |
| Acute lower respiratory infections | BMI | 133 | 25.38 | MR Egger | 0.9838 | 0.994 (0.561, 1.761) |
|  |  |  |  | Inverse variance weighted (fixed effects) | 0.5503 | 1.060 (0.876, 1.281) |
|  |  |  |  | Maximum likelihood | 0.5493 | 1.060 (0.876, 1.284) |
| Acute lower respiratory infections | HC | 103 | 29.51 | MR Egger | 0.9551 | 0.987 (0.620, 1.571) |
|  |  |  |  | Inverse variance weighted (fixed effects) | 0.1607 | 1.115 (0.958, 1.299) |
|  |  |  |  | Maximum likelihood | 0.1563 | 1.118 (0.958, 1.304) |
| Acute lower respiratory infections | TFP | 79 | 23.25 | MR Egger | 0.6496 | 1.251 (0.478, 3.277) |
|  |  |  |  | Inverse variance weighted (fixed effects) | 0.5949 | 1.060 (0.855, 1.313) |
|  |  |  |  | Maximum likelihood | 0.5923 | 1.061 (0.854, 1.318) |
| Acute lower respiratory infections | WC | 143 | 27.67 | MR Egger | 0.0706 | 1.926 (0.955, 3.885) |
|  |  |  |  | Inverse variance weighted (fixed effects) | 0.0365 | 1.267 (1.015, 1.581) |
|  |  |  |  | Maximum likelihood | 0.0356 | 1.271 (1.016, 1.589) |
| Acute upper respiratory infections | BMI | 146 | 25.57 | MR Egger | 0.6314 | 0.912 (0.628, 1.325) |
|  |  |  |  | Inverse variance weighted (fixed effects) | 0.3987 | 1.050 (0.937, 1.177) |
|  |  |  |  | Maximum likelihood | 0.3933 | 1.051 (0.937, 1.180) |
| Acute upper respiratory infections | HC | 99 | 29.51 | MR Egger | 0.0278 | 1.355 (1.037, 1.772) |
|  |  |  |  | Inverse variance weighted (fixed effects) | 0.0086 | 1.130 (1.031, 1.237) |
|  |  |  |  | Maximum likelihood | 0.0082 | 1.132 (1.032, 1.240) |
| Acute upper respiratory infections | TFP | 88 | 23.25 | MR Egger | 0.0327 | 1.829 (1.063, 3.146) |
|  |  |  |  | Inverse variance weighted (fixed effects) | 0.0264 | 1.156 (1.017, 1.313) |
|  |  |  |  | Maximum likelihood | 0.0255 | 1.158 (1.018, 1.317) |
| Acute upper respiratory infections | WC | 95 | 27.67 | MR Egger | 0.1000 | 1.446 (0.936, 2.232) |
|  |  |  |  | Inverse variance weighted (fixed effects) | 0.0023 | 1.229 (1.076, 1.402) |
|  |  |  |  | Maximum likelihood | 0.0019 | 1.236 (1.081, 1.412) |
| Ectopic pregnancy | BMI | 133 | 23.69 | MR Egger | 0.5655 | 0.739 (0.263, 2.071) |
|  |  |  |  | Inverse variance weighted (fixed effects) | 0.7910 | 1.042 (0.770, 1.409) |
|  |  |  |  | Maximum likelihood | 0.7884 | 1.043 (0.769, 1.415) |
| Ectopic pregnancy | HC | 92 | 29.03 | MR Egger | 0.4276 | 0.746 (0.362, 1.536) |
|  |  |  |  | Inverse variance weighted (fixed effects) | 0.7726 | 0.963 (0.747, 1.242) |
|  |  |  |  | Maximum likelihood | 0.7734 | 0.963 (0.746, 1.244) |
| Ectopic pregnancy | TFP | 149 | 22.64 | MR Egger | 0.4964 | 0.573 (0.116, 2.828) |
|  |  |  |  | Inverse variance weighted (fixed effects) | 0.0253 | 1.506 (1.052, 2.156) |
|  |  |  |  | Maximum likelihood | 0.0269 | 1.505 (1.048, 2.163) |
| Ectopic pregnancy | WC | 161 | 27.69 | MR Egger | 0.9600 | 0.968 (0.274, 3.419) |
|  |  |  |  | Inverse variance weighted (fixed effects) | 0.8062 | 1.047 (0.728, 1.505) |
|  |  |  |  | Maximum likelihood | 0.8076 | 1.047 (0.725, 1.511) |
| Female infertility | BMI | 89 | 24.69 | MR Egger | 0.8582 | 1.070 (0.513, 2.231) |
|  |  |  |  | Inverse variance weighted (fixed effects) | 0.7783 | 1.035 (0.817, 1.311) |
|  |  |  |  | Maximum likelihood | 0.7781 | 1.035 (0.815, 1.314) |
| Female infertility | HC | 150 | 28.96 | MR Egger | 0.6554 | 0.887 (0.523, 1.503) |
|  |  |  |  | Inverse variance weighted (fixed effects) | 0.7432 | 1.032 (0.856, 1.243) |
|  |  |  |  | Maximum likelihood | 0.7427 | 1.032 (0.855, 1.246) |
| Female infertility | TFP | 96 | 22.77 | MR Egger | 0.6209 | 0.729 (0.210, 2.537) |
|  |  |  |  | Inverse variance weighted (fixed effects) | 0.4595 | 0.906 (0.697, 1.178) |
|  |  |  |  | Maximum likelihood | 0.4698 | 0.907 (0.695, 1.182) |
| Female infertility | WC | 172 | 27.69 | MR Egger | 0.4701 | 0.698 (0.265, 1.843) |
|  |  |  |  | Inverse variance weighted | 0.9151 | 0.984 (0.727, 1.331) |
|  |  |  |  | Maximum likelihood | 0.9039 | 0.983 (0.750, 1.289) |
| Infections of the skin and subcutaneous tissue | BMI | 84 | 24.35 | MR Egger | 0.0992 | 1.638 (0.915, 2.931) |
|  |  |  |  | Inverse variance weighted (fixed effects) | 2.42E-06 | 1.549 (1.291, 1.857) |
|  |  |  |  | Maximum likelihood | 1.84E-06 | 1.564 (1.302, 1.880) |
| Infections of the skin and subcutaneous tissue | HC | 129 | 28.42 | MR Egger | 0.2775 | 1.279 (0.822, 1.991) |
|  |  |  |  | Inverse variance weighted (fixed effects) | 0.0006 | 1.291 (1.115, 1.495) |
|  |  |  |  | Maximum likelihood | 0.0006 | 1.295 (1.117, 1.502) |
| Infections of the skin and subcutaneous tissue | TFP | 68 | 24.83 | MR Egger | 0.0342 | 2.379 (1.082, 5.231) |
|  |  |  |  | Inverse variance weighted (fixed effects) | 0.0017 | 1.362 (1.123, 1.653) |
|  |  |  |  | Maximum likelihood | 0.0014 | 1.375 (1.131, 1.672) |
| Infections of the skin and subcutaneous tissue | WC | 214 | 27.45 | MR Egger | 0.0024 | 2.905 (1.486, 5.678) |
|  |  |  |  | Inverse variance weighted (fixed effects) | 4.83E-08 | 1.806 (1.460, 2.232) |
|  |  |  |  | Maximum likelihood | 3.35E-08 | 1.831 (1.477, 2.269) |
| Puerperal sepsis | BMI | 212 | 24.59 | MR Egger | 0.1769 | 2.432 (0.675, 8.764) |
|  |  |  |  | Inverse variance weighted (fixed effects) | 0.0576 | 1.446 (0.988, 2.114) |
|  |  |  |  | Maximum likelihood | 0.0607 | 1.444 (0.984, 2.121) |
| Puerperal sepsis | HC | 105 | 29.03 | MR Egger | 0.9768 | 1.013 (0.433, 2.369) |
|  |  |  |  | Inverse variance weighted (fixed effects) | 0.7139 | 1.058 (0.783, 1.429) |
|  |  |  |  | Maximum likelihood | 0.7099 | 1.059 (0.782, 1.434) |
| Puerperal sepsis | TFP | 133 | 22.77 | MR Egger | 0.1560 | 4.136 (0.593, 28.826) |
|  |  |  |  | Inverse variance weighted (fixed effects) | 0.9730 | 0.993 (0.650, 1.515) |
|  |  |  |  | Maximum likelihood | 0.9732 | 0.993 (0.647, 1.522) |
| Puerperal sepsis | WC | 76 | 27.69 | MR Egger | 0.9082 | 1.086 (0.267, 4.422) |
|  |  |  |  | Inverse variance weighted (fixed effects) | 0.4754 | 0.855 (0.555, 1.316) |
|  |  |  |  | Maximum likelihood | 0.4800 | 0.855 (0.553, 1.321) |
| Polycystic ovary syndrome | BMI | 83 | 24.98 | MR Egger | 0.2968 | 1.901 (0.573, 6.308) |
|  |  |  |  | Inverse variance weighted (fixed effects) | 0.0630 | 1.347 (0.984, 1.845) |
|  |  |  |  | Maximum likelihood | 0.0610 | 1.354 (0.986, 1.859) |
| Polycystic ovary syndrome | HC | 150 | 29.9 | MR Egger | 0.1257 | 1.664 (0.870, 3.183) |
|  |  |  |  | Inverse variance weighted (fixed effects) | 0.2627 | 1.131 (0.912, 1.402) |
|  |  |  |  | Maximum likelihood | 0.2549 | 1.134 (0.913, 1.409) |
| Polycystic ovary syndrome | TFP | 84 | 31.56 | MR Egger | 0.7999 | 1.174 (0.342, 4.030) |
|  |  |  |  | Inverse variance weighted | 0.0917 | 1.355 (0.952, 1.929) |
|  |  |  |  | Maximum likelihood | 0.0562 | 1.361 (0.992, 1.866) |
| Polycystic ovary syndrome | WC | 154 | 27.85 | MR Egger | 0.1566 | 1.751 (0.810, 3.784) |
|  |  |  |  | Inverse variance weighted (fixed effects) | 0.1508 | 1.199 (0.936, 1.535) |
|  |  |  |  | Maximum likelihood | 0.1422 | 1.205 (0.939, 1.546) |
| Preeclampsia | BMI | 78 | 23.57 | MR Egger | 0.7377 | 0.770 (0.167, 3.543) |
|  |  |  |  | Inverse variance weighted (fixed effects) | 0.2951 | 0.817 (0.560, 1.193) |
|  |  |  |  | Maximum likelihood | 0.2984 | 0.817 (0.558, 1.196) |
| Preeclampsia | HC | 173 | 26.2 | MR Egger | 0.0829 | 1.916 (0.923, 3.981) |
|  |  |  |  | Inverse variance weighted (fixed effects) | 0.6679 | 1.052 (0.833, 1.329) |
|  |  |  |  | Maximum likelihood | 0.6691 | 1.053 (0.832, 1.332) |
| Preeclampsia | TFP | 99 | 26.7 | MR Egger | 0.3321 | 1.884 (0.527, 6.733) |
|  |  |  |  | Inverse variance weighted (fixed effects) | 0.5427 | 1.110 (0.794, 1.551) |
|  |  |  |  | Maximum likelihood | 0.5407 | 1.111 (0.792, 1.558) |
| Preeclampsia | WC | 189 | 23.28 | MR Egger | 0.0226 | 3.050 (1.179, 7.892) |
|  |  |  |  | Inverse variance weighted (fixed effects) | 0.0486 | 1.302 (1.002, 1.693) |
|  |  |  |  | Maximum likelihood | 0.0447 | 1.312 (1.006, 1.710) |
| Sporadic miscarriage | BMI | 104 | 21.48 | MR Egger | 0.5581 | 1.106 (0.790, 1.550) |
|  |  |  |  | Inverse variance weighted (fixed effects) | 0.8013 | 1.011 (0.930, 1.099) |
|  |  |  |  | Maximum likelihood | 0.7991 | 1.011 (0.929, 1.100) |
| Sporadic miscarriage | HC | 180 | 25.7 | MR Egger | 0.5572 | 0.944 (0.778, 1.144) |
|  |  |  |  | Inverse variance weighted (fixed effects) | 0.5340 | 1.019 (0.961, 1.079) |
|  |  |  |  | Maximum likelihood | 0.5312 | 1.019 (0.961, 1.080) |
| Sporadic miscarriage | TFP | 105 | 26.22 | MR Egger | 0.4014 | 0.876 (0.645, 1.191) |
|  |  |  |  | Inverse variance weighted (fixed effects) | 0.6692 | 0.982 (0.905, 1.066) |
|  |  |  |  | Maximum likelihood | 0.6725 | 0.982 (0.904, 1.067) |
| Sporadic miscarriage | WC | 179 | 23.87 | MR Egger | 0.0882 | 0.831 (0.672, 1.027) |
|  |  |  |  | Inverse variance weighted (fixed effects) | 0.8214 | 1.008 (0.943, 1.077) |
|  |  |  |  | Maximum likelihood | 0.8199 | 1.008 (0.942, 1.078) |
| Rheumatoid arthritis | BMI | 316 | 28.75 | MR Egger | 0.0419 | 1.568 (1.018, 2.413) |
|  |  |  |  | Inverse variance weighted (fixed effects) | 0.0024 | 1.281 (1.092, 1.502) |
|  |  |  |  | Maximum likelihood | 0.0021 | 1.287 (1.096, 1.512) |
| Rheumatoid arthritis | HC | 251 | 32.54 | MR Egger | 0.0192 | 1.726 (1.096, 2.716) |
|  |  |  |  | Inverse variance weighted (fixed effects) | 5.71E-06 | 1.461 (1.240, 1.721) |
|  |  |  |  | Maximum likelihood | 5.80E-06 | 1.465 (1.242, 1.728) |
| Rheumatoid arthritis | WC | 220 | 30.49 | MR Egger | 0.0868 | 1.685 (0.930, 3.052) |
|  |  |  |  | Inverse variance weighted (fixed effects) | 1.42E-05 | 1.567 (1.280, 1.920) |
|  |  |  |  | Maximum likelihood | 1.27E-05 | 1.578 (1.286, 1.936) |
| Rheumatoid arthritis | TFP | 180 | 24.82 | MR Egger | 0.3925 | 1.386 (0.657, 2.920) |
|  |  |  |  | Inverse variance weighted (fixed effects) | 0.0001 | 1.481 (1.217, 1.803) |
|  |  |  |  | Maximum likelihood | 0.0001 | 1.491 (1.223, 1.819) |
| Systemic lupus erythematosus | BMI | 254 | 26.96 | MR Egger | 0.5287 | 0.725 (0.267, 1.970) |
|  |  |  |  | Inverse variance weighted (fixed effects) | 0.2043 | 1.232 (0.893, 1.701) |
|  |  |  |  | Maximum likelihood | 0.2047 | 1.235 (0.891, 1.710) |
| Systemic lupus erythematosus | HC | 231 | 25.83 | MR Egger | 0.9796 | 0.987 (0.353, 2.754) |
|  |  |  |  | Inverse variance weighted (fixed effects) | 0.1021 | 1.326 (0.945, 1.860) |
|  |  |  |  | Maximum likelihood | 0.1057 | 1.325 (0.942, 1.863) |
| Systemic lupus erythematosus | WC | 186 | 24.91 | MR Egger | 0.9797 | 0.982 (0.243, 3.974) |
|  |  |  |  | Inverse variance weighted (fixed effects) | 0.1735 | 1.346 (0.877, 2.065) |
|  |  |  |  | Maximum likelihood | 0.1760 | 1.347 (0.875, 2.075) |
| Systemic lupus erythematosus | TFP | 193 | 24.82 | MR Egger | 0.9604 | 1.040 (0.225, 4.809) |
|  |  |  |  | Inverse variance weighted (fixed effects) | 0.1171 | 1.392 (0.920, 2.104) |
|  |  |  |  | Maximum likelihood | 0.1185 | 1.394 (0.919, 2.115) |
| Multiple sclerosis | BMI | 315 | 28.8 | MR Egger | 0.5559 | 0.761 (0.308, 1.885) |
|  |  |  |  | Inverse variance weighted (fixed effects) | 0.8239 | 1.037 (0.753, 1.428) |
|  |  |  |  | Maximum likelihood | 0.8244 | 1.037 (0.751, 1.432) |
| Multiple sclerosis | HC | 252 | 32.58 | MR Egger | 0.5401 | 1.331 (0.534, 3.316) |
|  |  |  |  | Inverse variance weighted (fixed effects) | 0.9830 | 1.004 (0.724, 1.392) |
|  |  |  |  | Maximum likelihood | 0.9832 | 1.004 (0.722, 1.395) |
| Multiple sclerosis | WC | 220 | 30.49 | MR Egger | 0.9421 | 0.957 (0.292, 3.140) |
|  |  |  |  | Inverse variance weighted (fixed effects) | 0.2873 | 1.247 (0.830, 1.872) |
|  |  |  |  | Maximum likelihood | 0.2857 | 1.250 (0.830, 1.883) |
| Multiple sclerosis | TFP | 182 | 24.84 | MR Egger | 0.1872 | 0.333 (0.065, 1.696) |
|  |  |  |  | Inverse variance weighted | 0.9717 | 0.992 (0.642, 1.533) |
|  |  |  |  | Maximum likelihood | 0.9688 | 0.992 (0.668, 1.474) |
| Sex hormone binding globulin | BMI | 49 | 25.38 | MR Egger | 0.9591 | −0.005 (−0.190, 0.180) |
|  |  |  |  | Inverse variance weighted | 5.19E-13 | −0.168 (−0.214, −0.122) |
|  |  |  |  | Maximum likelihood | 4.88E-22 | −0.167 (−0.201, −0.134) |
| Sex hormone binding globulin | HC | 111 | 25.09 | MR Egger | 0.5671 | −0.037 (−0.163, 0.089) |
|  |  |  |  | Inverse variance weighted | 7.06E-10 | −0.108 (−0.142, −0.074) |
|  |  |  |  | Maximum likelihood | 2.53E-23 | −0.111 (−0.132, −0.089) |
| Sex hormone binding globulin | TFP | 68 | 26.75 | MR Egger | 0.5207 | −0.066 (−0.267, 0.135) |
|  |  |  |  | Inverse variance weighted | 6.87E-07 | −0.135 (−0.188, −0.081) |
|  |  |  |  | Maximum likelihood | 1.94E-19 | −0.139 (−0.170, −0.110) |
| Sex hormone binding globulin | WC | 93 | 23.95 | MR Egger | 0.0403 | 0.186 (0.011, 0.360) |
|  |  |  |  | Inverse variance weighted | 3.15E-22 | −0.197 (−0.237, −0.158) |
|  |  |  |  | Maximum likelihood | 1.67E-42 | −0.198 (−0.227, −0.170) |
| Systemic sclerosis | BMI | 317 | 28.73 | MR Egger | 0.5400 | 0.437 (0.031, 6.138) |
|  |  |  |  | Inverse variance weighted (fixed effects) | 0.7404 | 0.848 (0.320, 2.250) |
|  |  |  |  | Maximum likelihood | 0.7430 | 0.848 (0.318, 2.266) |
| Systemic sclerosis | HC | 252 | 32.58 | MR Egger | 0.3567 | 0.272 (0.017, 4.318) |
|  |  |  |  | Inverse variance weighted (fixed effects) | 0.0793 | 0.409 (0.150, 1.110) |
|  |  |  |  | Maximum likelihood | 0.0784 | 0.405 (0.148, 1.108) |
| Systemic sclerosis | WC | 220 | 30.49 | MR Egger | 0.5326 | 0.314 (0.008, 11.904) |
|  |  |  |  | Inverse variance weighted (fixed effects) | 0.5182 | 0.664 (0.192, 2.299) |
|  |  |  |  | Maximum likelihood | 0.5169 | 0.661 (0.189, 2.311) |
| Systemic sclerosis | TFP | 182 | 24.84 | MR Egger | 0.1680 | 0.040 (0.000, 3.794) |
|  |  |  |  | Inverse variance weighted (fixed effects) | 0.1813 | 0.442 (0.134, 1.463) |
|  |  |  |  | Maximum likelihood | 0.1781 | 0.436 (0.130, 1.459) |
| Idiopathic thrombocytopenic purpura | BMI | 253 | 27.01 | MR Egger | 0.6449 | 0.794 (0.298, 2.115) |
|  |  |  |  | Inverse variance weighted (fixed effects) | 0.7927 | 1.046 (0.747, 1.465) |
|  |  |  |  | Maximum likelihood | 0.7947 | 1.046 (0.745, 1.469) |
| Idiopathic thrombocytopenic purpura | HC | 231 | 25.83 | MR Egger | 0.8668 | 1.095 (0.379, 3.168) |
|  |  |  |  | Inverse variance weighted (fixed effects) | 0.3817 | 1.170 (0.823, 1.666) |
|  |  |  |  | Maximum likelihood | 0.3805 | 1.173 (0.822, 1.674) |
| Idiopathic thrombocytopenic purpura | WC | 188 | 24.9 | MR Egger | 0.9442 | 0.952 (0.239, 3.786) |
|  |  |  |  | Inverse variance weighted (fixed effects) | 0.2313 | 1.312 (0.841, 2.047) |
|  |  |  |  | Maximum likelihood | 0.2339 | 1.313 (0.839, 2.055) |
| Idiopathic thrombocytopenic purpura | TFP | 192 | 24.7 | MR Egger | 0.4679 | 1.804 (0.368, 8.837) |
|  |  |  |  | Inverse variance weighted (fixed effects) | 0.3377 | 1.236 (0.802, 1.905) |
|  |  |  |  | Maximum likelihood | 0.3380 | 1.238 (0.800, 1.914) |
| Erectile dysfunction | BMI | 256 | 26.68 | MR Egger | 0.7365 | 0.938 (0.648, 1.359) |
|  |  |  |  | Inverse variance weighted (fixed effects) | 0.0106 | 1.181 (1.040, 1.342) |
|  |  |  |  | Maximum likelihood | 0.0096 | 1.185 (1.042, 1.348) |
| Erectile dysfunction | HC | 234 | 25.4 | MR Egger | 0.6138 | 0.900 (0.597, 1.356) |
|  |  |  |  | Inverse variance weighted (fixed effects) | 0.1230 | 1.111 (0.972, 1.269) |
|  |  |  |  | Maximum likelihood | 0.1219 | 1.112 (0.972, 1.273) |
| Erectile dysfunction | WC | 186 | 24.77 | MR Egger | 0.7569 | 1.087 (0.641, 1.845) |
|  |  |  |  | Inverse variance weighted (fixed effects) | 0.0268 | 1.214 (1.022, 1.441) |
|  |  |  |  | Maximum likelihood | 0.0264 | 1.216 (1.023, 1.446) |
| Erectile dysfunction | TFP | 194 | 24.61 | MR Egger | 0.7359 | 0.901 (0.493, 1.648) |
|  |  |  |  | Inverse variance weighted (fixed effects) | 0.1143 | 1.142 (0.969, 1.346) |
|  |  |  |  | Maximum likelihood | 0.1091 | 1.145 (0.970, 1.352) |
| Male infertility | BMI | 317 | 28.73 | MR Egger | 0.0755 | 2.609 (0.909, 7.484) |
|  |  |  |  | Inverse variance weighted (fixed effects) | 0.6002 | 1.110 (0.751, 1.641) |
|  |  |  |  | Maximum likelihood | 0.6056 | 1.109 (0.748, 1.644) |
| Male infertility | HC | 252 | 32.58 | MR Egger | 0.3282 | 1.794 (0.557, 5.775) |
|  |  |  |  | Inverse variance weighted (fixed effects) | 0.3017 | 1.235 (0.827, 1.843) |
|  |  |  |  | Maximum likelihood | 0.3072 | 1.234 (0.824, 1.848) |
| Male infertility | WC | 220 | 30.5 | MR Egger | 0.2755 | 2.252 (0.525, 9.655) |
|  |  |  |  | Inverse variance weighted (fixed effects) | 0.2847 | 1.312 (0.798, 2.158) |
|  |  |  |  | Maximum likelihood | 0.2925 | 1.309 (0.793, 2.161) |
| Male infertility | TFP | 182 | 24.84 | MR Egger | 0.1550 | 4.032 (0.595, 27.328) |
|  |  |  |  | Inverse variance weighted (fixed effects) | 0.7162 | 0.915 (0.566, 1.478) |
|  |  |  |  | Maximum likelihood | 0.7182 | 0.915 (0.563, 1.485) |
| Prostatitis | BMI | 95 | 24.93 | MR Egger | 0.1971 | 0.339 (0.066, 1.733) |
|  |  |  |  | Inverse variance weighted | 0.8782 | 0.958 (0.553, 1.660) |
|  |  |  |  | Maximum likelihood | 0.8620 | 0.959 (0.595, 1.545) |
| Prostatitis | HC | 181 | 29.13 | MR Egger | 0.3547 | 0.632 (0.240, 1.666) |
|  |  |  |  | Inverse variance weighted (fixed effects) | 0.0399 | 0.728 (0.538, 0.985) |
|  |  |  |  | Maximum likelihood | 0.0408 | 0.727 (0.536, 0.987) |
| Prostatitis | WC | 133 | 27.26 | MR Egger | 0.0468 | 0.240 (0.059, 0.967) |
|  |  |  |  | Inverse variance weighted (fixed effects) | 0.5434 | 0.879 (0.579, 1.333) |
|  |  |  |  | Maximum likelihood | 0.5436 | 0.878 (0.576, 1.337) |
| Prostatitis | TFP | 107 | 23.66 | MR Egger | 0.5277 | 0.589 (0.115, 3.025) |
|  |  |  |  | Inverse variance weighted (fixed effects) | 0.4809 | 1.150 (0.779, 1.698) |
|  |  |  |  | Maximum likelihood | 0.4782 | 1.153 (0.778, 1.709) |
| Sepsis | BMI | 97 | 25.71 | MR Egger | 0.9947 | 1.002 (0.572, 1.754) |
|  |  |  |  | Inverse variance weighted (fixed effects) | 2.04E-05 | 1.485 (1.238, 1.781) |
|  |  |  |  | Maximum likelihood | 1.77E-05 | 1.496 (1.245, 1.799) |
| Sepsis | HC | 76 | 32.62 | MR Egger | 0.9886 | 0.997 (0.637, 1.560) |
|  |  |  |  | Inverse variance weighted (fixed effects) | 0.0045 | 1.291 (1.083, 1.539) |
|  |  |  |  | Maximum likelihood | 0.0040 | 1.297 (1.086, 1.549) |
| Sepsis | WC | 62 | 30.52 | MR Egger | 0.8418 | 0.925 (0.433, 1.978) |
|  |  |  |  | Inverse variance weighted | 0.0168 | 1.381 (1.060, 1.800) |
|  |  |  |  | Maximum likelihood | 0.0053 | 1.392 (1.103, 1.756) |
| Sepsis | TFP | 40 | 26.53 | MR Egger | 0.4222 | 0.615 (0.190, 1.990) |
|  |  |  |  | Inverse variance weighted | 0.6524 | 1.077 (0.779, 1.490) |
|  |  |  |  | Maximum likelihood | 0.5444 | 1.080 (0.843, 1.384) |
| Acute laryngitis and tracheitis | BMI | 110 | 23.71 | MR Egger | 0.6590 | 0.730 (0.182, 2.937) |
|  |  |  |  | Inverse variance weighted (fixed effects) | 0.7063 | 0.925 (0.615, 1.389) |
|  |  |  |  | Maximum likelihood | 0.7098 | 0.925 (0.613, 1.396) |
| Acute laryngitis and tracheitis | HC | 105 | 29.66 | MR Egger | 0.6637 | 1.240 (0.472, 3.261) |
|  |  |  |  | Inverse variance weighted (fixed effects) | 0.1059 | 0.744 (0.520, 1.065) |
|  |  |  |  | Maximum likelihood | 0.1042 | 0.741 (0.516, 1.064) |
| Acute laryngitis and tracheitis | WC | 66 | 28.92 | MR Egger | 0.5517 | 1.578 (0.354, 7.039) |
|  |  |  |  | Inverse variance weighted (fixed effects) | 0.8628 | 0.955 (0.566, 1.611) |
|  |  |  |  | Maximum likelihood | 0.8631 | 0.955 (0.564, 1.616) |
| Acute laryngitis and tracheitis | TFP | 46 | 25.76 | MR Egger | 0.1746 | 4.603 (0.526, 40.257) |
|  |  |  |  | Inverse variance weighted (fixed effects) | 0.2150 | 0.713 (0.418, 1.217) |
|  |  |  |  | Maximum likelihood | 0.2136 | 0.710 (0.414, 1.218) |
| Chronic laryngitis and laryngotracheitis | BMI | 112 | 23.62 | MR Egger | 0.3391 | 1.865 (0.523, 6.655) |
|  |  |  |  | Inverse variance weighted (fixed effects) | 0.0568 | 1.485 (0.989, 2.229) |
|  |  |  |  | Maximum likelihood | 0.0600 | 1.482 (0.984, 2.232) |
| Chronic laryngitis and laryngotracheitis | HC | 104 | 29.72 | MR Egger | 0.2112 | 1.875 (0.704, 4.993) |
|  |  |  |  | Inverse variance weighted (fixed effects) | 0.5117 | 1.129 (0.785, 1.623) |
|  |  |  |  | Maximum likelihood | 0.5132 | 1.130 (0.784, 1.628) |
| Chronic laryngitis and laryngotracheitis | WC | 66 | 28.92 | MR Egger | 0.2521 | 2.504 (0.528, 11.875) |
|  |  |  |  | Inverse variance weighted (fixed effects) | 0.5093 | 0.837 (0.494, 1.419) |
|  |  |  |  | Maximum likelihood | 0.5124 | 0.837 (0.491, 1.425) |
| Chronic laryngitis and laryngotracheitis | TFP | 46 | 25.76 | MR Egger | 0.3150 | 3.163 (0.343, 29.147) |
|  |  |  |  | Inverse variance weighted (fixed effects) | 0.5313 | 1.188 (0.693, 2.034) |
|  |  |  |  | Maximum likelihood | 0.5187 | 1.196 (0.694, 2.060) |
| Urinary tract infection | BMI | 77 | 25.08 | MR Egger | 0.8026 | 0.951 (0.642, 1.409) |
|  |  |  |  | Inverse variance weighted (fixed effects) | 0.0095 | 1.182 (1.042, 1.341) |
|  |  |  |  | Maximum likelihood | 0.0090 | 1.185 (1.043, 1.346) |
| Urinary tract infection | HC | 80 | 28.29 | MR Egger | 0.2990 | 0.816 (0.556, 1.195) |
|  |  |  |  | Inverse variance weighted (fixed effects) | 0.0872 | 1.108 (0.985, 1.247) |
|  |  |  |  | Maximum likelihood | 0.0848 | 1.110 (0.986, 1.251) |
| Urinary tract infection | WC | 38 | 25.27 | MR Egger | 0.8384 | 0.920 (0.413, 2.046) |
|  |  |  |  | Inverse variance weighted (fixed effects) | 0.0086 | 1.323 (1.074, 1.630) |
|  |  |  |  | Maximum likelihood | 0.0084 | 1.328 (1.076, 1.640) |
| Urinary tract infection | TFP | 39 | 31.44 | MR Egger | 0.2315 | 0.643 (0.316, 1.310) |
|  |  |  |  | Inverse variance weighted (fixed effects) | 0.0565 | 1.198 (0.995, 1.441) |
|  |  |  |  | Maximum likelihood | 0.0560 | 1.200 (0.995, 1.447) |
| **Respiratory digestive and other disorders** | | | | | | |
| Acne | BMI | 118 | 24.65 | MR Egger | 0.7161 | 1.338 (0.280, 6.405) |
|  |  |  |  | Inverse variance weighted (fixed effects) | 0.2458 | 1.351 (0.813, 2.244) |
|  |  |  |  | Maximum likelihood | 0.2447 | 1.355 (0.812, 2.263) |
| Acne | HC | 98 | 28.96 | MR Egger | 0.2281 | 2.110 (0.630, 7.067) |
|  |  |  |  | Inverse variance weighted (fixed effects) | 0.0892 | 1.415 (0.948, 2.112) |
|  |  |  |  | Maximum likelihood | 0.0865 | 1.424 (0.951, 2.133) |
| Acne | TFP | 69 | 22.77 | MR Egger | 0.5258 | 2.218 (0.192, 25.681) |
|  |  |  |  | Inverse variance weighted (fixed effects) | 0.1671 | 1.487 (0.847, 2.612) |
|  |  |  |  | Maximum likelihood | 0.1665 | 1.493 (0.846, 2.635) |
| Acne | WC | 132 | 27.69 | MR Egger | 0.6033 | 1.630 (0.260, 10.239) |
|  |  |  |  | Inverse variance weighted (fixed effects) | 0.0764 | 1.681 (0.946, 2.985) |
|  |  |  |  | Maximum likelihood | 0.0727 | 1.699 (0.952, 3.033) |
| Cholecystitis | BMI | 131 | 24.06 | MR Egger | 0.2296 | 1.317 (0.842, 2.058) |
|  |  |  |  | Inverse variance weighted (fixed effects) | 1.71E-13 | 1.605 (1.415, 1.820) |
|  |  |  |  | Maximum likelihood | 1.10E-13 | 1.623 (1.428, 1.844) |
| Cholecystitis | HC | 98 | 29.2 | MR Egger | 0.7578 | 1.058 (0.740, 1.512) |
|  |  |  |  | Inverse variance weighted (fixed effects) | 1.61E-09 | 1.462 (1.292, 1.654) |
|  |  |  |  | Maximum likelihood | 1.26E-09 | 1.472 (1.299, 1.668) |
| Cholecystitis | TFP | 76 | 22.22 | MR Egger | 0.0279 | 2.374 (1.113, 5.065) |
|  |  |  |  | Inverse variance weighted | 4.60E-06 | 1.509 (1.266, 1.800) |
|  |  |  |  | Maximum likelihood | 1.45E-07 | 1.525 (1.303, 1.785) |
| Cholecystitis | WC | 153 | 27.19 | MR Egger | 0.0163 | 2.240 (1.171, 4.283) |
|  |  |  |  | Inverse variance weighted | 1.52E-11 | 1.934 (1.597, 2.343) |
|  |  |  |  | Maximum likelihood | 5.53E-17 | 1.973 (1.683, 2.313) |
| Cholelithiasis | BMI | 107 | 24.06 | MR Egger | 0.1963 | 1.355 (0.856, 2.145) |
|  |  |  |  | Inverse variance weighted (fixed effects) | 3.49E-13 | 1.611 (1.416, 1.831) |
|  |  |  |  | Maximum likelihood | 2.11E-13 | 1.629 (1.430, 1.856) |
| Cholelithiasis | HC | 104 | 31.86 | MR Egger | 0.7388 | 1.070 (0.719, 1.593) |
|  |  |  |  | Inverse variance weighted | 2.51E-07 | 1.520 (1.296, 1.782) |
|  |  |  |  | Maximum likelihood | 6.54E-09 | 1.526 (1.323, 1.761) |
| Cholelithiasis | TFP | 74 | 22.22 | MR Egger | 0.0109 | 2.825 (1.292, 6.174) |
|  |  |  |  | Inverse variance weighted | 1.92E-06 | 1.559 (1.298, 1.871) |
|  |  |  |  | Maximum likelihood | 3.48E-08 | 1.572 (1.339, 1.846) |
| Cholelithiasis | WC | 152 | 27.2 | MR Egger | 0.0137 | 2.315 (1.200, 4.469) |
|  |  |  |  | Inverse variance weighted | 6.35E-12 | 1.978 (1.628, 2.402) |
|  |  |  |  | Maximum likelihood | 2.88E-17 | 2.015 (1.713, 2.370) |
| Malignant neoplasm of kidney | BMI | 257 | 23.82 | MR Egger | 0.8841 | 0.878 (0.152, 5.055) |
|  |  |  |  | Inverse variance weighted (fixed effects) | 0.3447 | 0.768 (0.444, 1.328) |
|  |  |  |  | Maximum likelihood | 0.3443 | 0.766 (0.442, 1.330) |
| Malignant neoplasm of kidney | HC | 195 | 28.77 | MR Egger | 0.6285 | 0.711 (0.179, 2.825) |
|  |  |  |  | Inverse variance weighted (fixed effects) | 0.7282 | 0.922 (0.584, 1.456) |
|  |  |  |  | Maximum likelihood | 0.7323 | 0.923 (0.582, 1.463) |
| Malignant neoplasm of kidney | TFP | 220 | 22.92 | MR Egger | 0.6417 | 0.470 (0.020, 11.149) |
|  |  |  |  | Inverse variance weighted (fixed effects) | 0.1768 | 1.559 (0.818, 2.969) |
|  |  |  |  | Maximum likelihood | 0.1774 | 1.566 (0.816, 3.007) |
| Malignant neoplasm of kidney | WC | 231 | 27.98 | MR Egger | 0.5610 | 0.513 (0.054, 4.834) |
|  |  |  |  | Inverse variance weighted (fixed effects) | 0.0849 | 0.561 (0.290, 1.083) |
|  |  |  |  | Maximum likelihood | 0.0845 | 0.557 (0.286, 1.083) |
| Nonalcoholic fatty liver disease | BMI | 227 | 27.04 | MR Egger | 0.8342 | 0.790 (0.088, 7.118) |
|  |  |  |  | Inverse variance weighted | 0.2128 | 1.548 (0.779, 3.076) |
|  |  |  |  | Maximum likelihood | 0.1664 | 1.549 (0.833, 2.881) |
| Nonalcoholic fatty liver disease | HC | 55 | 28.86 | MR Egger | 0.2477 | 0.390 (0.079, 1.914) |
|  |  |  |  | Inverse variance weighted | 0.8650 | 0.957 (0.575, 1.592) |
|  |  |  |  | Maximum likelihood | 0.8538 | 0.957 (0.598, 1.530) |
| Nonalcoholic fatty liver disease | TFP | 220 | 22.92 | MR Egger | 0.3694 | 0.233 (0.010, 5.515) |
|  |  |  |  | Inverse variance weighted | 0.1368 | 1.759 (0.836, 3.702) |
|  |  |  |  | Maximum likelihood | 0.0891 | 1.755 (0.918, 3.357) |
| Nonalcoholic fatty liver disease | WC | 231 | 28.5 | MR Egger | 0.9698 | 0.948 (0.060, 14.893) |
|  |  |  |  | Inverse variance weighted | 0.0310 | 2.420 (1.084, 5.400) |
|  |  |  |  | Maximum likelihood | 0.0109 | 2.421 (1.226, 4.780) |
| Rosacea | BMI | 78 | 24.65 | MR Egger | 0.7267 | 1.335 (0.265, 6.712) |
|  |  |  |  | Inverse variance weighted (fixed effects) | 0.3592 | 0.783 (0.463, 1.322) |
|  |  |  |  | Maximum likelihood | 0.3595 | 0.781 (0.460, 1.325) |
| Rosacea | HC | 39 | 28.96 | MR Egger | 0.1393 | 2.438 (0.753, 7.892) |
|  |  |  |  | Inverse variance weighted (fixed effects) | 0.7207 | 1.078 (0.713, 1.630) |
|  |  |  |  | Maximum likelihood | 0.7171 | 1.080 (0.712, 1.638) |
| Rrosacea | TFP | 40 | 22.77 | MR Egger | 0.8272 | 0.754 (0.060, 9.477) |
|  |  |  |  | Inverse variance weighted (fixed effects) | 0.1162 | 1.594 (0.891, 2.850) |
|  |  |  |  | Maximum likelihood | 0.1178 | 1.597 (0.888, 2.873) |
| Rosacea | WC | 104 | 27.69 | MR Egger | 0.3227 | 2.771 (0.371, 20.679) |
|  |  |  |  | Inverse variance weighted (fixed effects) | 0.5723 | 1.186 (0.656, 2.146) |
|  |  |  |  | Maximum likelihood | 0.5717 | 1.189 (0.653, 2.163) |
| Acute pancreatitis | BMI | 76 | 23.79 | MR Egger | 0.5518 | 0.708 (0.228, 2.196) |
|  |  |  |  | Inverse variance weighted (fixed effects) | 0.0050 | 1.506 (1.131, 2.004) |
|  |  |  |  | Maximum likelihood | 0.0041 | 1.527 (1.143, 2.038) |
| Acute pancreatitis | HC | 204 | 25.8 | MR Egger | 0.3040 | 0.763 (0.456, 1.276) |
|  |  |  |  | Inverse variance weighted (fixed effects) | 0.5207 | 1.055 (0.896, 1.241) |
|  |  |  |  | Maximum likelihood | 0.5221 | 1.055 (0.895, 1.243) |
| Acute pancreatitis | TFP | 149 | 24.52 | MR Egger | 0.7489 | 1.139 (0.515, 2.516) |
|  |  |  |  | Inverse variance weighted (fixed effects) | 0.0057 | 1.348 (1.091, 1.667) |
|  |  |  |  | Maximum likelihood | 0.0053 | 1.356 (1.095, 1.679) |
| Acute pancreatitis | WC | 181 | 24.59 | MR Egger | 0.9673 | 1.014 (0.526, 1.955) |
|  |  |  |  | Inverse variance weighted (fixed effects) | 2.17E-05 | 1.532 (1.258, 1.866) |
|  |  |  |  | Maximum likelihood | 1.85E-05 | 1.545 (1.266, 1.885) |
| Asthma | BMI | 89 | 23.95 | MR Egger | 0.7025 | 1.087 (0.710, 1.663) |
|  |  |  |  | Inverse variance weighted | 4.13E-05 | 1.245 (1.121, 1.382) |
|  |  |  |  | Maximum likelihood | 1.51E-07 | 1.251 (1.151, 1.360) |
| Asthma | HC | 147 | 29.61 | MR Egger | 0.2084 | 1.150 (0.926, 1.429) |
|  |  |  |  | Inverse variance weighted | 1.11E-05 | 1.175 (1.094, 1.263) |
|  |  |  |  | Maximum likelihood | 6.16E-08 | 1.177 (1.109, 1.248) |
| Asthma | TFP | 79 | 31.84 | MR Egger | 0.2899 | 1.235 (0.838, 1.822) |
|  |  |  |  | Inverse variance weighted | 3.60E-06 | 1.298 (1.162, 1.449) |
|  |  |  |  | Maximum likelihood | 1.35E-09 | 1.305 (1.197, 1.423) |
| Asthma | WC | 150 | 27.65 | MR Egger | 0.4252 | 1.117 (0.851, 1.466) |
|  |  |  |  | Inverse variance weighted | 9.34E-09 | 1.301 (1.189, 1.423) |
|  |  |  |  | Maximum likelihood | 6.57E-15 | 1.306 (1.221, 1.397) |
| Breast cancer | BMI | 97 | 22.11 | MR Egger | 0.7362 | 1.100 (0.633, 1.913) |
|  |  |  |  | Inverse variance weighted | 0.6246 | 1.037 (0.897, 1.199) |
|  |  |  |  | Maximum likelihood | 0.5535 | 1.038 (0.918, 1.174) |
| Breast cancer | HC | 172 | 24.22 | MR Egger | 0.8366 | 0.963 (0.672, 1.379) |
|  |  |  |  | Inverse variance weighted | 0.6144 | 1.026 (0.928, 1.134) |
|  |  |  |  | Maximum likelihood | 0.5542 | 1.027 (0.941, 1.120) |
| Breast cancer | TFP | 96 | 24.98 | MR Egger | 0.8755 | 1.047 (0.589, 1.863) |
|  |  |  |  | Inverse variance weighted | 0.7367 | 0.976 (0.846, 1.126) |
|  |  |  |  | Maximum likelihood | 0.6944 | 0.975 (0.860, 1.106) |
| Breast cancer | WC | 176 | 22.64 | MR Egger | 0.6572 | 1.105 (0.711, 1.717) |
|  |  |  |  | Inverse variance weighted | 0.4033 | 0.952 (0.848, 1.069) |
|  |  |  |  | Maximum likelihood | 0.3150 | 0.951 (0.861, 1.049) |
| Chronic obstructive pulmonary disease | BMI | 100 | 22.34 | MR Egger | 0.0594 | 1.700 (0.986, 2.932) |
|  |  |  |  | Inverse variance weighted (fixed effects) | 1.23E-14 | 1.708 (1.491, 1.957) |
|  |  |  |  | Maximum likelihood | 1.19E-14 | 1.725 (1.502, 1.982) |
| Chronic obstructive pulmonary disease | HC | 172 | 25.88 | MR Egger | 0.4028 | 1.150 (0.829, 1.596) |
|  |  |  |  | Inverse variance weighted | 3.10E-07 | 1.314 (1.183, 1.459) |
|  |  |  |  | Maximum likelihood | 2.18E-08 | 1.317 (1.196, 1.451) |
| Chronic obstructive pulmonary disease | TFP | 85 | 27.44 | MR Egger | 0.6364 | 0.858 (0.455, 1.617) |
|  |  |  |  | Inverse variance weighted | 0.0001 | 1.428 (1.188, 1.715) |
|  |  |  |  | Maximum likelihood | 9.50E-07 | 1.441 (1.245, 1.667) |
| Chronic obstructive pulmonary disease | WC | 171 | 24.51 | MR Egger | 0.2565 | 1.260 (0.846, 1.877) |
|  |  |  |  | Inverse variance weighted | 2.08E-19 | 1.773 (1.565, 2.008) |
|  |  |  |  | Maximum likelihood | 1.36E-24 | 1.785 (1.598, 1.995) |
| Colon cancer | BMI | 65 | 24.09 | MR Egger | 0.9722 | 1.000 (0.992, 1.008) |
|  |  |  |  | Inverse variance weighted (fixed effects) | 0.0446 | 1.002 (1.000, 1.003) |
|  |  |  |  | Maximum likelihood | 0.0441 | 1.002 (1.000, 1.003) |
| Colon cancer | HC | 149 | 26.91 | MR Egger | 0.3643 | 0.998 (0.995, 1.002) |
|  |  |  |  | Inverse variance weighted (fixed effects) | 0.8965 | 1.000 (0.999, 1.001) |
|  |  |  |  | Maximum likelihood | 0.8970 | 1.000 (0.999, 1.001) |
| Colon cancer | TFP | 82 | 28.8 | MR Egger | 0.0660 | 0.995 (0.990, 1.000) |
|  |  |  |  | Inverse variance weighted (fixed effects) | 0.2152 | 0.999 (0.998, 1.001) |
|  |  |  |  | Maximum likelihood | 0.2153 | 0.999 (0.998, 1.001) |
| Colon cancer | WC | 137 | 25.63 | MR Egger | 0.1843 | 0.997 (0.993, 1.001) |
|  |  |  |  | Inverse variance weighted (fixed effects) | 0.1556 | 1.001 (1.000, 1.002) |
|  |  |  |  | Maximum likelihood | 0.1535 | 1.001 (1.000, 1.002) |
| Endometrial cancer | BMI | 103 | 22.22 | MR Egger | 0.7277 | 0.783 (0.198, 3.094) |
|  |  |  |  | Inverse variance weighted | 0.0885 | 1.367 (0.954, 1.959) |
|  |  |  |  | Maximum likelihood | 0.0387 | 1.378 (1.017, 1.867) |
| Endometrial cancer | HC | 178 | 26.25 | MR Egger | 0.1965 | 1.608 (0.784, 3.296) |
|  |  |  |  | Inverse variance weighted (fixed effects) | 0.0001 | 1.512 (1.227, 1.863) |
|  |  |  |  | Maximum likelihood | 0.0001 | 1.526 (1.236, 1.884) |
| Endometrial cancer | TFP | 105 | 26.37 | MR Egger | 0.3943 | 1.645 (0.526, 5.149) |
|  |  |  |  | Inverse variance weighted (fixed effects) | 0.0089 | 1.479 (1.103, 1.984) |
|  |  |  |  | Maximum likelihood | 0.0079 | 1.495 (1.111, 2.011) |
| Endometrial cancer | WC | 181 | 24.38 | MR Egger | 0.0313 | 2.513 (1.094, 5.774) |
|  |  |  |  | Inverse variance weighted | 0.0076 | 1.423 (1.098, 1.844) |
|  |  |  |  | Maximum likelihood | 0.0034 | 1.428 (1.125, 1.814) |
| Esophageal cancer | BMI | 98 | 22.58 | MR Egger | 0.1404 | 2.875 (0.715, 11.570) |
|  |  |  |  | Inverse variance weighted (fixed effects) | 0.5063 | 1.135 (0.782, 1.646) |
|  |  |  |  | Maximum likelihood | 0.5077 | 1.135 (0.780, 1.653) |
| Esophageal cancer | HC | 179 | 26.13 | MR Egger | 0.9885 | 0.994 (0.412, 2.395) |
|  |  |  |  | Inverse variance weighted (fixed effects) | 0.6381 | 1.064 (0.823, 1.375) |
|  |  |  |  | Maximum likelihood | 0.6362 | 1.065 (0.822, 1.379) |
| Esophageal cancer | TFP | 100 | 26.6 | MR Egger | 0.5814 | 1.448 (0.390, 5.380) |
|  |  |  |  | Inverse variance weighted (fixed effects) | 0.3434 | 1.195 (0.826, 1.729) |
|  |  |  |  | Maximum likelihood | 0.3402 | 1.198 (0.826, 1.738) |
| Esophageal cancer | WC | 179 | 24.47 | MR Egger | 0.9422 | 0.965 (0.371, 2.512) |
|  |  |  |  | Inverse variance weighted (fixed effects) | 0.2357 | 1.193 (0.891, 1.597) |
|  |  |  |  | Maximum likelihood | 0.2357 | 1.195 (0.890, 1.604) |
| Gastroesophageal reflux disease | BMI | 32 | 20.23 | MR Egger | 0.8181 | 1.083 (0.553, 2.121) |
|  |  |  |  | Inverse variance weighted | 3.69E-12 | 1.569 (1.382, 1.781) |
|  |  |  |  | Maximum likelihood | 4.38E-17 | 1.593 (1.429, 1.775) |
| Gastroesophageal reflux disease | HC | 64 | 26.3 | MR Egger | 0.7204 | 1.050 (0.804, 1.372) |
|  |  |  |  | Inverse variance weighted | 1.44E-09 | 1.295 (1.191, 1.409) |
|  |  |  |  | Maximum likelihood | 1.02E-15 | 1.300 (1.219, 1.386) |
| Gastroesophageal reflux disease | TFP | 50 | 24.07 | MR Egger | 0.7301 | 1.064 (0.750, 1.508) |
|  |  |  |  | Inverse variance weighted | 1.46E-14 | 1.472 (1.334, 1.625) |
|  |  |  |  | Maximum likelihood | 9.50E-21 | 1.481 (1.364, 1.608) |
| Gastroesophageal reflux disease | WC | 46 | 20.66 | MR Egger | 0.2849 | 1.403 (0.760, 2.588) |
|  |  |  |  | Inverse variance weighted | 1.66E-12 | 1.547 (1.371, 1.746) |
|  |  |  |  | Maximum likelihood | 7.44E-19 | 1.562 (1.415, 1.723) |
| Residual haemorrhoidal skin tags | BMI | 84 | 24.01 | MR Egger | 0.7682 | 1.001 (0.994, 1.008) |
|  |  |  |  | Inverse variance weighted (fixed effects) | 0.1618 | 1.001 (1.000, 1.002) |
|  |  |  |  | Maximum likelihood | 0.1629 | 1.001 (1.000, 1.002) |
| Residual haemorrhoidal skin tags | HC | 137 | 28.05 | MR Egger | 0.7550 | 0.999 (0.996, 1.003) |
|  |  |  |  | Inverse variance weighted (fixed effects) | 0.5833 | 1.000 (0.999, 1.001) |
|  |  |  |  | Maximum likelihood | 0.5868 | 1.000 (0.999, 1.001) |
| Residual haemorrhoidal skin tags | TFP | 84 | 28.54 | MR Egger | 0.2655 | 0.997 (0.992, 1.002) |
|  |  |  |  | Inverse variance weighted (fixed effects) | 0.8612 | 1.000 (0.998, 1.001) |
|  |  |  |  | Maximum likelihood | 0.8612 | 1.000 (0.998, 1.001) |
| Residual haemorrhoidal skin tags | WC | 129 | 26.17 | MR Egger | 0.3974 | 0.998 (0.994, 1.002) |
|  |  |  |  | Inverse variance weighted (fixed effects) | 0.2704 | 1.001 (0.999, 1.002) |
|  |  |  |  | Maximum likelihood | 0.2722 | 1.001 (0.999, 1.002) |
| Inguinal or femoral hernia bilateral | BMI | 262 | 27.28 | MR Egger | 0.0954 | 0.430 (0.160, 1.155) |
|  |  |  |  | Inverse variance weighted | 0.0001 | 0.515 (0.372, 0.713) |
|  |  |  |  | Maximum likelihood | 1.17E-05 | 0.509 (0.377, 0.689) |
| Inguinal or femoral hernia bilateral | HC | 213 | 29.88 | MR Egger | 0.6164 | 0.767 (0.272, 2.163) |
|  |  |  |  | Inverse variance weighted (fixed effects) | 0.0005 | 0.581 (0.427, 0.790) |
|  |  |  |  | Maximum likelihood | 0.0006 | 0.579 (0.425, 0.790) |
| Inguinal or femoral hernia bilateral | WC | 185 | 29.15 | MR Egger | 0.0908 | 0.299 (0.074, 1.203) |
|  |  |  |  | Inverse variance weighted | 3.25E-06 | 0.378 (0.251, 0.569) |
|  |  |  |  | Maximum likelihood | 4.77E-07 | 0.378 (0.259, 0.552) |
| Inguinal or femoral hernia bilateral | TFP | 145 | 23.6 | MR Egger | 0.8236 | 1.207 (0.231, 6.315) |
|  |  |  |  | Inverse variance weighted | 0.0015 | 0.516 (0.343, 0.776) |
|  |  |  |  | Maximum likelihood | 0.0005 | 0.516 (0.355, 0.750) |
| Crohn's disease | BMI | 214 | 28.4 | MR Egger | 0.1411 | 1.348 (0.907, 2.004) |
|  |  |  |  | Inverse variance weighted | 0.0156 | 1.183 (1.032, 1.355) |
|  |  |  |  | Maximum likelihood | 0.0044 | 1.185 (1.054, 1.332) |
| Crohn's disease | HC | 192 | 28.18 | MR Egger | 0.9619 | 0.990 (0.654, 1.498) |
|  |  |  |  | Inverse variance weighted | 0.0267 | 1.175 (1.019, 1.356) |
|  |  |  |  | Maximum likelihood | 0.0086 | 1.181 (1.043, 1.337) |
| Crohn's disease | WC | 148 | 27.75 | MR Egger | 0.1290 | 1.532 (0.886, 2.647) |
|  |  |  |  | Inverse variance weighted | 0.0355 | 1.223 (1.014, 1.476) |
|  |  |  |  | Maximum likelihood | 0.0126 | 1.227 (1.045, 1.440) |
| Crohn's disease | TFP | 153 | 27.2 | MR Egger | 0.5861 | 1.214 (0.605, 2.434) |
|  |  |  |  | Inverse variance weighted | 0.0201 | 1.254 (1.036, 1.518) |
|  |  |  |  | Maximum likelihood | 0.0032 | 1.260 (1.081, 1.469) |
| Ulcerative colitis | BMI | 316 | 28.75 | MR Egger | 0.1392 | 0.708 (0.449, 1.118) |
|  |  |  |  | Inverse variance weighted (fixed effects) | 0.5669 | 0.954 (0.813, 1.120) |
|  |  |  |  | Maximum likelihood | 0.5615 | 0.953 (0.811, 1.121) |
| Ulcerative colitis | HC | 250 | 32.49 | MR Egger | 0.7024 | 0.915 (0.580, 1.444) |
|  |  |  |  | Inverse variance weighted (fixed effects) | 0.9553 | 1.005 (0.852, 1.185) |
|  |  |  |  | Maximum likelihood | 0.9553 | 1.005 (0.851, 1.186) |
| Ulcerative colitis | WC | 218 | 30.58 | MR Egger | 0.4446 | 0.792 (0.436, 1.438) |
|  |  |  |  | Inverse variance weighted (fixed effects) | 0.8123 | 1.025 (0.835, 1.258) |
|  |  |  |  | Maximum likelihood | 0.8127 | 1.025 (0.834, 1.260) |
| Ulcerative colitis | TFP | 180 | 24.85 | MR Egger | 0.6591 | 1.181 (0.565, 2.470) |
|  |  |  |  | Inverse variance weighted (fixed effects) | 0.0355 | 1.236 (1.015, 1.506) |
|  |  |  |  | Maximum likelihood | 0.0345 | 1.240 (1.016, 1.513) |
| Irritable bowel syndrome | BMI | 254 | 26.46 | MR Egger | 0.7052 | 1.032 (0.876, 1.216) |
|  |  |  |  | Inverse variance weighted | 0.0363 | 1.062 (1.004, 1.124) |
|  |  |  |  | Maximum likelihood | 0.0093 | 1.063 (1.015, 1.112) |
| Irritable bowel syndrome | HC | 233 | 25.41 | MR Egger | 0.5871 | 0.954 (0.803, 1.132) |
|  |  |  |  | Inverse variance weighted | 0.9732 | 1.001 (0.945, 1.060) |
|  |  |  |  | Maximum likelihood | 0.9675 | 1.001 (0.954, 1.050) |
| Irritable bowel syndrome | WC | 185 | 24.51 | MR Egger | 0.8192 | 0.974 (0.779, 1.218) |
|  |  |  |  | Inverse variance weighted | 0.0418 | 1.079 (1.003, 1.160) |
|  |  |  |  | Maximum likelihood | 0.0143 | 1.080 (1.015, 1.148) |
| Irritable bowel syndrome | TFP | 191 | 24.36 | MR Egger | 0.5266 | 0.918 (0.705, 1.195) |
|  |  |  |  | Inverse variance weighted | 0.0315 | 1.084 (1.007, 1.166) |
|  |  |  |  | Maximum likelihood | 0.0072 | 1.084 (1.022, 1.150) |
| Malignant neoplasm of prostate | BMI | 212 | 25.15 | MR Egger | 0.7418 | 1.124 (0.561, 2.250) |
|  |  |  |  | Inverse variance weighted (fixed effects) | 0.8760 | 0.984 (0.806, 1.202) |
|  |  |  |  | Maximum likelihood | 0.8749 | 0.984 (0.804, 1.204) |
| Malignant neoplasm of prostate | HC | 181 | 28.93 | MR Egger | 0.5569 | 0.835 (0.457, 1.524) |
|  |  |  |  | Inverse variance weighted (fixed effects) | 0.0267 | 0.807 (0.667, 0.976) |
|  |  |  |  | Maximum likelihood | 0.0282 | 0.807 (0.666, 0.977) |
| Malignant neoplasm of prostate | WC | 132 | 27.28 | MR Egger | 0.9122 | 0.949 (0.376, 2.397) |
|  |  |  |  | Inverse variance weighted (fixed effects) | 0.2162 | 0.847 (0.651, 1.102) |
|  |  |  |  | Maximum likelihood | 0.2218 | 0.847 (0.649, 1.105) |
| Malignant neoplasm of prostate | TFP | 105 | 23.63 | MR Egger | 0.9970 | 0.998 (0.368, 2.705) |
|  |  |  |  | Inverse variance weighted (fixed effects) | 0.4868 | 1.092 (0.853, 1.398) |
|  |  |  |  | Maximum likelihood | 0.4827 | 1.094 (0.852, 1.404) |
| Barrett's esophagus | BMI | 78 | 24.9 | MR Egger | 0.1397 | 1.919 (0.815, 4.517) |
|  |  |  |  | Inverse variance weighted | 0.0106 | 1.447 (1.090, 1.921) |
|  |  |  |  | Maximum likelihood | 0.0017 | 1.464 (1.154, 1.857) |
| Barrett's esophagus | HC | 80 | 28.2 | MR Egger | 0.7417 | 1.144 (0.516, 2.533) |
|  |  |  |  | Inverse variance weighted | 0.5073 | 0.913 (0.698, 1.195) |
|  |  |  |  | Maximum likelihood | 0.4247 | 0.913 (0.729, 1.142) |
| Barrett's esophagus | WC | 37 | 25.41 | MR Egger | 0.5330 | 1.700 (0.326, 8.876) |
|  |  |  |  | Inverse variance weighted (fixed effects) | 0.0538 | 1.481 (0.994, 2.208) |
|  |  |  |  | Maximum likelihood | 0.0515 | 1.493 (0.997, 2.236) |
| Barrett's esophagus | TFP | 39 | 31.44 | MR Egger | 0.1563 | 2.921 (0.684, 12.477) |
|  |  |  |  | Inverse variance weighted (fixed effects) | 0.0207 | 1.510 (1.065, 2.140) |
|  |  |  |  | Maximum likelihood | 0.0178 | 1.532 (1.077, 2.182) |
| Lung adenocarcinoma | BMI | 79 | 23.34 | MR Egger | 0.7947 | 1.093 (0.560, 2.133) |
|  |  |  |  | Inverse variance weighted (fixed effects) | 0.0315 | 1.244 (1.020, 1.519) |
|  |  |  |  | Maximum likelihood | 0.0301 | 1.250 (1.022, 1.529) |
| Lung adenocarcinoma | HC | 88 | 24.82 | MR Egger | 0.6982 | 1.130 (0.610, 2.095) |
|  |  |  |  | Inverse variance weighted (fixed effects) | 0.3186 | 1.097 (0.914, 1.316) |
|  |  |  |  | Maximum likelihood | 0.3120 | 1.100 (0.915, 1.322) |
| Lung adenocarcinoma | WC | 40 | 22.74 | MR Egger | 0.4424 | 0.606 (0.172, 2.144) |
|  |  |  |  | Inverse variance weighted (fixed effects) | 0.4041 | 1.146 (0.832, 1.578) |
|  |  |  |  | Maximum likelihood | 0.4003 | 1.149 (0.832, 1.587) |
| Lung adenocarcinoma | TFP | 49 | 24.68 | MR Egger | 0.4098 | 0.599 (0.179, 2.003) |
|  |  |  |  | Inverse variance weighted | 0.7605 | 1.053 (0.755, 1.469) |
|  |  |  |  | Maximum likelihood | 0.6986 | 1.055 (0.803, 1.387) |
| Colon adenocarcinoma | BMI | 112 | 23.62 | MR Egger | 0.5467 | 1.627 (0.336, 7.881) |
|  |  |  |  | Inverse variance weighted (fixed effects) | 0.7941 | 0.935 (0.565, 1.549) |
|  |  |  |  | Maximum likelihood | 0.7914 | 0.934 (0.561, 1.553) |
| Colon adenocarcinoma | HC | 104 | 29.79 | MR Egger | 0.6378 | 0.715 (0.178, 2.874) |
|  |  |  |  | Inverse variance weighted | 0.2668 | 1.338 (0.800, 2.236) |
|  |  |  |  | Maximum likelihood | 0.2051 | 1.342 (0.851, 2.114) |
| Colon adenocarcinoma | WC | 66 | 28.93 | MR Egger | 0.4280 | 2.247 (0.307, 16.416) |
|  |  |  |  | Inverse variance weighted (fixed effects) | 0.9074 | 0.962 (0.500, 1.852) |
|  |  |  |  | Maximum likelihood | 0.9079 | 0.962 (0.497, 1.863) |
| Colon adenocarcinoma | TFP | 46 | 25.76 | MR Egger | 0.8180 | 1.397 (0.082, 23.659) |
|  |  |  |  | Inverse variance weighted (fixed effects) | 0.8306 | 0.930 (0.477, 1.813) |
|  |  |  |  | Maximum likelihood | 0.8327 | 0.930 (0.474, 1.826) |
| Pulmonary fibrosis | BMI | 82 | 23.87 | MR Egger | 0.7285 | 0.786 (0.203, 3.046) |
|  |  |  |  | Inverse variance weighted (fixed effects) | 0.2662 | 1.277 (0.830, 1.965) |
|  |  |  |  | Maximum likelihood | 0.2599 | 1.284 (0.831, 1.983) |
| Pulmonary fibrosis | HC | 92 | 24.71 | MR Egger | 0.2280 | 2.087 (0.636, 6.851) |
|  |  |  |  | Inverse variance weighted (fixed effects) | 0.0779 | 1.420 (0.962, 2.096) |
|  |  |  |  | Maximum likelihood | 0.0760 | 1.427 (0.964, 2.113) |
| Pulmonary fibrosis | WC | 43 | 22.33 | MR Egger | 0.8965 | 0.840 (0.062, 11.363) |
|  |  |  |  | Inverse variance weighted (fixed effects) | 0.0073 | 2.501 (1.280, 4.887) |
|  |  |  |  | Maximum likelihood | 0.0069 | 2.534 (1.291, 4.975) |
| Pulmonary fibrosis | TFP | 49 | 25.03 | MR Egger | 0.7661 | 1.443 (0.131, 15.917) |
|  |  |  |  | Inverse variance weighted (fixed effects) | 0.0033 | 2.420 (1.343, 4.361) |
|  |  |  |  | Maximum likelihood | 0.0032 | 2.447 (1.349, 4.437) |

Note: Effect size is presented as odds ratio (OR) for binary outcomes and as beta coefficient (β) for continuous outcomes (fast insulin, insulin resistance, insulin receptor protein, serum uric acid, sex hormone binding globulin, and bone mineral density). All effect sizes correspond to a 1‑standard deviation (SD) increase in the exposure.

Abbreviations: CMDs, cardiovascular and metabolic disorders; BMI, body mass index; HC, hip circumference; TFP, total fat percentage; WC, waist circumference.
