# Supplementary material for: Unraveling the causal web of 4 adiposity indices and 92 multi-system outcomes: A body-wide Mendelian randomization study
Source: Medicine (Baltimore). 2026 May 22;105(21):e48986. doi: 10.1097/MD.0000000000048986 (PMC13201005; doi:10.1097/MD.0000000000048986)
Supplement: Supplementary file 6 [file medi-105-e48986-s006.docx]

Table S6. Sensitivity analyses of two-sample Mendelian randomization.

| **Outcome** | **Exposure** | **Pleiotropy test*** | **Heterogeneity test** ^a^ | **Heterogeneity test** ^b^ | **Outliers^#^** |
| --- | --- | --- | --- | --- | --- |
| Acne | BMI | 0.9901 | 0.4715 | 0.4977 | NA |
| Acne | HC | 0.4897 | 0.1166 | 0.1223 | NA |
| Acne | TFP | 0.7434 | 0.7523 | 0.7751 | NA |
| Acne | WC | 0.9727 | 0.6701 | 0.6961 | NA |
| Acute lower respiratory infections | BMI | 0.8174 | 0.5565 | 0.5819 | NA |
| Acute lower respiratory infections | HC | 0.5824 | 0.1363 | 0.1455 | NA |
| Acute lower respiratory infections | TFP | 0.7291 | 0.2387 | 0.2623 | NA |
| Acute lower respiratory infections | WC | 0.2209 | 0.7795 | 0.7651 | NA |
| Acute upper respiratory infections | BMI | 0.4349 | 0.0904 | 0.0940 | NA |
| Acute upper respiratory infections | HC | 0.1571 | 0.3009 | 0.2782 | NA |
| Acute upper respiratory infections | TFP | 0.0926 | 0.9084 | 0.8705 | NA |
| Acute upper respiratory infections | WC | 0.4415 | 0.2937 | 0.3037 | NA |
| Anxiety disorders | BMI | 0.3226 | 0.0484 | 0.0479 | NA |
| Anxiety disorders | HC | 0.1852 | 0.2558 | 0.2404 | NA |
| Anxiety disorders | TFP | 0.9534 | 0.6526 | 0.6821 | NA |
| Anxiety disorders | WC | 0.0937 | 0.9190 | 0.8928 | NA |
| Atrial fibrillation and flutter | BMI | 0.2546 | 0.0029 | 0.0026 | NO |
| Atrial fibrillation and flutter | HC | 0.1379 | 0.0000 | 0.0000 | NA |
| Atrial fibrillation and flutter | TFP | 0.5263 | 0.0213 | 0.0234 | NA |
| Atrial fibrillation and flutter | WC | 0.5915 | 0.2699 | 0.2875 | NA |
| Bipolar disorder | BMI | 0.3167 | 0.0000 | 0.0000 | NA |
| Bipolar disorder | HC | 0.1403 | 0.0000 | 0.0000 | NA |
| Bipolar disorder | TFP | 0.6698 | 0.0000 | 0.0000 | NA |
| Bipolar disorder | WC | 0.9378 | 0.0000 | 0.0000 | NA |
| Cerebral atherosclerosis | BMI | 0.3801 | 0.7608 | 0.7644 | NA |
| Cerebral atherosclerosis | HC | 0.6520 | 0.4449 | 0.4633 | NA |
| Cerebral atherosclerosis | TFP | 0.2722 | 0.0484 | 0.0455 | NA |
| Cerebral atherosclerosis | WC | 0.3469 | 0.2490 | 0.2506 | NA |
| Cholecystitis | BMI | 0.3640 | 0.1614 | 0.1631 | NA |
| Cholecystitis | HC | 0.0597 | 0.3230 | 0.2656 | NA |
| Cholecystitis | TFP | 0.2318 | 0.0370 | 0.0334 | NA |
| Cholecystitis | WC | 0.6436 | 0.0003 | 0.0003 | NA |
| Cholelithiasis | BMI | 0.4419 | 0.1303 | 0.1346 | NA |
| Cholelithiasis | HC | 0.0632 | 0.0594 | 0.0388 | NA |
| Cholelithiasis | TFP | 0.1291 | 0.0261 | 0.0196 | NA |
| Cholelithiasis | WC | 0.6235 | 0.0004 | 0.0005 | NA |
| Coronary atherosclerosis | BMI | 0.4363 | 0.0063 | 0.0066 | NA |
| Coronary atherosclerosis | HC | 0.5979 | 0.0144 | 0.0159 | NA |
| Coronary atherosclerosis | TFP | 0.2141 | 0.4290 | 0.4119 | NA |
| Coronary atherosclerosis | WC | 0.4339 | 0.0057 | 0.0060 | NA |
| Coronary heart disease | BMI | 0.8359 | 0.0291 | 0.0332 | NA |
| Coronary heart disease | HC | 0.7760 | 0.0600 | 0.0666 | NA |
| Coronary heart disease | TFP | 0.6008 | 0.0005 | 0.0006 | NO |
| Coronary heart disease | WC | 0.8021 | 0.0505 | 0.0578 | NA |
| Ectopic pregnancy | BMI | 0.4936 | 0.2216 | 0.2305 | NA |
| Ectopic pregnancy | HC | 0.4598 | 0.8750 | 0.8799 | NA |
| Ectopic pregnancy | TFP | 0.2269 | 0.4293 | 0.4140 | NA |
| Ectopic pregnancy | WC | 0.8992 | 0.1746 | 0.1925 | NA |
| Female infertility | BMI | 0.9257 | 0.4480 | 0.4738 | NA |
| Female infertility | HC | 0.5481 | 0.5286 | 0.5437 | NA |
| Female infertility | TFP | 0.7272 | 0.1229 | 0.1374 | NA |
| Female infertility | WC | 0.4681 | 0.0318 | 0.0338 | NA |
| Fracture of lower leg including ankle | BMI | 0.9963 | 0.0687 | 0.0742 | NA |
| Fracture of lower leg including ankle | HC | 0.6908 | 0.0918 | 0.0982 | NA |
| Fracture of lower leg including ankle | TFP | 0.9594 | 0.0365 | 0.0410 | NA |
| Fracture of lower leg including ankle | WC | 0.8735 | 0.3825 | 0.4011 | NA |
| Fracture of lumbar spine and pelvis | BMI | 0.8355 | 0.1601 | 0.1697 | NA |
| Fracture of lumbar spine and pelvis | HC | 0.9974 | 0.2810 | 0.2967 | NA |
| Fracture of lumbar spine and pelvis | TFP | 0.5322 | 0.2760 | 0.2871 | NA |
| Fracture of lumbar spine and pelvis | WC | 0.0580 | 0.5819 | 0.5062 | NA |
| Gestational diabetes | BMI | 0.6098 | 0.1877 | 0.1981 | NA |
| Gestational diabetes | HC | 0.2038 | 0.0012 | 0.0010 | NO |
| Gestational diabetes | TFP | 0.8257 | 0.0116 | 0.0137 | NA |
| Gestational diabetes | WC | 0.4057 | 0.2556 | 0.2611 | NA |
| Heart failure | BMI | 0.7961 | 0.0527 | 0.0587 | NA |
| Heart failure | HC | 0.1872 | 0.4558 | 0.4384 | NA |
| Heart failure | TFP | 0.7021 | 0.0257 | 0.0298 | NO |
| Heart failure | WC | 0.1041 | 0.9497 | 0.9319 | NA |
| Hypertension | BMI | 0.8887 | 0.0000 | 0.0000 | NO |
| Hypertension | HC | 0.3590 | 0.0000 | 0.0000 | NA |
| Hypertension | TFP | 0.1718 | 0.0000 | 0.0000 | NO |
| Hypertension | WC | 0.5253 | 0.0113 | 0.0124 | NA |
| Infections of the skin and subcutaneous tissue | BMI | 0.8427 | 0.3321 | 0.3544 | NA |
| Infections of the skin and subcutaneous tissue | HC | 0.9652 | 0.1394 | 0.1526 | NA |
| Infections of the skin and subcutaneous tissue | TFP | 0.1547 | 0.1527 | 0.1315 | NA |
| Infections of the skin and subcutaneous tissue | WC | 0.1462 | 0.6514 | 0.6181 | NA |
| Insulin receptor protein | BMI | 0.2229 | 0.4385 | 0.4290 | NA |
| Insulin receptor protein | HC | 0.5731 | 0.3080 | 0.3195 | NA |
| Insulin receptor protein | TFP | 0.3007 | 0.5533 | 0.5509 | NA |
| Insulin receptor protein | WC | 0.9174 | 0.2913 | 0.3111 | NA |
| Ischemic stroke | BMI | 0.6315 | 0.0008 | 0.0009 | NA |
| Ischemic stroke | HC | 0.6920 | 0.4380 | 0.4583 | NA |
| Ischemic stroke | TFP | 0.4896 | 0.3462 | 0.3619 | NA |
| Ischemic stroke | WC | 0.3387 | 0.0151 | 0.0149 | NA |
| Malaise and fatigue | BMI | 0.9550 | 0.0266 | 0.0305 | NO |
| Malaise and fatigue | HC | 0.4921 | 0.8847 | 0.8904 | NA |
| Malaise and fatigue | TFP | 0.8039 | 0.1699 | 0.1892 | NA |
| Malaise and fatigue | WC | 0.3377 | 0.3920 | 0.3936 | NA |
| Malignant neoplasm of kidney | BMI | 0.8753 | 0.9148 | 0.9240 | NA |
| Malignant neoplasm of kidney | HC | 0.6930 | 0.1018 | 0.1105 | NA |
| Malignant neoplasm of kidney | TFP | 0.4486 | 0.0860 | 0.0905 | NA |
| Malignant neoplasm of kidney | WC | 0.9348 | 0.1859 | 0.2054 | NA |
| Metabolic disorders | BMI | 0.5616 | 0.1165 | 0.1237 | NA |
| Metabolic disorders | HC | 0.2605 | 0.2179 | 0.2125 | NA |
| Metabolic disorders | TFP | 0.5168 | 0.0136 | 0.0150 | NA |
| Metabolic disorders | WC | 0.8833 | 0.0028 | 0.0034 | NA |
| Nonalcoholic fatty liver disease | BMI | 0.5294 | 0.0363 | 0.0393 | NA |
| Nonalcoholic fatty liver disease | HC | 0.2449 | 0.0442 | 0.0418 | NA |
| Nonalcoholic fatty liver disease | TFP | 0.2013 | 0.0223 | 0.0189 | NA |
| Nonalcoholic fatty liver disease | WC | 0.4873 | 0.0026 | 0.0028 | NO |
| Nontraumatic intracranial haemmorrhage | BMI | 0.9445 | 0.2784 | 0.3006 | NA |
| Nontraumatic intracranial haemmorrhage | HC | 0.5749 | 0.1801 | 0.1908 | NA |
| Nontraumatic intracranial haemmorrhage | TFP | 0.2184 | 0.6088 | 0.5901 | NA |
| Nontraumatic intracranial haemmorrhage | WC | 0.6937 | 0.2074 | 0.2253 | NA |
| Peripheral atherosclerosis | BMI | 0.5652 | 0.0034 | 0.0037 | NO |
| Peripheral atherosclerosis | HC | 0.7030 | 0.0072 | 0.0082 | NO |
| Peripheral atherosclerosis | TFP | 0.5740 | 0.2858 | 0.3043 | NA |
| Peripheral atherosclerosis | WC | 0.7341 | 0.2148 | 0.2329 | NA |
| Puerperal sepsis | BMI | 0.4024 | 0.0781 | 0.0803 | NA |
| Puerperal sepsis | HC | 0.9145 | 0.6354 | 0.6574 | NA |
| Puerperal sepsis | TFP | 0.1430 | 0.2385 | 0.2076 | NA |
| Puerperal sepsis | WC | 0.7252 | 0.3914 | 0.4157 | NA |
| Rosacea | BMI | 0.4948 | 0.4936 | 0.5075 | NA |
| Rosacea | HC | 0.1479 | 0.4458 | 0.4196 | NA |
| Rosacea | TFP | 0.5532 | 0.4909 | 0.5120 | NA |
| Rosacea | WC | 0.3859 | 0.1981 | 0.2020 | NA |
| Sleep disorders | BMI | 0.1688 | 0.0056 | 0.0045 | NA |
| Sleep disorders | HC | 0.1968 | 0.0727 | 0.0664 | NA |
| Sleep disorders | TFP | 0.4993 | 0.0097 | 0.0106 | NA |
| Sleep disorders | WC | 0.9570 | 0.0136 | 0.0162 | NA |
| Stroke | BMI | 0.4430 | 0.0060 | 0.0063 | NA |
| Stroke | HC | 0.3472 | 0.1846 | 0.1856 | NA |
| Stroke | TFP | 0.3050 | 0.2888 | 0.2858 | NA |
| Stroke | WC | 0.2988 | 0.0334 | 0.0321 | NA |
| Type 2 diabetes | BMI | 0.7621 | 0.0000 | 0.0000 | NO |
| Type 2 diabetes | HC | 0.0755 | 0.0000 | 0.0000 | NO |
| Type 2 diabetes | TFP | 0.2934 | 0.0000 | 0.0000 | NO |
| Type 2 diabetes | WC | 0.8988 | 0.0010 | 0.0012 | NO |
| Urinary incontinence | BMI | 0.2030 | 0.7952 | 0.7787 | NA |
| Urinary incontinence | HC | 0.1365 | 0.0137 | 0.0110 | NO |
| Urinary incontinence | TFP | 0.1364 | 0.3152 | 0.2787 | NA |
| Urinary incontinence | WC | 0.1731 | 0.0721 | 0.0624 | NA |
| Varicose veins | BMI | 0.8613 | 0.0713 | 0.0796 | NA |
| Varicose veins | HC | 0.1563 | 0.0021 | 0.0017 | NO |
| Varicose veins | TFP | 0.6440 | 0.3344 | 0.3582 | NA |
| Varicose veins | WC | 0.5640 | 0.3106 | 0.3279 | NO |
| Acute pancreatitis | BMI | 0.1810 | 0.6327 | 0.6052 | NA |
| Acute pancreatitis | HC | 0.1948 | 0.5530 | 0.5391 | NA |
| Acute pancreatitis | TFP | 0.6651 | 0.5164 | 0.5353 | NA |
| Acute pancreatitis | WC | 0.1963 | 0.2314 | 0.2198 | NA |
| Alzheimer's disease | BMI | 0.0974 | 0.0491 | 0.0344 | NO |
| Alzheimer's disease | HC | 0.9591 | 0.0207 | 0.0236 | NA |
| Alzheimer's disease | TFP | 0.4286 | 0.0098 | 0.0103 | NA |
| Alzheimer's disease | WC | 0.2747 | 0.0000 | 0.0000 | NA |
| Asthma | BMI | 0.5196 | 0.0001 | 0.0002 | NO |
| Asthma | HC | 0.8374 | 0.0000 | 0.0000 | NO |
| Asthma | TFP | 0.7951 | 0.0001 | 0.0001 | NO |
| Asthma | WC | 0.2469 | 0.0000 | 0.0000 | NO |
| Breast cancer | BMI | 0.8288 | 0.0025 | 0.0031 | NO |
| Breast cancer | HC | 0.7181 | 0.0012 | 0.0014 | NA |
| Breast cancer | TFP | 0.8042 | 0.0152 | 0.0179 | NA |
| Breast cancer | WC | 0.4920 | 0.0003 | 0.0003 | NA |
| Chronic obstructive pulmonary disease | BMI | 0.9864 | 0.2663 | 0.2900 | NA |
| Chronic obstructive pulmonary disease | HC | 0.4021 | 0.0356 | 0.0365 | NA |
| Chronic obstructive pulmonary disease | TFP | 0.1041 | 0.0005 | 0.0002 | NO |
| Chronic obstructive pulmonary disease | WC | 0.0790 | 0.0069 | 0.0047 | NO |
| Colon cancer | BMI | 0.6356 | 0.4733 | 0.5009 | NA |
| Colon cancer | HC | 0.3258 | 0.6023 | 0.6026 | NA |
| Colon cancer | TFP | 0.1172 | 0.6723 | 0.6252 | NA |
| Colon cancer | WC | 0.0715 | 0.8130 | 0.7691 | NA |
| Depression | BMI | 0.4287 | 0.5445 | 0.5549 | NA |
| Depression | HC | 0.1910 | 0.0017 | 0.0014 | NA |
| Depression | TFP | 0.3921 | 0.4250 | 0.4325 | NA |
| Depression | WC | 0.2089 | 0.0160 | 0.0143 | NO |
| Endometrial cancer | BMI | 0.4119 | 0.0022 | 0.0023 | NA |
| Endometrial cancer | HC | 0.8595 | 0.0839 | 0.0919 | NA |
| Endometrial cancer | TFP | 0.8495 | 0.1106 | 0.1234 | NA |
| Endometrial cancer | WC | 0.1605 | 0.0372 | 0.0326 | NA |
| Esophageal cancer | BMI | 0.1775 | 0.5981 | 0.5734 | NA |
| Esophageal cancer | HC | 0.8733 | 0.0744 | 0.0818 | NA |
| Esophageal cancer | TFP | 0.7659 | 0.7651 | 0.7849 | NA |
| Esophageal cancer | WC | 0.6484 | 0.3845 | 0.4005 | NA |
| Fasting insulin | BMI | 0.2813 | 0.0212 | 0.0199 | NA |
| Fasting insulin | HC | 0.8455 | 0.0000 | 0.0000 | NA |
| Fasting insulin | TFP | 0.9489 | 0.0000 | 0.0000 | NA |
| Fasting insulin | WC | 0.0859 | 0.0000 | 0.0000 | NO |
| Gastroesophageal reflux disease | BMI | 0.2799 | 0.0428 | 0.0379 | NA |
| Gastroesophageal reflux disease | HC | 0.1110 | 0.0003 | 0.0001 | NA |
| Gastroesophageal reflux disease | TFP | 0.0638 | 0.0272 | 0.0127 | NA |
| Gastroesophageal reflux disease | WC | 0.7506 | 0.0039 | 0.0050 | NA |
| Gout | BMI | 0.3516 | 0.0000 | 0.0000 | NA |
| Gout | HC | 0.8776 | 0.0000 | 0.0000 | NO |
| Gout | TFP | 0.2873 | 0.0000 | 0.0000 | NA |
| Gout | WC | 0.7363 | 0.0052 | 0.0059 | NA |
| Hyperthyroidism | BMI | 0.2706 | 0.5172 | 0.5100 | NA |
| Hyperthyroidism | HC | 0.1098 | 0.2820 | 0.2521 | NA |
| Hyperthyroidism | TFP | 0.4888 | 0.4052 | 0.4209 | NA |
| Hyperthyroidism | WC | 0.2330 | 0.1058 | 0.1008 | NA |
| Hypothyroidism | BMI | 0.1823 | 0.0003 | 0.0002 | NA |
| Hypothyroidism | HC | 0.6965 | 0.0943 | 0.1029 | NA |
| Hypothyroidism | TFP | 0.0760 | 0.0000 | 0.0000 | NO |
| Hypothyroidism | WC | 0.8325 | 0.0001 | 0.0001 | NA |
| Insulin resistance | BMI | 0.3198 | 0.1814 | 0.1795 | NA |
| Insulin resistance | HC | 0.3167 | 0.4993 | 0.4989 | NA |
| Insulin resistance | TFP | 0.1106 | 0.0009 | 0.0005 | NA |
| Insulin resistance | WC | 0.1595 | 0.1562 | 0.1355 | NA |
| Osteoarthritis | BMI | 0.0529 | 0.0248 | 0.0176 | NA |
| Osteoarthritis | HC | 0.1074 | 0.0001 | 0.0001 | NO |
| Osteoarthritis | TFP | 0.8991 | 0.0001 | 0.0001 | NO |
| Osteoarthritis | WC | 0.4120 | 0.2751 | 0.2809 | NA |
| Osteoporosis | BMI | 0.2287 | 0.0000 | 0.0000 | NA |
| Osteoporosis | HC | 0.4481 | 0.0015 | 0.0016 | NA |
| Osteoporosis | TFP | 0.9152 | 0.0001 | 0.0001 | NA |
| Osteoporosis | WC | 0.7843 | 0.0061 | 0.0082 | NO |
| Parkinson's disease | BMI | 0.4483 | 0.9081 | 0.9125 | NA |
| Parkinson's disease | HC | 0.4986 | 0.1742 | 0.1813 | NA |
| Parkinson's disease | TFP | 0.8625 | 0.2474 | 0.2694 | NA |
| Parkinson's disease | WC | 0.6632 | 0.2055 | 0.2177 | NA |
| Polycystic ovary syndrome | BMI | 0.5613 | 0.8111 | 0.8246 | NA |
| Polycystic ovary syndrome | HC | 0.2168 | 0.3988 | 0.3865 | NA |
| Polycystic ovary syndrome | TFP | 0.8119 | 0.0391 | 0.0453 | NA |
| Polycystic ovary syndrome | WC | 0.3108 | 0.5745 | 0.5735 | NA |
| Preeclampsia | BMI | 0.9370 | 0.6325 | 0.6628 | NA |
| Preeclampsia | HC | 0.0910 | 0.3478 | 0.3104 | NA |
| Preeclampsia | TFP | 0.3978 | 0.1423 | 0.1459 | NA |
| Preeclampsia | WC | 0.0694 | 0.5154 | 0.4673 | NA |
| Residual haemorrhoidal skin tags | BMI | 0.9983 | 0.8645 | 0.8812 | NA |
| Residual haemorrhoidal skin tags | HC | 0.8701 | 0.3728 | 0.3953 | NA |
| Residual haemorrhoidal skin tags | TFP | 0.2673 | 0.7342 | 0.7260 | NA |
| Residual haemorrhoidal skin tags | WC | 0.2232 | 0.9638 | 0.9601 | NA |
| Sex hormone binding globulin | BMI | 0.0809 | 0.0003 | 0.0001 | NA |
| Sex hormone binding globulin | HC | 0.2551 | 0.0000 | 0.0000 | NO |
| Sex hormone binding globulin | TFP | 0.4889 | 0.0000 | 0.0000 | NO |
| Sex hormone binding globulin | WC | 0.0000 | 0.0000 | 0.0000 | NO |
| Sleep apnea syndrome | BMI | 0.2899 | 0.0117 | 0.0108 | NA |
| Sleep apnea syndrome | HC | 0.1492 | 0.0000 | 0.0000 | NO |
| Sleep apnea syndrome | TFP | 0.9524 | 0.0000 | 0.0000 | NO |
| Sleep apnea syndrome | WC | 0.8299 | 0.0009 | 0.0010 | NA |
| Sporadic miscarriage | BMI | 0.5877 | 0.2481 | 0.2635 | NA |
| Sporadic miscarriage | HC | 0.4142 | 0.1036 | 0.1063 | NA |
| Sporadic miscarriage | TFP | 0.4495 | 0.2044 | 0.2121 | NA |
| Sporadic miscarriage | WC | 0.0616 | 0.4827 | 0.4297 | NA |
| Type 1 diabetes | BMI | 0.1726 | 0.2137 | 0.1959 | NA |
| Type 1 diabetes | HC | 0.6743 | 0.8339 | 0.8453 | NA |
| Type 1 diabetes | TFP | 0.4489 | 0.9212 | 0.9248 | NA |
| Type 1 diabetes | WC | 0.9592 | 0.7338 | 0.7523 | NA |
| Serum uric acid | BMI | 0.0779 | 0.2408 | 0.1481 | NA |
| Serum uric acid | HC | 0.2125 | 0.0000 | 0.0000 | NO |
| Serum uric acid | WC | 0.0688 | 0.0000 | 0.0000 | NO |
| Serum uric acid | TFP | 0.0778 | 0.0000 | 0.0000 | NO |
| Intervertebral disk disorders | BMI | 0.3954 | 0.1009 | 0.1029 | NA |
| Intervertebral disk disorders | HC | 0.8103 | 0.0911 | 0.0993 | NA |
| Intervertebral disk disorders | WC | 0.7976 | 0.0946 | 0.1043 | NA |
| Intervertebral disk disorders | TFP | 0.4970 | 0.0162 | 0.0175 | NA |
| Lumbar spine bone mineral density | BMI | 0.4575 | 0.0077 | 0.0080 | NA |
| Lumbar spine bone mineral density | HC | 0.7024 | 0.0001 | 0.0001 | NO |
| Lumbar spine bone mineral density | WC | 0.7027 | 0.0013 | 0.0014 | NA |
| Lumbar spine bone mineral density | TFP | 0.6059 | 0.0176 | 0.0193 | NO |
| Inguinal or femoral hernia, bilateral | BMI | 0.7059 | 0.0185 | 0.0202 | NA |
| Inguinal or femoral hernia, bilateral | HC | 0.5806 | 0.1127 | 0.1190 | NA |
| Inguinal or femoral hernia, bilateral | WC | 0.7296 | 0.0300 | 0.0331 | NA |
| Inguinal or femoral hernia, bilateral | TFP | 0.3005 | 0.0418 | 0.0408 | NA |
| Heel bone mineral density | BMI | 0.5727 | 0.0000 | 0.0000 | NO |
| Heel bone mineral density | HC | 0.5038 | 0.0008 | 0.0009 | NA |
| Heel bone mineral density | WC | 0.2910 | 0.0000 | 0.0000 | NO |
| Heel bone mineral density | TFP | 0.6506 | 0.0004 | 0.0005 | NA |
| Total body bone mineral density (age over 60) | BMI | 0.7634 | 0.0000 | 0.0000 | NA |
| Total body bone mineral density (age over 60) | HC | 0.2675 | 0.0000 | 0.0000 | NO |
| Total body bone mineral density (age over 60) | WC | 0.6579 | 0.0003 | 0.0004 | NO |
| Total body bone mineral density (age over 60) | TFP | 0.3206 | 0.0074 | 0.0073 | NA |
| Total body bone mineral density (age 45–60) | BMI | 0.0986 | 0.0098 | 0.0078 | NA |
| Total body bone mineral density (age 45–60) | HC | 0.1632 | 0.0558 | 0.0456 | NA |
| Total body bone mineral density (age 45–60) | WC | 0.9055 | 0.1341 | 0.1451 | NA |
| Total body bone mineral density (age 45–60) | TFP | 0.3246 | 0.0003 | 0.0003 | NO |
| Total body bone mineral density (age 30–45) | BMI | 0.7571 | 0.0245 | 0.0268 | NA |
| Total body bone mineral density (age 30–45) | HC | 0.5914 | 0.0236 | 0.0254 | NA |
| Total body bone mineral density (age 30–45) | WC | 0.1454 | 0.0400 | 0.0346 | NO |
| Total body bone mineral density (age 30–45) | TFP | 0.9740 | 0.0004 | 0.0005 | NO |
| Rheumatoid arthritis | BMI | 0.3237 | 0.6073 | 0.6075 | NA |
| Rheumatoid arthritis | HC | 0.4414 | 0.7821 | 0.7872 | NA |
| Rheumatoid arthritis | WC | 0.8005 | 0.5254 | 0.5433 | NA |
| Rheumatoid arthritis | TFP | 0.8558 | 0.3886 | 0.4083 | NA |
| Systemic lupus erythematosus | BMI | 0.2691 | 0.0728 | 0.0710 | NA |
| Systemic lupus erythematosus | HC | 0.5506 | 0.6857 | 0.6962 | NA |
| Systemic lupus erythematosus | WC | 0.6410 | 0.1409 | 0.1499 | NA |
| Systemic lupus erythematosus | TFP | 0.6988 | 0.6550 | 0.6710 | NA |
| Multiple sclerosis | BMI | 0.4725 | 0.0958 | 0.0989 | NA |
| Multiple sclerosis | HC | 0.5165 | 0.4252 | 0.4353 | NA |
| Multiple sclerosis | WC | 0.6427 | 0.4823 | 0.4973 | NA |
| Multiple sclerosis | TFP | 0.1743 | 0.0195 | 0.0171 | NA |
| Crohn's disease | BMI | 0.4917 | 0.0002 | 0.0002 | NO |
| Crohn's disease | HC | 0.3877 | 0.0006 | 0.0006 | NA |
| Crohn's disease | WC | 0.3928 | 0.0009 | 0.0009 | NA |
| Crohn's disease | TFP | 0.9237 | 0.0000 | 0.0000 | NO |
| Ulcerative colitis | BMI | 0.1688 | 0.0890 | 0.0829 | NA |
| Ulcerative colitis | HC | 0.6661 | 0.7483 | 0.7599 | NA |
| Ulcerative colitis | WC | 0.3680 | 0.5158 | 0.5193 | NA |
| Ulcerative colitis | TFP | 0.9003 | 0.4862 | 0.5070 | NA |
| Myasthenia Gravis | BMI | 0.4922 | 0.1832 | 0.1896 | NA |
| Myasthenia Gravis | HC | 0.4855 | 0.0109 | 0.0116 | NA |
| Myasthenia Gravis | WC | 0.7791 | 0.1889 | 0.2028 | NA |
| Myasthenia Gravis | TFP | 0.0819 | 0.1354 | 0.0921 | NA |
| Systemic sclerosis | BMI | 0.5975 | 0.9722 | 0.9740 | NA |
| Systemic sclerosis | HC | 0.7566 | 0.5720 | 0.5879 | NA |
| Systemic sclerosis | WC | 0.6674 | 0.7548 | 0.7670 | NA |
| Systemic sclerosis | TFP | 0.2857 | 0.4109 | 0.4077 | NA |
| Idiopathic thrombocytopenic purpura | BMI | 0.5573 | 0.7273 | 0.7369 | NA |
| Idiopathic thrombocytopenic purpura | HC | 0.8967 | 0.4722 | 0.4905 | NA |
| Idiopathic thrombocytopenic purpura | WC | 0.6309 | 0.7990 | 0.8100 | NA |
| Idiopathic thrombocytopenic purpura | TFP | 0.6285 | 0.9182 | 0.9240 | NA |
| Schizophrenia | BMI | 0.7589 | 0.6705 | 0.6836 | NA |
| Schizophrenia | HC | 0.4991 | 0.1217 | 0.1264 | NA |
| Schizophrenia | WC | 0.1956 | 0.0005 | 0.0004 | NA |
| Schizophrenia | TFP | 0.9501 | 0.0223 | 0.0252 | NO |
| Irritable bowel syndrome | BMI | 0.7137 | 0.0000 | 0.0000 | NA |
| Irritable bowel syndrome | HC | 0.5561 | 0.0000 | 0.0000 | NA |
| Irritable bowel syndrome | WC | 0.3455 | 0.0001 | 0.0001 | NA |
| Irritable bowel syndrome | TFP | 0.2011 | 0.0000 | 0.0000 | NA |
| Erectile dysfunction | BMI | 0.1959 | 0.5609 | 0.5488 | NA |
| Erectile dysfunction | HC | 0.2859 | 0.1964 | 0.1941 | NA |
| Erectile dysfunction | WC | 0.6665 | 0.8431 | 0.8531 | NA |
| Erectile dysfunction | TFP | 0.4253 | 0.3811 | 0.3880 | NA |
| Male infertility | BMI | 0.0881 | 0.5228 | 0.4922 | NA |
| Male infertility | HC | 0.5026 | 0.1047 | 0.1089 | NA |
| Male infertility | WC | 0.4397 | 0.8854 | 0.8888 | NA |
| Male infertility | TFP | 0.1168 | 0.1082 | 0.0934 | NA |
| Pure hypercholesterolaemia | BMI | 0.6066 | 0.5390 | 0.5601 | NA |
| Pure hypercholesterolaemia | HC | 0.5491 | 0.6243 | 0.6443 | NA |
| Pure hypercholesterolaemia | WC | 0.9903 | 0.4775 | 0.5150 | NA |
| Pure hypercholesterolaemia | TFP | 0.1201 | 0.2256 | 0.1727 | NA |
| Familial combined hyperlipidemia | BMI | 0.3025 | 0.3922 | 0.3895 | NA |
| Familial combined hyperlipidemia | HC | 0.2003 | 0.0567 | 0.0485 | NA |
| Familial combined hyperlipidemia | WC | 0.4321 | 0.5440 | 0.5605 | NA |
| Familial combined hyperlipidemia | TFP | 0.3504 | 0.5722 | 0.5761 | NA |
| Malignant neoplasm of prostate | BMI | 0.6957 | 0.4155 | 0.4316 | NA |
| Malignant neoplasm of prostate | HC | 0.9063 | 0.2516 | 0.2683 | NA |
| Malignant neoplasm of prostate | WC | 0.8010 | 0.1745 | 0.1895 | NA |
| Malignant neoplasm of prostate | TFP | 0.8559 | 0.4555 | 0.4823 | NA |
| Prostatitis | BMI | 0.1886 | 0.0142 | 0.0118 | NA |
| Prostatitis | HC | 0.7615 | 0.1690 | 0.1811 | NA |
| Prostatitis | WC | 0.0579 | 0.6917 | 0.6288 | NA |
| Prostatitis | TFP | 0.4099 | 0.2644 | 0.2709 | NA |
| Sepsis | BMI | 0.1438 | 0.0872 | 0.0729 | NA |
| Sepsis | HC | 0.2195 | 0.2960 | 0.2798 | NA |
| Sepsis | WC | 0.2747 | 0.0487 | 0.0453 | NA |
| Sepsis | TFP | 0.3364 | 0.0026 | 0.0024 | NA |
| Barrett's esophagus | BMI | 0.4954 | 0.0048 | 0.0053 | NA |
| Barrett's esophagus | HC | 0.5568 | 0.0037 | 0.0042 | NO |
| Barrett's esophagus | WC | 0.8662 | 0.2239 | 0.2592 | NA |
| Barrett's esophagus | TFP | 0.3586 | 0.0527 | 0.0524 | NA |
| Lung adenocarcinoma | BMI | 0.6881 | 0.0751 | 0.0845 | NA |
| Lung adenocarcinoma | HC | 0.9206 | 0.0810 | 0.0925 | NA |
| Lung adenocarcinoma | WC | 0.3126 | 0.3531 | 0.3500 | NA |
| Lung adenocarcinoma | TFP | 0.3456 | 0.0115 | 0.0112 | NA |
| Colon adenocarcinoma | BMI | 0.4693 | 0.7132 | 0.7236 | NA |
| Colon adenocarcinoma | HC | 0.3446 | 0.0215 | 0.0214 | NA |
| Colon adenocarcinoma | WC | 0.3756 | 0.2267 | 0.2309 | NA |
| Colon adenocarcinoma | TFP | 0.7716 | 0.1516 | 0.1746 | NA |
| Mild age-related type 2 diabetes | BMI | 0.6987 | 0.2601 | 0.2782 | NA |
| Mild age-related type 2 diabetes | HC | 0.1892 | 0.2671 | 0.2503 | NA |
| Mild age-related type 2 diabetes | WC | 0.7162 | 0.5218 | 0.5512 | NA |
| Mild age-related type 2 diabetes | TFP | 0.3009 | 0.5420 | 0.5392 | NA |
| Pulmonary fibrosis | BMI | 0.4603 | 0.3934 | 0.4068 | NA |
| Pulmonary fibrosis | HC | 0.5027 | 0.7336 | 0.7465 | NA |
| Pulmonary fibrosis | WC | 0.4006 | 0.9240 | 0.9263 | NA |
| Pulmonary fibrosis | TFP | 0.6627 | 0.1353 | 0.1531 | NA |
| Acute laryngitis and tracheitis | BMI | 0.7264 | 0.0707 | 0.0788 | NA |
| Acute laryngitis and tracheitis | HC | 0.2675 | 0.6038 | 0.5966 | NA |
| Acute laryngitis and tracheitis | WC | 0.4844 | 0.7851 | 0.7973 | NA |
| Acute laryngitis and tracheitis | TFP | 0.0879 | 0.2718 | 0.2024 | NA |
| Chronic laryngitis and laryngotracheitis | BMI | 0.7115 | 0.9423 | 0.9488 | NA |
| Chronic laryngitis and laryngotracheitis | HC | 0.2770 | 0.9527 | 0.9501 | NA |
| Chronic laryngitis and laryngotracheitis | WC | 0.1458 | 0.3403 | 0.3023 | NA |
| Chronic laryngitis and laryngotracheitis | TFP | 0.3753 | 0.2189 | 0.2233 | NA |
| urinary tract infection | BMI | 0.2544 | 0.3176 | 0.3074 | NA |
| urinary tract infection | HC | 0.0981 | 0.0829 | 0.0609 | NA |
| urinary tract infection | WC | 0.3618 | 0.6534 | 0.6580 | NA |
| urinary tract infection | TFP | 0.0821 | 0.1898 | 0.1249 | NA |

Note: *p-value from MR-Egger pleiotropy test; ^a^ p-value from MR-Egger heterogeneity test; ^b^ p-value from inverse-variance weighted heterogeneity test; **^#^** outliers’ results from Mendelian randomization pleiotropy RESidual sum and outlier test; For outliers test results, "NA" indicates no outliers were detected; "NO" indicates outliers were detected but the direction of odds ratios and statistical significance remained consistent before and after outlier removal. Statistical significance was defined as p < 0.05.

Abbreviations: BMI, body mass index; HC, hip circumference; TFP, total fat percentage; WC, waist circumference.
