# Supplementary material for: Unraveling the causal web of 4 adiposity indices and 92 multi-system outcomes: A body-wide Mendelian randomization study
Source: Medicine (Baltimore). 2026 May 22;105(21):e48986. doi: 10.1097/MD.0000000000048986 (PMC13201005; doi:10.1097/MD.0000000000048986)
Supplement: Supplementary file 7 [file medi-105-e48986-s007.docx]

Table S7. Independent causal effects of adiposity indices on 25 outcomes after adjusting for other adiposity indices in multivariate Mendelian randomization analyses.

| **Outcome** | **Exposure** | **nSNP** | **Method** | **Effect size (95%CI)** | **p-value** | **Pleiotropy test*** |
| --- | --- | --- | --- | --- | --- | --- |
| Acute pancreatitis | BMI | 367 | MVMR-IVW | 0.958 (0.589, 1.558) | 0.8627 | 0.364 |
|  |  | 367 | MVMR-Egger | 0.876 (0.519, 1.478) | 0.6194 |  |
|  | WC | 367 | MVMR-IVW | 1.572 (0.814, 3.037) | 0.1783 |  |
|  |  | 367 | MVMR-Egger | 1.614 (0.834, 3.127) | 0.1556 |  |
|  | TFP | 367 | MVMR-IVW | 1.604 (1.007, 2.555) | 0.0466 |  |
|  |  | 367 | MVMR-Egger | 1.551 (0.969, 2.485) | 0.0676 |  |
|  | HC | 367 | MVMR-IVW | 0.629 (0.425, 0.933) | 0.0211 |  |
|  |  | 367 | MVMR-Egger | 0.603 (0.402, 0.903) | 0.0142 |  |
| Asthma | BMI | 324 | MVMR-IVW | 0.959 (0.756, 1.218) | 0.732 | 0.739 |
|  |  | 324 | MVMR-Egger | 0.947 (0.738, 1.216) | 0.6705 |  |
|  | WC | 324 | MVMR-IVW | 1.425 (1.032, 1.970) | 0.0316 |  |
|  |  | 324 | MVMR-Egger | 1.429 (1.034, 1.977) | 0.0307 |  |
|  | TFP | 324 | MVMR-IVW | 1.187 (0.945, 1.491) | 0.1395 |  |
|  |  | 324 | MVMR-Egger | 1.177 (0.931, 1.488) | 0.1735 |  |
|  | HC | 324 | MVMR-IVW | 0.905 (0.748, 1.095) | 0.305 |  |
|  |  | 324 | MVMR-Egger | 0.899 (0.740, 1.092) | 0.2845 |  |
| Atrial fibrillation and flutter | BMI | 393 | MVMR-IVW | 1.150 (0.801, 1.651) | 0.4504 | 0.441 |
|  |  | 393 | MVMR-Egger | 1.139 (0.793, 1.637) | 0.4809 |  |
|  | WC | 393 | MVMR-IVW | 2.134 (1.265, 3.602) | 0.0045 |  |
|  |  | 393 | MVMR-Egger | 2.026 (1.181, 3.476) | 0.0104 |  |
|  | TFP | 393 | MVMR-IVW | 0.697 (0.465, 1.045) | 0.0808 |  |
|  |  | 393 | MVMR-Egger | 0.676 (0.447, 1.022) | 0.0631 |  |
|  | HC | 393 | MVMR-IVW | 1.372 (0.965, 1.950) | 0.0782 |  |
|  |  | 393 | MVMR-Egger | 1.339 (0.937, 1.914) | 0.1087 |  |
| Cholecystitis | BMI | 394 | MVMR-IVW | 1.085 (0.826, 1.427) | 0.5574 | 0.516 |
|  |  | 394 | MVMR-Egger | 1.092 (0.830, 1.436) | 0.5313 |  |
|  | WC | 394 | MVMR-IVW | 2.089 (1.406, 3.103) | 0.0003 |  |
|  |  | 394 | MVMR-Egger | 2.159 (1.435, 3.248) | 0.0002 |  |
|  | TFP | 394 | MVMR-IVW | 1.085 (0.800, 1.473) | 0.6 |  |
|  |  | 394 | MVMR-Egger | 1.106 (0.810, 1.509) | 0.5262 |  |
|  | HC | 394 | MVMR-IVW | 0.827 (0.635, 1.079) | 0.1614 |  |
|  |  | 394 | MVMR-Egger | 0.841 (0.642, 1.101) | 0.2066 |  |
| Cholelithiasis | BMI | 394 | MVMR-IVW | 1.045 (0.791, 1.382) | 0.7561 | 0.47 |
|  |  | 394 | MVMR-Egger | 1.052 (0.795, 1.392) | 0.7225 |  |
|  | WC | 394 | MVMR-IVW | 2.185 (1.459, 3.273) | 0.0001 |  |
|  |  | 394 | MVMR-Egger | 2.269 (1.495, 3.442) | 0.0001 |  |
|  | TFP | 394 | MVMR-IVW | 1.133 (0.829, 1.547) | 0.4337 |  |
|  |  | 394 | MVMR-Egger | 1.157 (0.842, 1.589) | 0.3675 |  |
|  | HC | 394 | MVMR-IVW | 0.811 (0.618, 1.062) | 0.128 |  |
|  |  | 394 | MVMR-Egger | 0.825 (0.627, 1.086) | 0.1705 |  |
| Chronic obstructive pulmonary disease | BMI | 367 | MVMR-IVW | 1.434 (1.039, 1.981) | 0.0284 | 0.414 |
|  |  | 367 | MVMR-Egger | 1.360 (0.961, 1.925) | 0.0824 |  |
|  | WC | 367 | MVMR-IVW | 1.100 (0.710, 1.704) | 0.6698 |  |
|  |  | 367 | MVMR-Egger | 1.118 (0.720, 1.735) | 0.6201 |  |
|  | TFP | 367 | MVMR-IVW | 1.397 (1.026, 1.901) | 0.0336 |  |
|  |  | 367 | MVMR-Egger | 1.368 (1.001, 1.870) | 0.0489 |  |
|  | HC | 367 | MVMR-IVW | 0.892 (0.686, 1.159) | 0.3909 |  |
|  |  | 367 | MVMR-Egger | 0.869 (0.664, 1.137) | 0.3063 |  |
| Fracture of lower leg including ankle | BMI | 394 | MVMR-IVW | 1.106 (0.806, 1.519) | 0.5326 | 0.414 |
|  |  | 394 | MVMR-Egger | 1.097 (0.798, 1.508) | 0.5678 |  |
|  | WC | 394 | MVMR-IVW | 0.691 (0.437, 1.093) | 0.1142 |  |
|  |  | 394 | MVMR-Egger | 0.659 (0.410, 1.057) | 0.0834 |  |
|  | TFP | 394 | MVMR-IVW | 1.373 (0.964, 1.957) | 0.0792 |  |
|  |  | 394 | MVMR-Egger | 1.336 (0.932, 1.916) | 0.115 |  |
|  | HC | 394 | MVMR-IVW | 1.350 (0.993, 1.836) | 0.0554 |  |
|  |  | 394 | MVMR-Egger | 1.320 (0.966, 1.803) | 0.0815 |  |
| Gastroesophageal reflux disease | BMI | 274 | MVMR-IVW | 1.937 (1.584, 2.368) | 1.14E-10 | 0.0005 |
|  |  | 274 | MVMR-Egger | 1.680 (1.359, 2.077) | 1.68E-06 |  |
|  | WC | 274 | MVMR-IVW | 1.262 (0.948, 1.680) | 0.1114 |  |
|  |  | 274 | MVMR-Egger | 1.335 (1.007, 1.770) | 0.0448 |  |
|  | TFP | 274 | MVMR-IVW | 1.675 (1.389, 2.020) | 6.47E-08 |  |
|  |  | 274 | MVMR-Egger | 1.569 (1.301, 1.891) | 2.35E-06 |  |
|  | HC | 274 | MVMR-IVW | 0.566 (0.479, 0.669) | 2.27E-11 |  |
|  |  | 274 | MVMR-Egger | 0.532 (0.450, 0.628) | 1.26E-13 |  |
| Gestational diabetes | BMI | 394 | MVMR-IVW | 1.464 (0.927, 2.312) | 0.1022 | 0.801 |
|  |  | 394 | MVMR-Egger | 1.469 (0.929, 2.324) | 0.1 |  |
|  | WC | 394 | MVMR-IVW | 3.411 (1.763, 6.601) | 0.0003 |  |
|  |  | 394 | MVMR-Egger | 3.486 (1.763, 6.893) | 0.0003 |  |
|  | TFP | 394 | MVMR-IVW | 0.728 (0.437, 1.212) | 0.2223 |  |
|  |  | 394 | MVMR-Egger | 0.737 (0.438, 1.239) | 0.249 |  |
|  | HC | 394 | MVMR-IVW | 0.501 (0.322, 0.780) | 0.0022 |  |
|  |  | 394 | MVMR-Egger | 0.506 (0.323, 0.794) | 0.003 |  |
| Heart failure | BMI | 394 | MVMR-IVW | 1.331 (0.960, 1.847) | 0.0865 | 0.909 |
|  |  | 394 | MVMR-Egger | 1.333 (0.960, 1.851) | 0.0863 |  |
|  | WC | 394 | MVMR-IVW | 1.751 (1.091, 2.810) | 0.0203 |  |
|  |  | 394 | MVMR-Egger | 1.763 (1.082, 2.874) | 0.0229 |  |
|  | TFP | 394 | MVMR-IVW | 0.945 (0.656, 1.363) | 0.7633 |  |
|  |  | 394 | MVMR-Egger | 0.949 (0.654, 1.377) | 0.7833 |  |
|  | HC | 394 | MVMR-IVW | 0.916 (0.667, 1.257) | 0.5857 |  |
|  |  | 394 | MVMR-Egger | 0.919 (0.666, 1.268) | 0.6059 |  |
| Hypertension | BMI | 394 | MVMR-IVW | 2.368 (1.773, 3.163) | 5.27E-09 | 0.158 |
|  |  | 394 | MVMR-Egger | 2.400 (1.796, 3.206) | 3.15E-09 |  |
|  | WC | 394 | MVMR-IVW | 1.570 (1.034, 2.386) | 0.0345 |  |
|  |  | 394 | MVMR-Egger | 1.695 (1.101, 2.607) | 0.0165 |  |
|  | TFP | 394 | MVMR-IVW | 0.788 (0.570, 1.089) | 0.1484 |  |
|  |  | 394 | MVMR-Egger | 0.823 (0.593, 1.142) | 0.2438 |  |
|  | HC | 394 | MVMR-IVW | 0.656 (0.496, 0.868) | 0.0032 |  |
|  |  | 394 | MVMR-Egger | 0.680 (0.512, 0.904) | 0.0079 |  |
| Hypothyroidism | BMI | 322 | MVMR-IVW | 0.929 (0.673, 1.281) | 0.6533 | 0.582 |
|  |  | 322 | MVMR-Egger | 0.963 (0.681, 1.361) | 0.8298 |  |
|  | WC | 322 | MVMR-IVW | 1.120 (0.713, 1.761) | 0.6229 |  |
|  |  | 322 | MVMR-Egger | 1.109 (0.704, 1.746) | 0.6565 |  |
|  | TFP | 322 | MVMR-IVW | 1.020 (0.742, 1.402) | 0.9027 |  |
|  |  | 322 | MVMR-Egger | 1.036 (0.750, 1.433) | 0.8283 |  |
|  | HC | 322 | MVMR-IVW | 1.449 (1.107, 1.895) | 0.0069 |  |
|  |  | 322 | MVMR-Egger | 1.469 (1.117, 1.932) | 0.0059 |  |
| Infections of the skin and subcutaneous tissue | BMI | 394 | MVMR-IVW | 1.253 (0.914, 1.718) | 0.161 | 0.559 |
|  |  | 394 | MVMR-Egger | 1.261 (0.919, 1.730) | 0.1514 |  |
|  | WC | 394 | MVMR-IVW | 1.779 (1.128, 2.806) | 0.0132 |  |
|  |  | 394 | MVMR-Egger | 1.842 (1.150, 2.948) | 0.011 |  |
|  | TFP | 394 | MVMR-IVW | 0.900 (0.633, 1.280) | 0.5585 |  |
|  |  | 394 | MVMR-Egger | 0.918 (0.641, 1.314) | 0.6394 |  |
|  | HC | 394 | MVMR-IVW | 0.810 (0.597, 1.099) | 0.1762 |  |
|  |  | 394 | MVMR-Egger | 0.823 (0.604, 1.123) | 0.2196 |  |
| Insulin resistance | BMI | 280 | MVMR-IVW | 0.118 (−0.006, 0.242) | 0.0622 | 0.124 |
|  |  | 280 | MVMR-Egger | 0.155 (0.023, 0.287) | 0.0217 |  |
|  | WC | 280 | MVMR-IVW | 0.318 (0.145, 0.490) | 0.0003 |  |
|  |  | 280 | MVMR-Egger | 0.301 (0.127, 0.475) | 0.0007 |  |
|  | TFP | 280 | MVMR-IVW | −0.165 (−0.280, −0.050) | 0.0048 |  |
|  |  | 280 | MVMR-Egger | −0.150 (−0.265, −0.034) | 0.0118 |  |
|  | HC | 280 | MVMR-IVW | −0.156 (−0.259, −0.052) | 0.0031 |  |
|  |  | 280 | MVMR-Egger | −0.132 (−0.238, −0.025) | 0.0156 |  |
| Osteoarthritis | BMI | 330 | MVMR-IVW | 1.830 (1.432, 2.337) | 1.34E-06 | 0.65 |
|  |  | 330 | MVMR-Egger | 1.790 (1.376, 2.328) | 1.43E-05 |  |
|  | WC | 330 | MVMR-IVW | 0.888 (0.630, 1.252) | 0.4975 |  |
|  |  | 330 | MVMR-Egger | 0.893 (0.633, 1.262) | 0.5218 |  |
|  | TFP | 330 | MVMR-IVW | 1.110 (0.873, 1.412) | 0.3953 |  |
|  |  | 330 | MVMR-Egger | 1.098 (0.859, 1.404) | 0.4539 |  |
|  | HC | 330 | MVMR-IVW | 0.879 (0.717, 1.077) | 0.2136 |  |
|  |  | 330 | MVMR-Egger | 0.871 (0.708, 1.072) | 0.192 |  |
| Peripheral atherosclerosis | BMI | 394 | MVMR-IVW | 1.314 (0.831, 2.078) | 0.2434 | 0.463 |
|  |  | 394 | MVMR-Egger | 1.328 (0.839, 2.104) | 0.2263 |  |
|  | WC | 394 | MVMR-IVW | 3.976 (2.049, 7.714) | 4.46E-05 |  |
|  |  | 394 | MVMR-Egger | 4.233 (2.136, 8.389) | 3.55E-05 |  |
|  | TFP | 394 | MVMR-IVW | 0.955 (0.572, 1.593) | 0.8591 |  |
|  |  | 394 | MVMR-Egger | 0.989 (0.587, 1.666) | 0.9674 |  |
|  | HC | 394 | MVMR-IVW | 0.473 (0.303, 0.738) | 0.001 |  |
|  |  | 394 | MVMR-Egger | 0.487 (0.310, 0.765) | 0.0018 |  |
| Sex hormone binding globulin | BMI | 329 | MVMR-IVW | −0.161 (−0.307, −0.017) | 0.0285 | 0.324 |
|  |  | 329 | MVMR-Egger | −0.189 (−0.343, −0.035) | 0.0166 |  |
|  | WC | 329 | MVMR-IVW | −0.715 (−0.919, −0.513) | 5.00E-12 |  |
|  |  | 329 | MVMR-Egger | −0.709 (−0.911, −0.506) | 8.51E-12 |  |
|  | TFP | 329 | MVMR-IVW | 0.234 (0.091, 0.376) | 0.0014 |  |
|  |  | 329 | MVMR-Egger | 0.219 (0.072, 0.364) | 0.0034 |  |
|  | HC | 329 | MVMR-IVW | 0.337 (0.217, 0.458) | 4.34E-08 |  |
|  |  | 329 | MVMR-Egger | 0.327 (0.205, 0.449) | 1.63E-07 |  |
| Sleep apnea syndrome | BMI | 367 | MVMR-IVW | 1.635 (1.130, 2.367) | 0.0091 | 0.648 |
|  |  | 367 | MVMR-Egger | 1.694 (1.136, 2.526) | 0.0097 |  |
|  | WC | 367 | MVMR-IVW | 2.178 (1.322, 3.588) | 0.0022 |  |
|  |  | 367 | MVMR-Egger | 2.156 (1.305, 3.560) | 0.0027 |  |
|  | TFP | 367 | MVMR-IVW | 0.723 (0.507, 1.031) | 0.0731 |  |
|  |  | 367 | MVMR-Egger | 0.732 (0.511, 1.048) | 0.0886 |  |
|  | HC | 367 | MVMR-IVW | 0.878 (0.651, 1.184) | 0.3935 |  |
|  |  | 367 | MVMR-Egger | 0.893 (0.656, 1.214) | 0.4699 |  |
| Sleep disorders | BMI | 394 | MVMR-IVW | 1.700 (1.273, 2.270) | 0.0003 | 0.603 |
|  |  | 394 | MVMR-Egger | 1.708 (1.278, 2.283) | 0.0003 |  |
|  | WC | 394 | MVMR-IVW | 1.501 (0.988, 2.279) | 0.0569 |  |
|  |  | 394 | MVMR-Egger | 1.543 (1.003, 2.376) | 0.0487 |  |
|  | TFP | 394 | MVMR-IVW | 0.994 (0.720, 1.372) | 0.9701 |  |
|  |  | 394 | MVMR-Egger | 1.010 (0.727, 1.402) | 0.9538 |  |
|  | HC | 394 | MVMR-IVW | 0.796 (0.601, 1.053) | 0.1096 |  |
|  |  | 394 | MVMR-Egger | 0.806 (0.607, 1.072) | 0.1383 |  |
| Type 2 diabetes | BMI | 157 | MVMR-IVW | 1.430 (0.917, 2.230) | 0.1143 | 0.1 |
|  |  | 157 | MVMR-Egger | 1.460 (0.938, 2.273) | 0.0934 |  |
|  | WC | 157 | MVMR-IVW | 1.830 (0.992, 3.376) | 0.0531 |  |
|  |  | 157 | MVMR-Egger | 2.231 (1.161, 4.287) | 0.016 |  |
|  | TFP | 157 | MVMR-IVW | 0.718 (0.419, 1.231) | 0.2287 |  |
|  |  | 157 | MVMR-Egger | 0.771 (0.448, 1.327) | 0.3482 |  |
|  | HC | 157 | MVMR-IVW | 0.733 (0.483, 1.113) | 0.1445 |  |
|  |  | 157 | MVMR-Egger | 0.798 (0.521, 1.224) | 0.3019 |  |
| Varicose veins | BMI | 394 | MVMR-IVW | 0.839 (0.597, 1.179) | 0.3118 | 0.767 |
|  |  | 394 | MVMR-Egger | 0.842 (0.599, 1.184) | 0.3224 |  |
|  | WC | 394 | MVMR-IVW | 0.831 (0.508, 1.358) | 0.4599 |  |
|  |  | 394 | MVMR-Egger | 0.846 (0.510, 1.406) | 0.5197 |  |
|  | TFP | 394 | MVMR-IVW | 1.258 (0.861, 1.838) | 0.2352 |  |
|  |  | 394 | MVMR-Egger | 1.272 (0.864, 1.871) | 0.2225 |  |
|  | HC | 394 | MVMR-IVW | 1.862 (1.340, 2.588) | 0.0002 |  |
|  |  | 394 | MVMR-Egger | 1.879 (1.345, 2.626) | 0.0002 |  |
| Inguinal or femoral hernia, bilateral | BMI | 394 | MVMR-IVW | 0.648 (0.282, 1.490) | 0.3072 | 0.307 |
|  |  | 394 | MVMR-Egger | 0.666 (0.289, 1.534) | 0.3395 |  |
|  | WC | 394 | MVMR-IVW | 0.377 (0.113, 1.256) | 0.1122 |  |
|  |  | 394 | MVMR-Egger | 0.443 (0.128, 1.530) | 0.1978 |  |
|  | TFP | 394 | MVMR-IVW | 0.874 (0.344, 2.217) | 0.7764 |  |
|  |  | 394 | MVMR-Egger | 0.955 (0.371, 2.462) | 0.9248 |  |
|  | HC | 394 | MVMR-IVW | 1.889 (0.843, 4.233) | 0.1223 |  |
|  |  | 394 | MVMR-Egger | 2.036 (0.897, 4.621) | 0.089 |  |
| Rheumatoid arthritis | BMI | 394 | MVMR-IVW | 0.954 (0.606, 1.502) | 0.8376 | 0.757 |
|  |  | 394 | MVMR-Egger | 0.949 (0.602-1.496) | 0.8226 |  |
|  | WC | 394 | MVMR-IVW | 1.309 (0.678, 2.525) | 0.4224 |  |
|  |  | 394 | MVMR-Egger | 1.275 (0.647, 2.510) | 0.4826 |  |
|  | TFP | 394 | MVMR-IVW | 1.287 (0.775, 2.138) | 0.3291 |  |
|  |  | 394 | MVMR-Egger | 1.268 (0.757, 2.125) | 0.3663 |  |
|  | HC | 394 | MVMR-IVW | 1.045 (0.673, 1.623) | 0.844 |  |
|  |  | 394 | MVMR-Egger | 1.032 (0.660, 1.614) | 0.8893 |  |
| Sepsis | BMI | 386 | MVMR-IVW | 0.942 (0.704, 1.260) | 0.6863 | 0.931 |
|  |  | 386 | MVMR-Egger | 0.941 (0.703, 1.260) | 0.6841 |  |
|  | WC | 386 | MVMR-IVW | 2.086 (1.363, 3.193) | 0.0007 |  |
|  |  | 386 | MVMR-Egger | 2.076 (1.339, 3.219) | 0.0011 |  |
|  | TFP | 386 | MVMR-IVW | 0.782 (0.563, 1.084) | 0.1398 |  |
|  |  | 386 | MVMR-Egger | 0.780 (0.559, 1.087) | 0.1423 |  |
|  | HC | 386 | MVMR-IVW | 1.035 (0.778, 1.378) | 0.8122 |  |
|  |  | 386 | MVMR-Egger | 1.032 (0.771, 1.383) | 0.8305 |  |
| Serum uric acid | BMI | 367 | MVMR-IVW | 0.131 (0.030, 0.233) | 0.0111 | 0.236 |
|  |  | 367 | MVMR-Egger | 0.155 (0.047, 0.264) | 0.0051 |  |
|  | WC | 367 | MVMR-IVW | 0.280 (0.142, 0.417) | 0.0001 |  |
|  |  | 367 | MVMR-Egger | 0.272 (0.135, 0.411) | 0.0001 |  |
|  | TFP | 367 | MVMR-IVW | −0.012 (−0.109, 0.084) | 0.8037 |  |
|  |  | 367 | MVMR-Egger | −0.003 (−0.101, 0.095) | 0.9585 |  |
|  | HC | 367 | MVMR-IVW | −0.136 (−0.218, −0.053) | 0.0012 |  |
|  |  | 367 | MVMR-Egger | −0.125 (−0.208, −0.040) | 0.004 |  |

Note: *p-value from MVMR-Egger pleiotropy test. Statistical significance was defined as p < 0.05. Effect size is presented as odds ratio (OR) for binary outcomes and as beta coefficient (β) for continuous outcomes (insulin resistance, sex hormone binding globin, and serum uric acid). All effect sizes correspond to a 1‑standard deviation (SD) increase in the exposure.

Abbreviations: BMI, body mass index; HC, hip circumference; WC, waist circumference; MVMR-IVW, multivariable Mendelian randomization-inverse variance weighted; TFP, total fat percentage.
