# Supplementary material for: Unraveling the causal web of 4 adiposity indices and 92 multi-system outcomes: A body-wide Mendelian randomization study
Source: Medicine (Baltimore). 2026 May 22;105(21):e48986. doi: 10.1097/MD.0000000000048986 (PMC13201005; doi:10.1097/MD.0000000000048986)
Supplement: Supplementary file 8 [file medi-105-e48986-s008.docx]

Table S8. Independent causal effects of adiposity indices on outcomes after adjusting for adipokines in multivariate Mendelian randomization analyses.

| **Outcomes** | **Exposures** | **nSNP** | **Method** | **Effect size (95%CI)** | **p-value** | **Pleiotropy test*** |
| --- | --- | --- | --- | --- | --- | --- |
| Chronic obstructive pulmonary disease | BMI | 112 | MVMR-IVW | 2.017 (1.667, 2.441) | 5.67E-13 | 0.081 |
|  |  | 112 | MVMR-Egger | 2.081 (1.716, 2.522) | 8.80E-14 |  |
|  | Leptin | 112 | MVMR-IVW | 0.684 (0.454, 1.031) | 0.0696 |  |
|  |  | 112 | MVMR-Egger | 0.471 (0.266, 0.835) | 0.0099 |  |
|  | Resistin | 112 | MVMR-IVW | 0.968 (0.874, 1.073) | 0.537 |  |
|  |  | 112 | MVMR-Egger | 0.955 (0.861, 1.059) | 0.3805 |  |
|  | MCP-1 | 112 | MVMR-IVW | 0.971 (0.910, 1.036) | 0.3752 |  |
|  |  | 112 | MVMR-Egger | 0.965 (0.905, 1.030) | 0.2862 |  |
| Gastroesophageal reflux disease | BMI | 108 | MVMR-IVW | 2.036 (1.811, 2.289) | 1.20E-32 | 0.32 |
|  |  | 108 | MVMR-Egger | 2.067 (1.835, 2.329) | 5.49E-33 |  |
|  | Leptin | 108 | MVMR-IVW | 1.027 (0.795, 1.326) | 0.8396 |  |
|  |  | 108 | MVMR-Egger | 0.891 (0.622, 1.277) | 0.5301 |  |
|  | Resistin | 108 | MVMR-IVW | 0.976 (0.916, 1.039) | 0.4446 |  |
|  |  | 108 | MVMR-Egger | 0.969 (0.909, 1.033) | 0.3305 |  |
|  | MCP-1 | 108 | MVMR-IVW | 1.026 (0.986, 1.067) | 0.2103 |  |
|  |  | 108 | MVMR-Egger | 1.023 (0.983, 1.065) | 0.2639 |  |
| Hypertension | BMI | 162 | MVMR-IVW | 2.008 (1.728, 2.333) | 8.80E-20 | 0.997 |
|  |  | 162 | MVMR-Egger | 2.009 (1.443, 2.797) | 3.56E-05 |  |
|  | Leptin | 162 | MVMR-IVW | 0.829 (0.621, 1.106) | 0.2024 |  |
|  |  | 162 | MVMR-Egger | 0.828 (0.617, 1.113) | 0.2112 |  |
|  | Resistin | 162 | MVMR-IVW | 0.991 (0.922, 1.064) | 0.7975 |  |
|  |  | 162 | MVMR-Egger | 0.991 (0.922, 1.065) | 0.7982 |  |
|  | MCP-1 | 162 | MVMR-IVW | 0.989 (0.947, 1.033) | 0.6303 |  |
|  |  | 162 | MVMR-Egger | 0.989 (0.947, 1.034) | 0.6316 |  |
| Osteoarthritis | BMI | 112 | MVMR-IVW | 1.638 (1.425, 1.882) | 3.32E-12 | 0.731 |
|  |  | 112 | MVMR-Egger | 1.632 (1.420, 1.875) | 4.80E-12 |  |
|  | Leptin | 112 | MVMR-IVW | 0.984 (0.728, 1.328) | 0.9139 |  |
|  |  | 112 | MVMR-Egger | 1.036 (0.684, 1.568) | 0.8684 |  |
|  | Resistin | 112 | MVMR-IVW | 0.949 (0.880, 1.022) | 0.1662 |  |
|  |  | 112 | MVMR-Egger | 0.957 (0.888, 1.031) | 0.2457 |  |
|  | MCP-1 | 112 | MVMR-IVW | 1.024 (0.977, 1.073) | 0.3302 |  |
|  |  | 112 | MVMR-Egger | 1.029 (0.982, 1.078) | 0.2275 |  |
| Serum uric acid | BMI | 112 | MVMR-IVW | 0.252 (0.208, 0.297) | 8.35E-30 | 0.031 |
|  |  | 112 | MVMR-Egger | 0.248 (0.204, 0.291) | 1.12E-28 |  |
|  | Leptin | 112 | MVMR-IVW | –0.008 (–0.102, 0.086) | 0.8708 |  |
|  |  | 112 | MVMR-Egger | 0.092 (–0.038, 0.222) | 0.165 |  |
|  | Resistin | 112 | MVMR-IVW | 0.005 (–0.018, 0.029) | 0.6473 |  |
|  |  | 112 | MVMR-Egger | 0.007 (–0.017, 0.030) | 0.575 |  |
|  | MCP-1 | 112 | MVMR-IVW | –0.009 (–0.024, 0.005) | 0.2061 |  |
|  |  | 112 | MVMR-Egger | –0.009 (–0.024, 0.005) | 0.2113 |  |
| Sleep apnea syndrome | BMI | 112 | MVMR-IVW | 2.321 (1.889, 2.852) | 1.09E-15 | 0.353 |
|  |  | 112 | MVMR-Egger | 2.270 (1.840, 2.801) | 2.15E-14 |  |
|  | Leptin | 112 | MVMR-IVW | 1.170 (0.752, 1.822) | 0.4859 |  |
|  |  | 112 | MVMR-Egger | 1.465 (0.783, 2.742) | 0.2327 |  |
|  | Resistin | 112 | MVMR-IVW | 0.973 (0.869, 1.089) | 0.6333 |  |
|  |  | 112 | MVMR-Egger | 0.983 (0.876, 1.104) | 0.7746 |  |
|  | MCP-1 | 112 | MVMR-IVW | 0.923 (0.860, 0.991) | 0.0277 |  |
|  |  | 112 | MVMR-Egger | 0.928 (0.864, 0.997) | 0.0415 |  |
| Sleep disorders | BMI | 162 | MVMR-IVW | 1.772 (1.477, 2.127) | 7.63E-10 | 0.988 |
|  |  | 162 | MVMR-Egger | 1.777 (1.189, 2.655) | 0.005 |  |
|  | Leptin | 162 | MVMR-IVW | 1.049 (0.738, 1.491) | 0.7879 |  |
|  |  | 162 | MVMR-Egger | 1.049 (0.733, 1.501) | 0.7942 |  |
|  | Resistin | 162 | MVMR-IVW | 1.024 (0.939, 1.117) | 0.5937 |  |
|  |  | 162 | MVMR-Egger | 1.024 (0.938, 1.118) | 0.5955 |  |
|  | MCP-1 | 162 | MVMR-IVW | 0.976 (0.926, 1.029) | 0.3728 |  |
|  |  | 162 | MVMR-Egger | 0.976 (0.926, 1.030) | 0.3745 |  |
| Asthma | WC | 86 | MVMR-IVW | 1.422 (1.097, 1.843) | 0.0078 | 0.882 |
|  |  | 86 | MVMR-Egger | 1.403 (1.076, 1.831) | 0.0125 |  |
|  | Leptin | 86 | MVMR-IVW | 0.903 (0.582, 1.402) | 0.6505 |  |
|  |  | 86 | MVMR-Egger | 0.899 (0.538, 1.504) | 0.6856 |  |
|  | Resistin | 86 | MVMR-IVW | 1.150 (1.043, 1.269) | 0.0052 |  |
|  |  | 86 | MVMR-Egger | 1.156 (1.045, 1.279) | 0.005 |  |
|  | MCP-1 | 86 | MVMR-IVW | 1.030 (0.960, 1.106) | 0.4071 |  |
|  |  | 86 | MVMR-Egger | 1.026 (0.955, 1.103) | 0.4826 |  |
| Atrial fibrillation and flutter | WC | 100 | MVMR-IVW | 1.910 (1.404, 2.598) | 3.81E-05 | 0.808 |
|  |  | 100 | MVMR-Egger | 1.789 (0.974, 3.288) | 0.0608 |  |
|  | Leptin | 100 | MVMR-IVW | 1.179 (0.705, 1.972) | 0.5292 |  |
|  |  | 100 | MVMR-Egger | 1.190 (0.706, 2.004) | 0.5134 |  |
|  | Resistin | 100 | MVMR-IVW | 1.118 (0.980, 1.276) | 0.0979 |  |
|  |  | 100 | MVMR-Egger | 1.120 (0.980, 1.280) | 0.0963 |  |
|  | MCP-1 | 100 | MVMR-IVW | 0.910 (0.838, 0.987) | 0.0235 |  |
|  |  | 100 | MVMR-Egger | 0.911 (0.839, 0.990) | 0.0279 |  |
| Cholecystitis | WC | 100 | MVMR-IVW | 1.885 (1.420, 2.504) | 1.18E-05 | 0.102 |
|  |  | 100 | MVMR-Egger | 2.805 (1.614, 4.876) | 0.0003 |  |
|  | Leptin | 100 | MVMR-IVW | 0.881 (0.549, 1.414) | 0.6005 |  |
|  |  | 100 | MVMR-Egger | 0.835 (0.520, 1.341) | 0.455 |  |
|  | Resistin | 100 | MVMR-IVW | 0.986 (0.873, 1.114) | 0.8215 |  |
|  |  | 100 | MVMR-Egger | 0.976 (0.865, 1.102) | 0.6961 |  |
|  | MCP-1 | 100 | MVMR-IVW | 1.001 (0.928, 1.080) | 0.9761 |  |
|  |  | 100 | MVMR-Egger | 0.992 (0.920, 1.070) | 0.8424 |  |
| Cholelithiasis | WC | 100 | MVMR-IVW | 1.935 (1.455, 2.574) | 5.77E-06 | 0.098 |
|  |  | 100 | MVMR-Egger | 2.897 (1.662, 5.048) | 0.0002 |  |
|  | Leptin | 100 | MVMR-IVW | 0.869 (0.540, 1.398) | 0.5617 |  |
|  |  | 100 | MVMR-Egger | 0.822 (0.511, 1.323) | 0.4195 |  |
|  | Resistin | 100 | MVMR-IVW | 0.986 (0.872, 1.114) | 0.8151 |  |
|  |  | 100 | MVMR-Egger | 0.975 (0.863, 1.102) | 0.6888 |  |
|  | MCP-1 | 100 | MVMR-IVW | 1.012 (0.938, 1.091) | 0.7659 |  |
|  |  | 100 | MVMR-Egger | 1.003 (0.929, 1.082) | 0.9465 |  |
| Gestational diabetes | WC | 100 | MVMR-IVW | 1.712 (1.187, 2.469) | 0.004 | 0.094 |
|  |  | 100 | MVMR-Egger | 2.906 (1.414, 5.970) | 0.0037 |  |
|  | Leptin | 100 | MVMR-IVW | 1.270 (0.689, 2.339) | 0.4438 |  |
|  |  | 100 | MVMR-Egger | 1.182 (0.638, 2.190) | 0.5947 |  |
|  | Resistin | 100 | MVMR-IVW | 0.911 (0.779, 1.066) | 0.2464 |  |
|  |  | 100 | MVMR-Egger | 0.899 (0.768, 1.053) | 0.1861 |  |
|  | MCP-1 | 100 | MVMR-IVW | 1.004 (0.911, 1.107) | 0.9305 |  |
|  |  | 100 | MVMR-Egger | 0.993 (0.900, 1.095) | 0.8813 |  |
| Heart failure | WC | 100 | MVMR-IVW | 1.964 (1.471, 2.623) | 4.68E-06 | 0.654 |
|  |  | 100 | MVMR-Egger | 2.198 (1.242, 3.891) | 0.0068 |  |
|  | Leptin | 100 | MVMR-IVW | 1.076 (0.665, 1.744) | 0.7645 |  |
|  |  | 100 | MVMR-Egger | 1.060 (0.650, 1.729) | 0.8141 |  |
|  | Resistin | 100 | MVMR-IVW | 1.041 (0.920, 1.179) | 0.5232 |  |
|  |  | 100 | MVMR-Egger | 1.038 (0.916, 1.177) | 0.557 |  |
|  | MCP-1 | 100 | MVMR-IVW | 0.955 (0.885, 1.032) | 0.2456 |  |
|  |  | 100 | MVMR-Egger | 0.953 (0.882, 1.030) | 0.2271 |  |
| Hypertension | WC | 100 | MVMR-IVW | 1.821 (1.399, 2.370) | 8.40E-06 | 0.47 |
|  |  | 100 | MVMR-Egger | 2.147 (1.277, 3.610) | 0.0039 |  |
|  | Leptin | 100 | MVMR-IVW | 1.178 (0.759, 1.829) | 0.4645 |  |
|  |  | 100 | MVMR-Egger | 1.152 (0.738, 1.798) | 0.5327 |  |
|  | Resistin | 100 | MVMR-IVW | 0.987 (0.881, 1.105) | 0.8146 |  |
|  |  | 100 | MVMR-Egger | 0.982 (0.877, 1.101) | 0.7604 |  |
|  | MCP-1 | 100 | MVMR-IVW | 0.959 (0.894, 1.028) | 0.2384 |  |
|  |  | 100 | MVMR-Egger | 0.955 (0.890, 1.025) | 0.2058 |  |
| Infections of the skin and subcutaneous tissue | WC | 100 | MVMR-IVW | 1.621 (1.240, 2.119) | 0.0004 | 0.727 |
|  |  | 100 | MVMR-Egger | 1.758 (1.038, 2.976) | 0.0358 |  |
|  | Leptin | 100 | MVMR-IVW | 0.765 (0.489, 1.196) | 0.2394 |  |
|  |  | 100 | MVMR-Egger | 0.756 (0.482, 1.187) | 0.2251 |  |
|  | Resistin | 100 | MVMR-IVW | 1.107 (0.987, 1.242) | 0.082 |  |
|  |  | 100 | MVMR-Egger | 1.105 (0.985, 1.240) | 0.09 |  |
|  | MCP-1 | 100 | MVMR-IVW | 1.003 (0.934, 1.077) | 0.9325 |  |
|  |  | 100 | MVMR-Egger | 1.001 (0.932, 1.076) | 0.9721 |  |
| Peripheral atherosclerosis | WC | 100 | MVMR-IVW | 2.218 (1.503, 3.273) | 0.0001 | 0.64 |
|  |  | 100 | MVMR-Egger | 2.597 (1.205, 5.600) | 0.0149 |  |
|  | Leptin | 100 | MVMR-IVW | 0.672 (0.351, 1.285) | 0.2295 |  |
|  |  | 100 | MVMR-Egger | 0.658 (0.341, 1.269) | 0.2116 |  |
|  | Resistin | 100 | MVMR-IVW | 1.078 (0.912, 1.274) | 0.3782 |  |
|  |  | 100 | MVMR-Egger | 1.074 (0.907, 1.271) | 0.4087 |  |
|  | MCP-1 | 100 | MVMR-IVW | 1.018 (0.918, 1.129) | 0.7313 |  |
|  |  | 100 | MVMR-Egger | 1.015 (0.914, 1.127) | 0.7848 |  |
| Sleep apnea syndrome | WC | 86 | MVMR-IVW | 2.506 (1.848, 3.398) | 3.38E-09 | 0.874 |
|  |  | 86 | MVMR-Egger | 2.475 (1.815, 3.374) | 1.02E-08 |  |
|  | Leptin | 86 | MVMR-IVW | 1.003 (0.600, 1.679) | 0.9898 |  |
|  |  | 86 | MVMR-Egger | 1.057 (0.579, 1.929) | 0.8576 |  |
|  | Resistin | 86 | MVMR-IVW | 1.043 (0.917, 1.185) | 0.5242 |  |
|  |  | 86 | MVMR-Egger | 1.048 (0.919, 1.196) | 0.4811 |  |
|  | MCP-1 | 86 | MVMR-IVW | 1.002 (0.923, 1.089) | 0.9563 |  |
|  |  | 86 | MVMR-Egger | 0.997 (0.917, 1.084) | 0.9474 |  |
| Serum uric acid | WC | 86 | MVMR-IVW | 0.328 (0.240, 0.416) | 2.51E-13 | 0.712 |
|  |  | 86 | MVMR-Egger | 0.333 (0.243, 0.423) | 4.16E-13 |  |
|  | Leptin | 86 | MVMR-IVW | –0.097 (–0.243, 0.052) | 0.2019 |  |
|  |  | 86 | MVMR-Egger | –0.082 (–0.256, 0.092) | 0.3531 |  |
|  | Resistin | 86 | MVMR-IVW | 0.027 (–0.007, 0.059) | 0.1217 |  |
|  |  | 86 | MVMR-Egger | 0.024 (–0.010, 0.058) | 0.1682 |  |
|  | MCP-1 | 86 | MVMR-IVW | –0.004 (–0.028, 0.020) | 0.7539 |  |
|  |  | 86 | MVMR-Egger | –0.003 (–0.027, 0.022) | 0.8413 |  |
| Gastroesophageal reflux disease | TFP | 86 | MVMR-IVW | 2.133 (1.782, 2.552) | 1.33E-16 | 0.003 |
|  |  | 86 | MVMR-Egger | 1.967 (1.642, 2.355) | 1.88E-13 |  |
|  | Leptin | 86 | MVMR-IVW | 1.090 (0.809, 1.468) | 0.5711 |  |
|  |  | 86 | MVMR-Egger | 0.799 (0.561, 1.137) | 0.2117 |  |
|  | Resistin | 86 | MVMR-IVW | 1.017 (0.947, 1.092) | 0.6502 |  |
|  |  | 86 | MVMR-Egger | 1.046 (0.975, 1.123) | 0.2108 |  |
|  | MCP-1 | 86 | MVMR-IVW | 1.022 (0.973, 1.073) | 0.3942 |  |
|  |  | 86 | MVMR-Egger | 1.018 (0.971, 1.067) | 0.4708 |  |
| Hypothyroidism | HC | 103 | MVMR-IVW | 1.295 (1.066, 1.573) | 0.0091 | 0.536 |
|  |  | 103 | MVMR-Egger | 1.233 (1.009, 1.506) | 0.0402 |  |
|  | Leptin | 103 | MVMR-IVW | 1.055 (0.710, 1.565) | 0.7919 |  |
|  |  | 103 | MVMR-Egger | 1.016 (0.637, 1.621) | 0.9474 |  |
|  | Resistin | 103 | MVMR-IVW | 1.069 (0.966, 1.182) | 0.1948 |  |
|  |  | 103 | MVMR-Egger | 1.084 (0.981, 1.198) | 0.1149 |  |
|  | MCP-1 | 103 | MVMR-IVW | 1.019 (0.959, 1.084) | 0.5452 |  |
|  |  | 103 | MVMR-Egger | 1.010 (0.950, 1.073) | 0.7479 |  |
| Varicose veins | HC | 123 | MVMR-IVW | 1.661 (1.369, 2.015) | 2.67E-07 | 0.138 |
|  |  | 123 | MVMR-Egger | 2.205 (1.447, 3.360) | 0.0002 |  |
|  | Leptin | 123 | MVMR-IVW | 1.008 (0.694, 1.464) | 0.9658 |  |
|  |  | 123 | MVMR-Egger | 0.974 (0.670, 1.416) | 0.8911 |  |
|  | Resistin | 123 | MVMR-IVW | 1.092 (0.987, 1.208) | 0.0867 |  |
|  |  | 123 | MVMR-Egger | 1.087 (0.983, 1.202) | 0.1023 |  |
|  | MCP-1 | 123 | MVMR-IVW | 1.086 (1.019, 1.158) | 0.0111 |  |
|  |  | 123 | MVMR-Egger | 1.076 (1.009, 1.148) | 0.0265 |  |
| Acute pancreatitis | HC | 103 | MVMR-IVW | 1.323 (0.983, 1.782) | 0.0651 | 0.389 |
|  |  | 103 | MVMR-Egger | 1.269 (0.929, 1.734) | 0.1345 |  |
|  | Resistin | 103 | MVMR-IVW | 0.962 (0.824, 1.124) | 0.6299 |  |
|  |  | 103 | MVMR-Egger | 0.966 (0.826, 1.130) | 0.6687 |  |
|  | MCP-1 | 103 | MVMR-IVW | 0.971 (0.884, 1.068) | 0.547 |  |
|  |  | 103 | MVMR-Egger | 0.968 (0.880, 1.065) | 0.5094 |  |
|  | Leptin | 103 | MVMR-IVW | 0.750 (0.408, 1.378) | 0.3539 |  |
|  |  | 103 | MVMR-Egger | 0.635 (0.305, 1.318) | 0.2228 |  |
| Gastroesophageal reflux disease | HC | 101 | MVMR-IVW | 1.510 (1.302, 1.750) | 4.69E-08 |  |
|  |  | 101 | MVMR-Egger | 1.532 (1.315, 1.785) | 4.56E-08 |  |
|  | Resistin | 101 | MVMR-IVW | 0.983 (0.912, 1.061) | 0.6667 |  |
|  |  | 101 | MVMR-Egger | 0.976 (0.905, 1.053) | 0.5322 |  |
|  | MCP-1 | 101 | MVMR-IVW | 0.980 (0.936, 1.026) | 0.389 |  |
|  |  | 101 | MVMR-Egger | 0.984 (0.940, 1.031) | 0.5038 |  |
|  | Leptin | 101 | MVMR-IVW | 1.475 (1.094, 1.988) | 0.0108 |  |
|  |  | 101 | MVMR-Egger | 1.390 (0.970, 1.992) | 0.0727 |  |
| Gestational diabetes | HC | 123 | MVMR-IVW | 1.407 (1.028, 1.926) | 0.033 | 0.879 |
|  |  | 123 | MVMR-Egger | 1.476 (0.740, 2.942) | 0.2693 |  |
|  | Leptin | 123 | MVMR-IVW | 1.588 (0.866, 2.913) | 0.1347 |  |
|  |  | 123 | MVMR-Egger | 1.579 (0.855, 2.916) | 0.1441 |  |
|  | Resistin | 123 | MVMR-IVW | 0.965 (0.819, 1.137) | 0.6681 |  |
|  |  | 123 | MVMR-Egger | 0.964 (0.818, 1.137) | 0.6636 |  |
|  | MCP-1 | 123 | MVMR-IVW | 1.044 (0.941, 1.159) | 0.4137 |  |
|  |  | 123 | MVMR-Egger | 1.043 (0.937, 1.160) | 0.4418 |  |
| Hypertension | HC | 123 | MVMR-IVW | 1.507 (1.273, 1.784) | 1.89E-06 | 0.249 |
|  |  | 123 | MVMR-Egger | 1.828 (1.264, 2.645) | 0.0014 |  |
|  | Leptin | 123 | MVMR-IVW | 1.164 (0.840, 1.612) | 0.3619 |  |
|  |  | 123 | MVMR-Egger | 1.137 (0.819, 1.578) | 0.4434 |  |
|  | Resistin | 123 | MVMR-IVW | 0.986 (0.903, 1.077) | 0.7527 |  |
|  |  | 123 | MVMR-Egger | 0.983 (0.900, 1.074) | 0.7036 |  |
|  | MCP-1 | 123 | MVMR-IVW | 0.955 (0.903, 1.010) | 0.1062 |  |
|  |  | 123 | MVMR-Egger | 0.949 (0.896, 1.004) | 0.0703 |  |
| Insulin resistance | HC | 102 | MVMR-IVW | 0.112 (0.049, 0.175) | 0.0005 | 0.221 |
|  |  | 102 | MVMR-Egger | 0.135 (0.069, 0.201) | 0.0001 |  |
|  | Resistin | 102 | MVMR-IVW | 0.024 (–0.008, 0.055) | 0.1418 |  |
|  |  | 102 | MVMR-Egger | 0.020 (–0.011, 0.051) | 0.2186 |  |
|  | MCP-1 | 102 | MVMR-IVW | 0.007 (–0.012, 0.026) | 0.4865 |  |
|  |  | 102 | MVMR-Egger | 0.010 (–0.009, 0.029) | 0.3221 |  |
|  | Leptin | 102 | MVMR-IVW | 0.070 (–0.058, 0.199) | 0.2794 |  |
|  |  | 102 | MVMR-Egger | 0.103 (–0.044, 0.251) | 0.1687 |  |
| Peripheral atherosclerosis | HC | 123 | MVMR-IVW | 1.310 (0.984, 1.744) | 0.0642 | 0.147 |
|  |  | 123 | MVMR-Egger | 1.976 (1.059, 3.690) | 0.0325 |  |
|  | Leptin | 123 | MVMR-IVW | 1.359 (0.782, 2.362) | 0.2765 |  |
|  |  | 123 | MVMR-Egger | 1.294 (0.743, 2.252) | 0.3627 |  |
|  | Resistin | 123 | MVMR-IVW | 1.011 (0.871, 1.174) | 0.8875 |  |
|  |  | 123 | MVMR-Egger | 1.004 (0.865, 1.166) | 0.9535 |  |
|  | MCP-1 | 123 | MVMR-IVW | 0.986 (0.897, 1.084) | 0.7689 |  |
|  |  | 123 | MVMR-Egger | 0.972 (0.883, 1.071) | 0.5691 |  |
| Serum uric acid | HC | 103 | MVMR-IVW | 0.175 (0.116, 0.234) | 5.37E-09 | 0.962 |
|  |  | 103 | MVMR-Egger | 0.177 (0.115, 0.239) | 1.94E-08 |  |
|  | Resistin | 103 | MVMR-IVW | 0.027 (–0.003, 0.057) | 0.0811 |  |
|  |  | 103 | MVMR-Egger | 0.026 (–0.005, 0.056) | 0.0974 |  |
|  | MCP-1 | 103 | MVMR-IVW | 0.006 (–0.013, 0.025) | 0.5376 |  |
|  |  | 103 | MVMR-Egger | 0.007 (–0.012, 0.026) | 0.498 |  |
|  | Leptin | 103 | MVMR-IVW | 0.071 (–0.047, 0.190) | 0.24 |  |
|  |  | 103 | MVMR-Egger | 0.066 (–0.078, 0.210) | 0.369 |  |

Note: *p-value from MVMR-Egger pleiotropy test. Statistical significance was defined as p < 0.05. Effect size is presented as odds ratio (OR) for binary outcomes and as beta coefficient (β) for continuous outcomes (serum uric acid and insulin resistance). All effect sizes correspond to a 1‑standard deviation (SD) increase in the exposure.

Abbreviations: BMI, body mass index; HC, hip circumference; WC, waist circumference; MCP-1, monocyte chemoattractant protein-1; MVMR-IVW, multivariable Mendelian randomization-inverse variance weighted; TFP, total fat percentage.
