# Supplementary material for: Unraveling the causal web of 4 adiposity indices and 92 multi-system outcomes: A body-wide Mendelian randomization study
Source: Medicine (Baltimore). 2026 May 22;105(21):e48986. doi: 10.1097/MD.0000000000048986 (PMC13201005; doi:10.1097/MD.0000000000048986)
Supplement: Supplementary file 9 [file medi-105-e48986-s009.docx]

Table S9. Independent causal effects of adiposity indices on outcomes after adjusting for inflammatory markers in multivariate Mendelian randomization analyses.

| **Outcome** | **Exposure** | **nSNP** | **Method** | Effect size (95%CI) | **p-value** | **Pleiotropy test*** |
| --- | --- | --- | --- | --- | --- | --- |
| Chronic obstructive pulmonary disease | BMI | 129 | MVMR-IVW | 1.764 (1.524, 2.041) | 2.77E-14 | 0.767 |
|  |  | 129 | MVMR-Egger | 1.736 (1.449, 2.079) | 2.18E-09 |  |
|  | C-reactive protein | 129 | MVMR-IVW | 1.063 (0.976, 1.159) | 0.1608 |  |
|  |  | 129 | MVMR-Egger | 1.057 (0.959, 1.164) | 0.2633 |  |
|  | TNF-α | 129 | MVMR-IVW | 0.980 (0.920, 1.042) | 0.515 |  |
|  |  | 129 | MVMR-Egger | 0.981 (0.921, 1.044) | 0.5401 |  |
|  | IL-6 | 129 | MVMR-IVW | 1.009 (0.955, 1.066) | 0.748 |  |
|  |  | 129 | MVMR-Egger | 1.010 (0.955, 1.067) | 0.7382 |  |
| Gastroesophageal reflux disease | BMI | 125 | MVMR-IVW | 2.047 (1.894, 2.213) | 7.46E-73 | 0.107 |
|  |  | 125 | MVMR-Egger | 1.955 (1.777, 2.151) | 4.70E-43 |  |
|  | C-reactive protein | 125 | MVMR-IVW | 1.007 (0.962, 1.055) | 0.7544 |  |
|  |  | 125 | MVMR-Egger | 0.989 (0.939, 1.041) | 0.6686 |  |
|  | TNF-α | 125 | MVMR-IVW | 0.984 (0.952, 1.018) | 0.3592 |  |
|  |  | 125 | MVMR-Egger | 0.987 (0.954, 1.021) | 0.4435 |  |
|  | IL-6 | 125 | MVMR-IVW | 1.002 (0.972, 1.032) | 0.9154 |  |
|  |  | 125 | MVMR-Egger | 1.002 (0.973, 1.032) | 0.8726 |  |
| Hypertension | BMI | 170 | MVMR-IVW | 1.857 (1.637, 2.106) | 6.25E-22 | 0.845 |
|  |  | 170 | MVMR-Egger | 1.811 (1.365, 2.402) | 3.83E-05 |  |
|  | C-reactive protein | 170 | MVMR-IVW | 1.020 (0.950, 1.096) | 0.5807 |  |
|  |  | 170 | MVMR-Egger | 1.022 (0.950, 1.099) | 0.5639 |  |
|  | TNF-α | 170 | MVMR-IVW | 0.985 (0.944, 1.029) | 0.5053 |  |
|  |  | 170 | MVMR-Egger | 0.986 (0.944, 1.030) | 0.5192 |  |
|  | IL-6 | 170 | MVMR-IVW | 1.004 (0.962, 1.047) | 0.8709 |  |
|  |  | 170 | MVMR-Egger | 1.003 (0.962, 1.047) | 0.873 |  |
| Osteoarthritis | BMI | 121 | MVMR-IVW | 1.664 (1.505, 1.840) | 3.25E-23 | 0.859 |
|  |  | 121 | MVMR-Egger | 1.676 (1.477, 1.901) | 1.09E-15 |  |
|  | C-reactive protein | 121 | MVMR-IVW | 1.014 (0.956, 1.075) | 0.6413 |  |
|  |  | 121 | MVMR-Egger | 1.017 (0.952, 1.086) | 0.6213 |  |
|  | TNF-α | 121 | MVMR-IVW | 0.971 (0.931, 1.013) | 0.1793 |  |
|  |  | 121 | MVMR-Egger | 0.971 (0.930, 1.014) | 0.1776 |  |
|  | IL-6 | 121 | MVMR-IVW | 0.970 (0.933, 1.008) | 0.1222 |  |
|  |  | 121 | MVMR-Egger | 0.970 (0.933, 1.008) | 0.1222 |  |
| Serum uric acid | BMI | 129 | MVMR-IVW | 0.206 (0.148, 0.263) | 1.68E-12 | 0.226 |
|  |  | 129 | MVMR-Egger | 0.181 (0.111, 0.251) | 4.83E-07 |  |
|  | C-reactive protein | 129 | MVMR-IVW | 0.071 (0.037, 0.105) | 4.50E-05 |  |
|  |  | 129 | MVMR-Egger | 0.061 (0.023, 0.099) | 0.0018 |  |
|  | TNF-α | 129 | MVMR-IVW | −0.002 (−0.026, 0.023) | 0.8627 |  |
|  |  | 129 | MVMR-Egger | −0.001 (−0.025, 0.024) | 0.9617 |  |
|  | IL-6 | 129 | MVMR-IVW | 0.015 (−0.007, 0.036) | 0.1821 |  |
|  |  | 129 | MVMR-Egger | 0.015 (−0.006, 0.036) | 0.166 |  |
| Sleep apnea syndrome | BMI | 129 | MVMR-IVW | 2.257 (1.930, 2.639) | 1.92E-24 | 0.795 |
|  |  | 129 | MVMR-Egger | 2.224 (1.834, 2.697) | 4.45E-16 |  |
|  | C-reactive protein | 129 | MVMR-IVW | 1.016 (0.929, 1.111) | 0.7287 |  |
|  |  | 129 | MVMR-Egger | 1.010 (0.913, 1.117) | 0.8466 |  |
|  | TNF-α | 129 | MVMR-IVW | 1.006 (0.942, 1.074) | 0.855 |  |
|  |  | 129 | MVMR-Egger | 1.007 (0.943, 1.076) | 0.8318 |  |
|  | IL-6 | 129 | MVMR-IVW | 0.957 (0.902, 1.015) | 0.144 |  |
|  |  | 129 | MVMR-Egger | 0.957 (0.902, 1.016) | 0.1506 |  |
| Sleep disorders | BMI | 170 | MVMR-IVW | 1.802 (1.561, 2.080) | 9.21E-16 | 0.872 |
|  |  | 170 | MVMR-Egger | 1.760 (1.275, 2.428) | 0.0006 |  |
|  | C-reactive protein | 170 | MVMR-IVW | 1.010 (0.931, 1.095) | 0.8132 |  |
|  |  | 170 | MVMR-Egger | 1.011 (0.931, 1.098) | 0.794 |  |
|  | TNF-α | 170 | MVMR-IVW | 1.001 (0.953, 1.051) | 0.9778 |  |
|  |  | 170 | MVMR-Egger | 1.001 (0.953, 1.052) | 0.9667 |  |
|  | IL-6 | 170 | MVMR-IVW | 0.997 (0.950, 1.046) | 0.8902 |  |
|  |  | 170 | MVMR-Egger | 0.997 (0.949, 1.046) | 0.8891 |  |
| Asthma | WC | 96 | MVMR-IVW | 1.392 (1.175, 1.649) | 0.0001 | 0.221 |
|  |  | 96 | MVMR-Egger | 1.520 (1.220, 1.894) | 0.0002 |  |
|  | C-reactive protein | 96 | MVMR-IVW | 1.027 (0.953, 1.106) | 0.4867 |  |
|  |  | 96 | MVMR-Egger | 1.054 (0.968, 1.147) | 0.2274 |  |
|  | TNF-α | 96 | MVMR-IVW | 0.971 (0.918, 1.028) | 0.3155 |  |
|  |  | 96 | MVMR-Egger | 0.971 (0.917, 1.027) | 0.3036 |  |
|  | IL-6 | 96 | MVMR-IVW | 1.026 (0.972, 1.084) | 0.3473 |  |
|  |  | 96 | MVMR-Egger | 1.025 (0.971, 1.082) | 0.3702 |  |
| Atrial fibrillation and flutter | WC | 112 | MVMR-IVW | 2.035 (1.620, 2.557) | 9.95E-10 | 0.743 |
|  |  | 112 | MVMR-Egger | 1.906 (1.208, 3.007) | 0.0056 |  |
|  | C-reactive protein | 112 | MVMR-IVW | 0.962 (0.870, 1.064) | 0.454 |  |
|  |  | 112 | MVMR-Egger | 0.964 (0.871, 1.067) | 0.4769 |  |
|  | TNF-α | 112 | MVMR-IVW | 0.982 (0.915, 1.054) | 0.611 |  |
|  |  | 112 | MVMR-Egger | 0.982 (0.915, 1.055) | 0.6231 |  |
|  | IL-6 | 112 | MVMR-IVW | 0.978 (0.915, 1.046) | 0.5217 |  |
|  |  | 112 | MVMR-Egger | 0.979 (0.915, 1.047) | 0.5331 |  |
| Cholecystitis | WC | 112 | MVMR-IVW | 1.843 (1.453, 2.339) | 4.89E-07 | 0.043 |
|  |  | 112 | MVMR-Egger | 2.802 (1.754, 4.476) | 1.62E-05 |  |
|  | C-reactive protein | 112 | MVMR-IVW | 1.073 (0.966, 1.193) | 0.1872 |  |
|  |  | 112 | MVMR-Egger | 1.062 (0.957, 1.179) | 0.2555 |  |
|  | TNF-α | 112 | MVMR-IVW | 0.947 (0.879, 1.020) | 0.1476 |  |
|  |  | 112 | MVMR-Egger | 0.944 (0.877, 1.015) | 0.1195 |  |
|  | IL-6 | 112 | MVMR-IVW | 1.055 (0.984, 1.131) | 0.133 |  |
|  |  | 112 | MVMR-Egger | 1.052 (0.982, 1.127) | 0.1514 |  |
| Cholelithiasis | WC | 112 | MVMR-IVW | 1.889 (1.488, 2.398) | 1.73E-07 | 0.041 |
|  |  | 112 | MVMR-Egger | 2.878 (1.802, 4.597) | 9.59E-06 |  |
|  | C-reactive protein | 112 | MVMR-IVW | 1.071 (0.963, 1.190) | 0.2049 |  |
|  |  | 112 | MVMR-Egger | 1.060 (0.954, 1.176) | 0.2773 |  |
|  | TNF-α | 112 | MVMR-IVW | 0.949 (0.881, 1.022) | 0.1672 |  |
|  |  | 112 | MVMR-Egger | 0.946 (0.879, 1.018) | 0.1366 |  |
|  | IL-6 | 112 | MVMR-IVW | 1.056 (0.985, 1.133) | 0.1258 |  |
|  |  | 112 | MVMR-Egger | 1.053 (0.983, 1.128) | 0.1432 |  |
| Gestational diabetes | WC | 112 | MVMR-IVW | 2.111 (1.559, 2.859) | 1.39E-06 | 0.459 |
|  |  | 112 | MVMR-Egger | 2.572 (1.404, 4.714) | 0.0022 |  |
|  | C-reactive protein | 112 | MVMR-IVW | 0.941 (0.823, 1.075) | 0.3711 |  |
|  |  | 112 | MVMR-Egger | 0.936 (0.819, 1.071) | 0.3378 |  |
|  | TNF-α | 112 | MVMR-IVW | 0.955 (0.869, 1.049) | 0.3389 |  |
|  |  | 112 | MVMR-Egger | 0.954 (0.868, 1.048) | 0.3245 |  |
|  | IL-6 | 112 | MVMR-IVW | 0.941 (0.861, 1.029) | 0.1832 |  |
|  |  | 112 | MVMR-Egger | 0.940 (0.860, 1.028) | 0.1743 |  |
| Heart failure | WC | 112 | MVMR-IVW | 2.003 (1.630, 2.462) | 4.15E-11 | 0.94 |
|  |  | 112 | MVMR-Egger | 1.976 (1.307, 2.987) | 0.0012 |  |
|  | C-reactive protein | 112 | MVMR-IVW | 0.998 (0.911, 1.093) | 0.9688 |  |
|  |  | 112 | MVMR-Egger | 0.999 (0.911, 1.095) | 0.9749 |  |
|  | TNF-α | 112 | MVMR-IVW | 0.994 (0.933, 1.060) | 0.8625 |  |
|  |  | 112 | MVMR-Egger | 0.994 (0.932, 1.061) | 0.8658 |  |
|  | IL-6 | 112 | MVMR-IVW | 1.003 (0.944, 1.065) | 0.9266 |  |
|  |  | 112 | MVMR-Egger | 1.003 (0.944, 1.066) | 0.9244 |  |
| Hypertension | WC | 112 | MVMR-IVW | 1.969 (1.635, 2.372) | 8.80E-13 | 0.46 |
|  |  | 112 | MVMR-Egger | 2.223 (1.534, 3.221) | 2.45E-05 |  |
|  | C-reactive protein | 112 | MVMR-IVW | 1.017 (0.937, 1.104) | 0.6894 |  |
|  |  | 112 | MVMR-Egger | 1.014 (0.934, 1.101) | 0.744 |  |
|  | TNF-α | 112 | MVMR-IVW | 0.966 (0.911, 1.023) | 0.2336 |  |
|  |  | 112 | MVMR-Egger | 0.965 (0.910, 1.022) | 0.2226 |  |
|  | IL-6 | 112 | MVMR-IVW | 0.960 (0.909, 1.014) | 0.1444 |  |
|  |  | 112 | MVMR-Egger | 0.959 (0.908, 1.013) | 0.1372 |  |
| Infections of the skin and subcutaneous tissue | WC | 112 | MVMR-IVW | 1.429 (1.179, 1.732) | 0.0003 | 0.978 |
|  |  | 112 | MVMR-Egger | 1.423 (0.970, 2.087) | 0.0714 |  |
|  | C-reactive protein | 112 | MVMR-IVW | 0.989 (0.909, 1.077) | 0.7994 |  |
|  |  | 112 | MVMR-Egger | 0.989 (0.908, 1.077) | 0.8023 |  |
|  | TNF-α | 112 | MVMR-IVW | 1.037 (0.977, 1.101) | 0.2288 |  |
|  |  | 112 | MVMR-Egger | 1.037 (0.977, 1.101) | 0.2288 |  |
|  | IL-6 | 112 | MVMR-IVW | 1.026 (0.970, 1.086) | 0.3683 |  |
|  |  | 112 | MVMR-Egger | 1.026 (0.970, 1.086) | 0.3681 |  |
| Peripheral atherosclerosis | WC | 112 | MVMR-IVW | 1.921 (1.426, 2.586) | 1.71E-05 | 0.641 |
|  |  | 112 | MVMR-Egger | 2.171 (1.197, 3.935) | 0.0107 |  |
|  | C-reactive protein | 112 | MVMR-IVW | 1.033 (0.906, 1.178) | 0.6284 |  |
|  |  | 112 | MVMR-Egger | 1.030 (0.902, 1.176) | 0.6628 |  |
|  | TNF-α | 112 | MVMR-IVW | 0.935 (0.853, 1.026) | 0.1562 |  |
|  |  | 112 | MVMR-Egger | 0.934 (0.852, 1.025) | 0.1523 |  |
|  | IL-6 | 112 | MVMR-IVW | 0.939 (0.861, 1.025) | 0.1579 |  |
|  |  | 112 | MVMR-Egger | 0.938 (0.860, 1.024) | 0.154 |  |
| Sleep apnea syndrome | WC | 109 | MVMR-IVW | 2.475 (2.025, 3.024) | 8.09E-19 | 0.606 |
|  |  | 109 | MVMR-Egger | 2.583 (1.994, 3.345) | 6.40E-13 |  |
|  | C-reactive protein | 109 | MVMR-IVW | 1.003 (0.916, 1.098) | 0.9512 |  |
|  |  | 109 | MVMR-Egger | 1.016 (0.916, 1.126) | 0.7665 |  |
|  | TNF-α | 109 | MVMR-IVW | 1.005 (0.939, 1.075) | 0.8937 |  |
|  |  | 109 | MVMR-Egger | 1.005 (0.939, 1.075) | 0.8956 |  |
|  | IL-6 | 109 | MVMR-IVW | 0.976 (0.917, 1.039) | 0.4507 |  |
|  |  | 109 | MVMR-Egger | 0.976 (0.917, 1.039) | 0.4448 |  |
| Serum uric acid | WC | 109 | MVMR-IVW | 0.218 (0.135, 0.301) | 2.78E-07 | 0.238 |
|  |  | 109 | MVMR-Egger | 0.177 (0.069, 0.284) | 0.0014 |  |
|  | C-reactive protein | 109 | MVMR-IVW | 0.069 (0.031, 0.107) | 0.0005 |  |
|  |  | 109 | MVMR-Egger | 0.056 (0.012, 0.100) | 0.0127 |  |
|  | TNF-α | 109 | MVMR-IVW | 0.009 (−0.019, 0.036) | 0.5362 |  |
|  |  | 109 | MVMR-Egger | 0.009 (−0.019, 0.036) | 0.5326 |  |
|  | IL-6 | 109 | MVMR-IVW | 0.011 (−0.015, 0.037) | 0.4008 |  |
|  |  | 109 | MVMR-Egger | 0.012 (−0.014, 0.037) | 0.3794 |  |
| Gastroesophageal reflux disease | TFP | 103 | MVMR-IVW | 2.275 (2.016, 2.567) | 1.44E-40 | 0.004 |
|  |  | 103 | MVMR-Egger | 2.007 (1.738, 2.317) | 2.28E-21 |  |
|  | C-reactive protein | 103 | MVMR-IVW | 0.988 (0.938, 1.041) | 0.6546 |  |
|  |  | 103 | MVMR-Egger | 0.951 (0.899, 1.006) | 0.0825 |  |
|  | TNF-α | 103 | MVMR-IVW | 1.030 (0.992, 1.071) | 0.1262 |  |
|  |  | 103 | MVMR-Egger | 1.033 (0.995, 1.072) | 0.0901 |  |
|  | IL-6 | 103 | MVMR-IVW | 0.997 (0.960, 1.036) | 0.8888 |  |
|  |  | 103 | MVMR-Egger | 1.001 (0.964, 1.039) | 0.9626 |  |
| Hypothyroidism | HC | 113 | MVMR-IVW | 1.308 (1.124, 1.522) | 0.0005 | 0.211 |
|  |  | 113 | MVMR-Egger | 1.250 (1.058, 1.477) | 0.0088 |  |
|  | C-reactive protein | 113 | MVMR-IVW | 1.065 (0.981, 1.157) | 0.1345 |  |
|  |  | 113 | MVMR-Egger | 1.036 (0.943, 1.138) | 0.459 |  |
|  | TNF-α | 113 | MVMR-IVW | 1.020 (0.965, 1.078) | 0.4774 |  |
|  |  | 113 | MVMR-Egger | 1.025 (0.969, 1.083) | 0.3902 |  |
|  | IL-6 | 113 | MVMR-IVW | 0.988 (0.929, 1.050) | 0.6941 |  |
|  |  | 113 | MVMR-Egger | 0.992 (0.933, 1.055) | 0.8085 |  |
| Varicose veins | HC | 137 | MVMR-IVW | 1.701 (1.442, 2.005) | 2.61E-10 | 0.376 |
|  |  | 137 | MVMR-Egger | 1.968 (1.369, 2.830) | 0.0003 |  |
|  | C-reactive protein | 137 | MVMR-IVW | 0.957 (0.873, 1.048) | 0.3407 |  |
|  |  | 137 | MVMR-Egger | 0.948 (0.864, 1.041) | 0.2629 |  |
|  | TNF-α | 137 | MVMR-IVW | 1.058 (0.998, 1.122) | 0.0601 |  |
|  |  | 137 | MVMR-Egger | 1.054 (0.993, 1.119) | 0.0824 |  |
|  | IL-6 | 137 | MVMR-IVW | 1.038 (0.978, 1.103) | 0.2194 |  |
|  |  | 137 | MVMR-Egger | 1.039 (0.978, 1.103) | 0.2178 |  |
| Acute pancreatitis | HC | 113 | MVMR-IVW | 1.199 (0.943, 1.525) | 0.1387 | 0.779 |
|  |  | 113 | MVMR-Egger | 1.180 (0.904, 1.540) | 0.2232 |  |
|  | TNF-α | 113 | MVMR-IVW | 1.017 (0.931, 1.111) | 0.7085 |  |
|  |  | 113 | MVMR-Egger | 1.019 (0.932, 1.114) | 0.6856 |  |
|  | IL-6 | 113 | MVMR-IVW | 1.020 (0.926, 1.124) | 0.6873 |  |
|  |  | 113 | MVMR-Egger | 1.022 (0.927, 1.127) | 0.6647 |  |
|  | C-reactive protein | 113 | MVMR-IVW | 1.089 (0.957, 1.238) | 0.1952 |  |
|  |  | 113 | MVMR-Egger | 1.078 (0.931, 1.248) | 0.3137 |  |
| Gastroesophageal reflux disease | HC | 110 | MVMR-IVW | 1.694 (1.514, 1.895) | 4.10E-20 | 0.021 |
|  |  | 110 | MVMR-Egger | 1.596 (1.414, 1.802) | 4.13E-14 |  |
|  | TNF-α | 110 | MVMR-IVW | 1.008 (0.968, 1.050) | 0.7038 |  |
|  |  | 110 | MVMR-Egger | 1.014 (0.973, 1.055) | 0.5144 |  |
|  | IL-6 | 110 | MVMR-IVW | 1.007 (0.962, 1.053) | 0.7769 |  |
|  |  | 110 | MVMR-Egger | 1.013 (0.969, 1.059) | 0.5707 |  |
|  | C-reactive protein | 110 | MVMR-IVW | 1.020 (0.959, 1.084) | 0.5298 |  |
|  |  | 110 | MVMR-Egger | 0.983 (0.918, 1.052) | 0.6126 |  |
| Gestational diabetes | HC | 137 | MVMR-IVW | 1.624 (1.228, 2.148) | 0.0007 | 0.762 |
|  |  | 137 | MVMR-Egger | 1.491 (0.804, 2.766) | **0.2051** |  |
|  | C-reactive protein | 137 | MVMR-IVW | 0.969 (0.830, 1.130) | 0.6881 |  |
|  |  | 137 | MVMR-Egger | 0.974 (0.831, 1.141) | 0.7437 |  |
|  | TNF-α | 137 | MVMR-IVW | 1.035 (0.937, 1.144) | 0.4969 |  |
|  |  | 137 | MVMR-Egger | 1.037 (0.938, 1.148) | 0.4762 |  |
|  | IL-6 | 137 | MVMR-IVW | 0.962 (0.869, 1.066) | 0.4629 |  |
|  |  | 137 | MVMR-Egger | 0.962 (0.869, 1.066) | 0.4631 |  |
| Hypertension | HC | 137 | MVMR-IVW | 1.555 (1.350, 1.791) | 9.61E-10 | 0.878 |
|  |  | 137 | MVMR-Egger | 1.589 (1.162, 2.173) | 0.0037 |  |
|  | C-reactive protein | 137 | MVMR-IVW | 1.028 (0.951, 1.112) | 0.4831 |  |
|  |  | 137 | MVMR-Egger | 1.027 (0.948, 1.113) | 0.5157 |  |
|  | TNF-α | 137 | MVMR-IVW | 0.988 (0.939, 1.039) | 0.6418 |  |
|  |  | 137 | MVMR-Egger | 0.988 (0.938, 1.039) | 0.6311 |  |
|  | IL-6 | 137 | MVMR-IVW | 0.992 (0.942, 1.045) | 0.7605 |  |
|  |  | 137 | MVMR-Egger | 0.992 (0.942, 1.045) | 0.7621 |  |
| Insulin resistance | HC | 111 | MVMR-IVW | 0.154 (0.103, 0.206) | 5.24E-09 | 0.849 |
|  |  | 111 | MVMR-Egger | 0.152 (0.094, 0.210) | 3.28E-07 |  |
|  | TNF-α | 111 | MVMR-IVW | −0.009 (−0.028, 0.010) | 0.3369 |  |
|  |  | 111 | MVMR-Egger | −0.009 (−0.028, 0.010) | 0.3539 |  |
|  | IL-6 | 111 | MVMR-IVW | −0.008 (−0.028, 0.013) | 0.4548 |  |
|  |  | 111 | MVMR-Egger | −0.008 (−0.028, 0.013) | 0.475 |  |
|  | C-reactive protein | 111 | MVMR-IVW | −0.001 (−0.031, 0.028) | 0.9187 |  |
|  |  | 111 | MVMR-Egger | −0.003 (−0.036, 0.030) | 0.8578 |  |
| Peripheral atherosclerosis | HC | 137 | MVMR-IVW | 1.410 (1.101, 1.808) | 0.0066 | 0.236 |
|  |  | 137 | MVMR-Egger | 1.893 (1.097, 3.268) | 0.022 |  |
|  | C-reactive protein | 137 | MVMR-IVW | 1.054 (0.919, 1.210) | 0.4499 |  |
|  |  | 137 | MVMR-Egger | 1.035 (0.900, 1.192) | 0.6272 |  |
|  | TNF-α | 137 | MVMR-IVW | 0.992 (0.908, 1.083) | 0.8533 |  |
|  |  | 137 | MVMR-Egger | 0.984 (0.900, 1.076) | 0.7279 |  |
|  | IL-6 | 137 | MVMR-IVW | 0.977 (0.893, 1.070) | 0.6212 |  |
|  |  | 137 | MVMR-Egger | 0.978 (0.893, 1.070) | 0.6255 |  |
| Serum uric acid | HC | 113 | MVMR-IVW | 0.143 (0.074, 0.214) | 0.0001 | 0.07 |
|  |  | 113 | MVMR-Egger | 0.114 (0.038, 0.191) | 0.0031 |  |
|  | TNF-α | 113 | MVMR-IVW | 0.018 (−0.008, 0.043) | 0.1735 |  |
|  |  | 113 | MVMR-Egger | 0.020 (−0.005, 0.046) | 0.1182 |  |
|  | IL-6 | 113 | MVMR-IVW | 0.014 (−0.014, 0.042) | 0.3295 |  |
|  |  | 113 | MVMR-Egger | 0.017 (−0.011, 0.045) | 0.2306 |  |
|  | C-reactive protein | 113 | MVMR-IVW | 0.077 (0.038, 0.114) | 0.0001 |  |
|  |  | 113 | MVMR-Egger | 0.058 (0.016, 0.101) | 0.0073 |  |

Note: *p-value from MVMR-Egger pleiotropy test. Statistical significance was defined as p < 0.05. Effect size is presented as odds ratio (OR) for binary outcomes and as beta coefficient (β) for continuous outcomes (serum uric acid and insulin resistance). All effect sizes correspond to a 1‑standard deviation (SD) increase in the exposure.

Abbreviations: BMI, body mass index; HC, hip circumference; WC, waist circumference; IL-6, interleukin-6; MVMR, multivariable Mendelian randomization; IVW, inverse variance weighted; TFP, total fat percentage; TNF-α, tumor necrosis factor-alpha.
